# Humoral pregnancy analyses reveal strategies for fetal protection and aberrancy in miscarriage

**DOI:** 10.1016/j.xcrm.2026.102993

**Published:** 2026-08-19

**Authors:** Brandon M. Sie, Yumei Leng, Nadege Nziza, Moriah M. Mitchell, Nicholas E. Webb, Yunjoo Lee, Sunni L. Mumford, Enrique F. Schisterman, H. Benjamin Larman, Boris Julg, Galit Alter, Stephen J. Elledge

**Affiliations:** 1 Division of Genetics, Department of Medicine, Howard Hughes Medical Institute, Brigham and Women’s Hospital, Boston, MA 02115, USA; 2 Department of Genetics, Harvard Medical School, Boston, MA 02115, USA; 3 Ragon Institute of Mass General, MIT, and Harvard, Cambridge, MA 02139, USA; 4 Epidemiology Branch, Division of Intramural Population Health Research, Eunice Kennedy Shriver National Institute of Child Health and Human Development, National Institutes of Health, Bethesda, MD 20892, USA; 5 Departments of Biostatistics, Epidemiology, and Informatics, and Obstetrics and Gynecology Perelman School of Medicine, Blockley Hall, 423 Guardian Drive, Philadelphia, PA 19104-6021, USA; 6 Department of Pathology, Johns Hopkins University School of Medicine, Baltimore, MD 21205, USA

**Keywords:** antibody, antibody repertoire, pregnancy, IgG, IgA, PhIP-seq, Luminex, HLA, miscarriage

## Abstract

The maternal immune system must simultaneously defend against pathogens with tolerance of the antigenically distinct fetus. Despite the central importance of immune modulation during pregnancy, epitope-level analyses of the maternal antibody repertoire remain lacking. Here, we performed high-throughput, multi-isotype longitudinal serology of 119 women from preconception through pregnancy, combining phage display of linear peptides with whole protein-based antigen assays to profile antibody responses to viral and self-antigens. We observed a broad expansion of the maternal IgG repertoire and a relative increase in IgA titers, both tracking with pregnancy progression. These changes may augment transplacental and mucosal immunity in the fetus and newborn, respectively. Notably, pregnancies ending in miscarriage exhibited pronounced IgA titer spikes against both viral and self-antigens unrelated to active infection (sensitivity 14/19, 74%, specificity 95%), indicating an underlying immune perturbation may precede pregnancy loss.

## Highlights

• Longitudinal serology of 119 pregnancies reveals maternal antibody dynamics
• Antibody repertoire diversity increases during pregnancy for many pathogen classes
• Levels of antiviral serum IgA increase relative to matched antiviral IgG or IgM
• Increases in IgA for cross-reactive antigens and HLA are linked to miscarriage

## Graphical abstract

**Figure:**
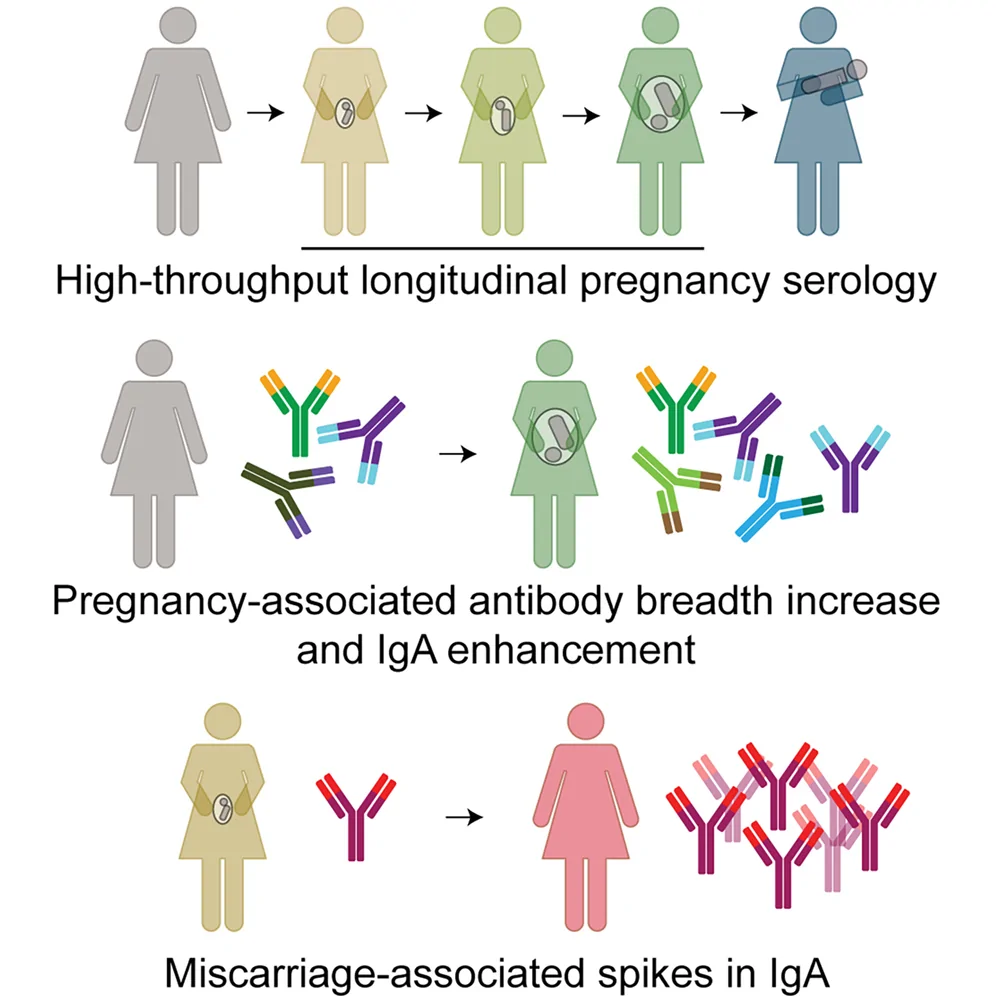

## Teaser

Sie et al. performed high-throughput, longitudinal serology during human pregnancy, revealing maternal immunological dynamics. They found enhancement of maternal humoral immunity over pregnancy, both in antibody diversity and in relative concentrations of IgA. Furthermore, they identified spikes in IgA antigen signals preceding miscarriage, targeting both cross-reactive antigens and HLA.

## Introduction

During pregnancy, expectant mothers’ immune systems must balance generation of effective immune protection for both mother and offspring and prevention complications that might arise from an immune response that targets the fetus. Notably, the developing immune system relies on maternal antibodies to confer passive protection against common pathogens.^1^^,^^2^^,^^3^^,^^4^^,^^5^^,^^6^^,^^7^ To this end, IgG is transferred through the placenta prior to birth, and both IgG and IgA are transferred through breast milk.^3^^,^^4^^,^^5^^,^^8^^,^^9^

Pregnancy has generally been reported to be associated with a decline in maternal antibody titers over gestational age,^10^^,^^11^^,^^12^ which occurs concurrently with an increase in maternal blood volume,^13^ transfer of maternal antibodies across the placenta to the fetus,^3^^,^^5^^,^^6^^,^^14^ and a rise in estrogen and progesterone levels.^15^^,^^16^ Estrogen and progesterone affect the relative frequency of B cell subsets during pregnancy, resulting in a decline in naive B cells and an increase in plasma cells.^1^^,^^16^ These changes in maternal B cells contributed to speculation that expectant mothers may experience changes in their susceptibility to infections or reduced response to vaccination.^3^^,^^17^^,^^18^^,^^19^

While analyses of immune changes during pregnancy have been performed,^1^^,^^20^^,^^21^^,^^22^^,^^23^^,^^24^^,^^25^ a systematic understanding of the breadth and dynamics of gestational immunity at the epitope level is still lacking. Some recent studies have increased the resolution and throughput with which the depth of the maternal antibody repertoire can be profiled. However, these approaches have typically prioritized a relatively focused selection of antigens.^26^^,^^27^ An enhanced understanding of the immune response during pregnancy could inform clinical decision-making, including responding to mid-gestational infections and optimizing the timing of vaccination.^28^ Moreover, there is a need to understand potential differences between pregnancies that end in live birth versus miscarriage. Miscarriage affects 10%–25% of known pregnancies,^29^^,^^30^ and approximately 28% of miscarriages occur after a previously experienced miscarriage.^31^ Although chromosomal abnormalities and maternal age contribute to some miscarriage risk, it is estimated that 30%–50% of miscarriages are from unidentified causes.^32^

## Results

### Longitudinal profiling of antiviral antibody specificity

**Figure 1:**
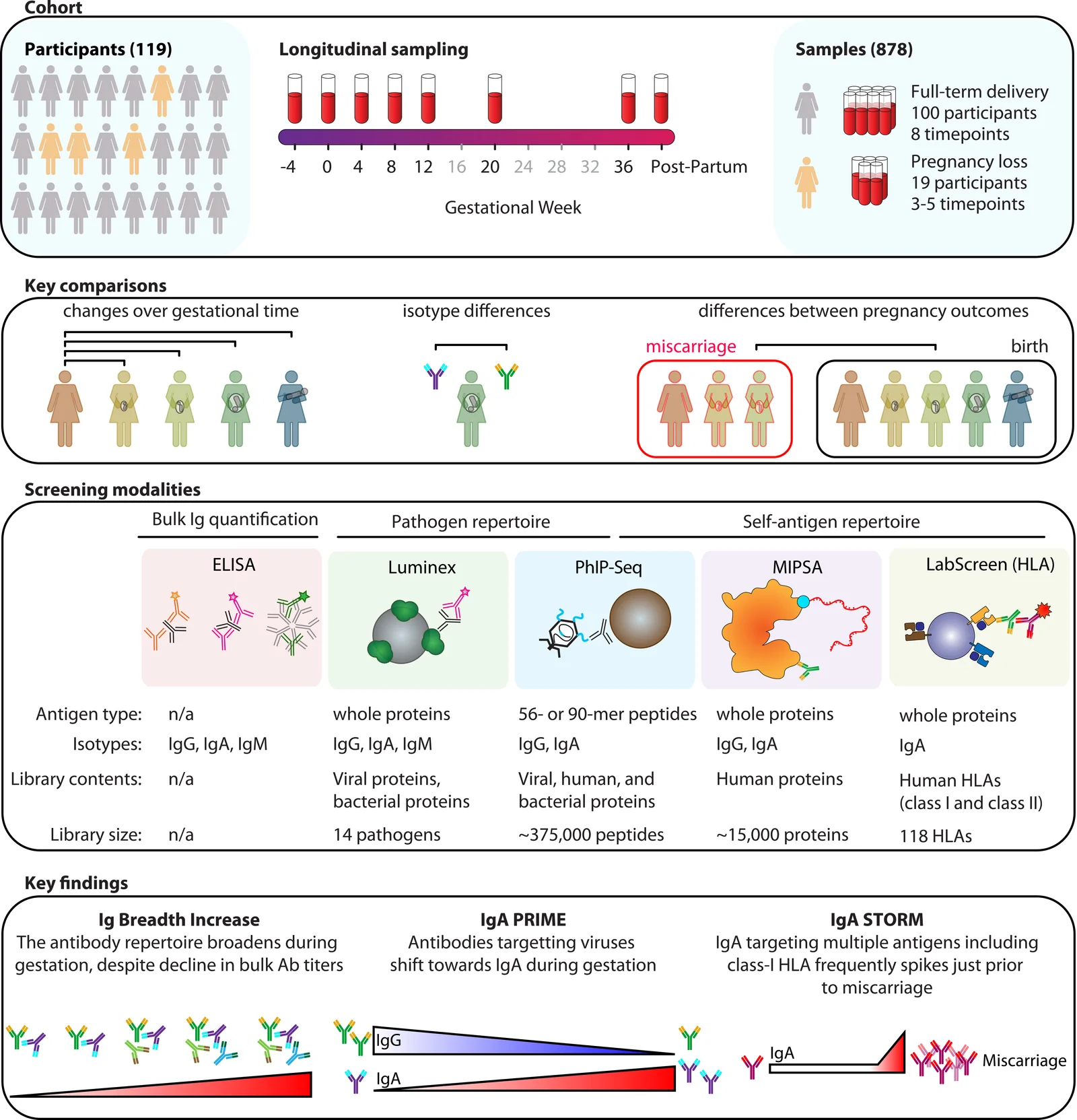
High-throughput, multimodal longitudinal profiling of antibody specificities during pregnancy Overview of the current study. See also Figure S1 and Table S1.

To begin a system level analysis of the architecture of the humoral immune response during pregnancy at the epitope level, we employed longitudinal serum samples for 119 women who participated in the Effect of Aspirin in Gestation and Reproduction (EAGeR) trial^33^^,^^34^^,^^35^^,^^36^^,^^37^ (Figure 1; Table S1). This trial evaluated effects of low-dose aspirin on pregnancy outcomes. Overall, our cohort contains 878 samples, representing 119 pregnancies, of which 100 resulted in full-term births (at gestational age ≥37 weeks), and 19 resulted in miscarriage between gestational weeks 8 and 15 (Figure 1; Figure S1A; Table S1, STAR Methods). Pregnancies not carried to term were followed through the last time point available, typically week 8 or 12, and was collected on average 9.5 ± 7.2 days prior to the estimated pregnancy loss. The birth- and loss-outcome cohorts do not statistically differ in maternal age, maternal BMI, prior pregnancies, prior pregnancy losses, or race (Figure S1A and Table S1). Gestational diabetes (1/119), preeclampsia (8/119), and allergic reaction (5/119) were only observed in the birth-outcome pregnancies. The 100 pregnancies described as “birth outcome” each resulted in a live birth, with typical birthweights (Figure S1A). All miscarriages during the study occurred during the first trimester. None of the 119 pregnancies ended in preterm birth or stillbirth. Overall, ∼17% of pregnancies confirmed by ultrasound in the EAGeR trial ended in miscarriage,^33^ in line with the estimated rate in a general US population of 10%–25%.^29^^,^^30^^,^^38^

Longitudinal antiviral antibody repertoires were examined using orthogonal high-throughput serology profiling technologies: high throughput immunoprecipitation and Luminex. Quantitative anti-viral antibody analyses were chosen because most individuals have previous immunity to hundreds of viral peptides whose analysis can provide great insight into the overall state of the humeral immune system. VirScan, a programmable phage display technology,^39^ was used to systematically profile IgG and IgA antibody specificity using a library of 115,753 phage-displayed peptide epitopes representing ∼400 species and strains of human and several bacterial antigens from Immune Epitope Database (IEDB) (Figure 1; Tables S2 and S3). A custom Luminex assay was performed to profile antibody specificity against 14 common viruses and bacteria proteins, with separate scores for IgG, IgM, IgA, and for antibodies that bind to the neonatal Fc (fragment crystallizable) receptor (FcRn) (Figure 1; Tables S4 and S5). Total IgG, IgM, and IgA titers were also measured using ELISA.

We initially focused on viral epitopes, which are readily detected using VirScan.^39^^,^^40^^,^^41^^,^^42^ Importantly, the humoral immune response to viruses is typical of other antigens,^43^ thus patterns observed in the antiviral antibody repertoire are likely to reflect broader principles of immunity. We used two high-throughput assays to broadly profile potential self-antigens. Molecular indexing of proteins by self-assembly (MIPSA)^44^ was used to profile IgG and IgA repertoires against a library of ∼15,000 human proteins. Additionally, phage immunoprecipitation sequencing (PhIP-seq) with a human peptidome^45^^,^^46^ was performed as an orthogonal method using a peptide library of ∼260,000 peptides representing possible self-antigens. Finally, we performed an additional assay specifically designed to reveal antibody specificities for human leukocyte antigens (HLA) in an allele-specific manner.

Altogether, we profiled ∼146 million interactions between antibody repertoires and peptides by PhIP-seq and ∼2.5 million interactions between antibody repertoires and proteins by Luminex and MIPSA.

### Quantification of antiviral antibody diversity by VirScan

Previous VirScan studies call viral seropositivity based on the number of peptides from a given virus that score as enriched and correlated clinical phenotypes to VirScan data.^39^^,^^40^^,^^41^^,^^42^^,^^47^ This study explored the possibility of extending VirScan’s utility beyond the characterization of specific viral infections. Here, we considered each person’s antibody repertoire across the entire virome library as an indicator of their overall antibody-mediated immune function. We performed this analysis in the context of longitudinal pregnancy samples to examine whether this library-wide paradigm can reveal general insight into the immune response during pregnancy for pregnancies that end in live birth as well as miscarriage.

We quantified peptide enrichment^39^^,^^40^^,^^41^ and verified proper representation of displayed peptides in our phage libraries (Figures S1B–S1E). We confirmed that the antibody repertoire measured by VirScan is stable throughout pregnancy (Figures S1F and S1G). The VirScan library has 56-mer linear peptides tiled every 28 amino acids, so most of the virome is directly represented by two peptides. Additionally, some proteins in the library share homology with others due to viral subfamily relationships and can be clustered into smaller groups of peptides that together represent distinct epitopes (Figure S2A). The number of distinct epitopes identified by VirScan for a given sample is referred to as VirScan “breadth”. It represents the diversity of antiviral antibody specificity within a given sample. After collapsing enriched peptides for sequence similarity (Figure S2B), individuals in this cohort recognized a mean ± SD of 628 ± 191 IgG epitopes per sample (Figure S2C) and a mean ± SD of 651 ± 181 IgA epitopes per sample (Figure S2D), which is in the expected range for a typical healthy adult.^40^^,^^41^

### Enhancement of epitope diversity occurs over gestational time

**Figure 2:**
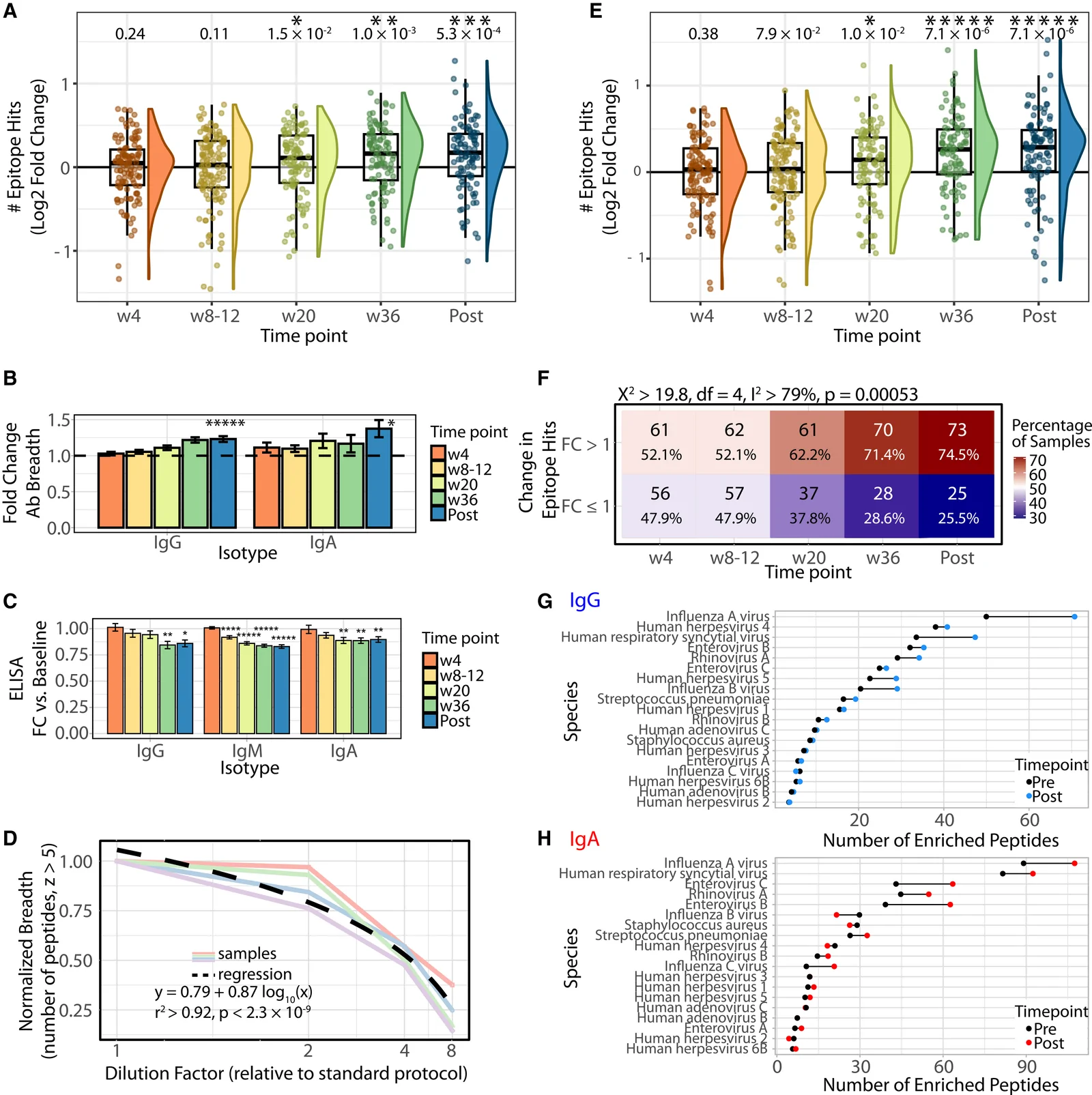
Antiviral antibody diversity broadens during pregnancy (A and E) Boxplots for fold-change in IgG VirScan breadth. Significance values are FDR from a two-sided Wilcoxon rank-sum test comparing median of fold-change of samples at each time point to FC = 1. FDR ∗<0.05, ∗∗<0.01, ∗∗∗<0.001, ∗∗∗∗<0.0001. (A) Without antibody concentration correction (normalization) (E) after adjusting breadth for sample antibody concentration based on the curve generated in (D). Samples were screened in duplicate. (B) Mean fold-change in normalized antibody breadth over pregnancy time by IgG VirScan and IgA VirScan. Error bars indicate standard error of the mean (SEM). FDR-corrected *p* values are shown from one sided *t* tests for the null hypothesis that postpartum breadth fold-change equals 1. FDR ∗<0.1, ∗∗∗∗∗<0.00001. (C) Bar plot of mean change in ELISA IgG, IgM, and IgA titers for the relative to pre-pregnancy baseline. Error bars indicate standard error of the mean (SEM). Asterisks indicate FDRs from one sided *t* tests for the null hypothesis that ELISA fold-change is greater than or equal to 1. FDR ∗∗∗∗∗<0.00001, ∗∗∗∗<0.0001, ∗∗∗<0.001, ∗∗<0.01, ∗<0.05, unlabeled not significant. (D) Effect of serum dilution on IgG VirScan breadth. The dashed curve shows a logarithmic fit to the data. (F) Chi-squared table for samples with breadth fold-change greater than 1 versus less than or equal to 1. (G and H) Number of non-collapsed enriched peptides for selected pathogens, before and after pregnancy for (G) IgG VirScan, (H) IgA. See also Figures S2, S11, and S13, Tables S2, S3, and S10.

VirScan breadth is anticipated to decline over pregnancy due to the increase in blood volume, which dilutes antibody concentration.^13^ We computed a fold-change (FC) in epitope breadth relative to the pre-pregnancy baseline (STAR Methods) for each non-baseline time point. The diversity of the maternal antiviral antibody repertoire is not detectibly changed during the first trimester but is significantly elevated in the second and third trimesters and at the post-partum time point for both IgG and IgA (Figures 2A and 2B). On average, IgG breadth increased by 15.6% (95% confidence interval [CI] 8.3%–23.0%), and IgA breadth increased by 19.6% (95% CI 2.6%–36.6%). VirScan enrichment magnitudes similarly increases over the course of pregnancy (Figure S13). This broadening of specificities occurs despite the expected decline in maternal antibody concentration over gestation, confirmed in this cohort for IgG, IgM, and IgA (Figure 2C; Figure S2G). Some individuals exhibit a decrease in breadth, possibly attributed to an active infection occurring near the start, which resolves with a subsequent decline and apparent drop in breadth at later time points. Nevertheless, the cohort overall shows an increase in VirScan breadth over the course of gestation.

To rule out an artifact due to increase in blood volume during pregnancy, we performed serial dilutions of serum input to VirScan (Figure 2D) which showed that dilution alone resulted in a reliable reduction of breadth. As antibody input into the assay decreases (ranging from 100% of our standard VirScan input to 12.5% of standard), the number of peptides that score as enriched per sample decreases following a logarithmic curve (Figure 2D). This is consistent with prior work.^40^ Thus, the observed increase in maternal antibody repertoire diversity occurs despite declining maternal antibody concentration. Accounting for antibody concentration, IgG breadth increased by 22.4% (95% CI 13.7%–31.0%) (Figure 2E), and IgA breadth increased by 33% (95% CI 11.4%–55.4%). A chi-squared test also indicates a statistically significant change in antibody repertoire breadth over time (Figure 2F). A generalized estimating equations model with an autoregressive AR(1) working correlation structure corroborates significant positive association between gestational time and VirScan breadth, with an estimated 0.59% increase in VirScan breadth per week compared to pre-pregnancy baseline (β = 0.0059, SE = 0.0010, Wald = 35, *p* = 3.4 ×·10^−9^, α = 0.55). We observed broadening of the number of enriched peptides across multiple pathogen classes in both IgG and IgA VirScan, especially among common viruses for which we detect antibody signal by VirScan prior to pregnancy (Figures 2G and 2H). Pregnancy outcome was not associated with differential change in VirScan breadth (Figures S2E and S2F) or different bulk antibody levels by ELISA (Figures S2G and S2H). Receipt of aspirin versus placebo in the EAGeR trial has no measurable effect on VirScan breadth (Figure S2I). Thus, despite decrease in antibody concentration during pregnancy,^10^^,^^11^^,^^12^ and experimental confirmation that the VirScan breadth decreases as antibody concentration decreases, the observed diversity of the maternal antibody repertoire broadens over gestational age.

### Identification of isotype-specific and virus-specific trends in antibody signals by Luminex

VirScan was particularly useful for assessing the overall breadth of the immune response through high-throughput linear epitope profiling. However, we expect most epitopes to be conformational. To complement VirScan and gain deeper insight into the humoral immune system, we employed an orthogonal Luminex-based assay. Luminex was chosen due to its high sensitivity, ability to capture both linear and conformational epitopes, and its ability to readout signals against multiple different antibody isotypes. By Luminex, we profiled antibody specificity against 14 full-length viral protein antigens, chosen for high prevalence and known compatibility with the assay (Figure 1; Tables S4 and S5), with separate readouts for IgG, IgM, IgA, and FcRn binding. Each antigen was conjugated to distinct Luminex beads, and detection antibodies were used to generate fluorescent signals corresponding to the binding of distinct antibody subclasses, as previously described.^5^^,^^26^^,^^48^^,^^49^

**Figure 3:**
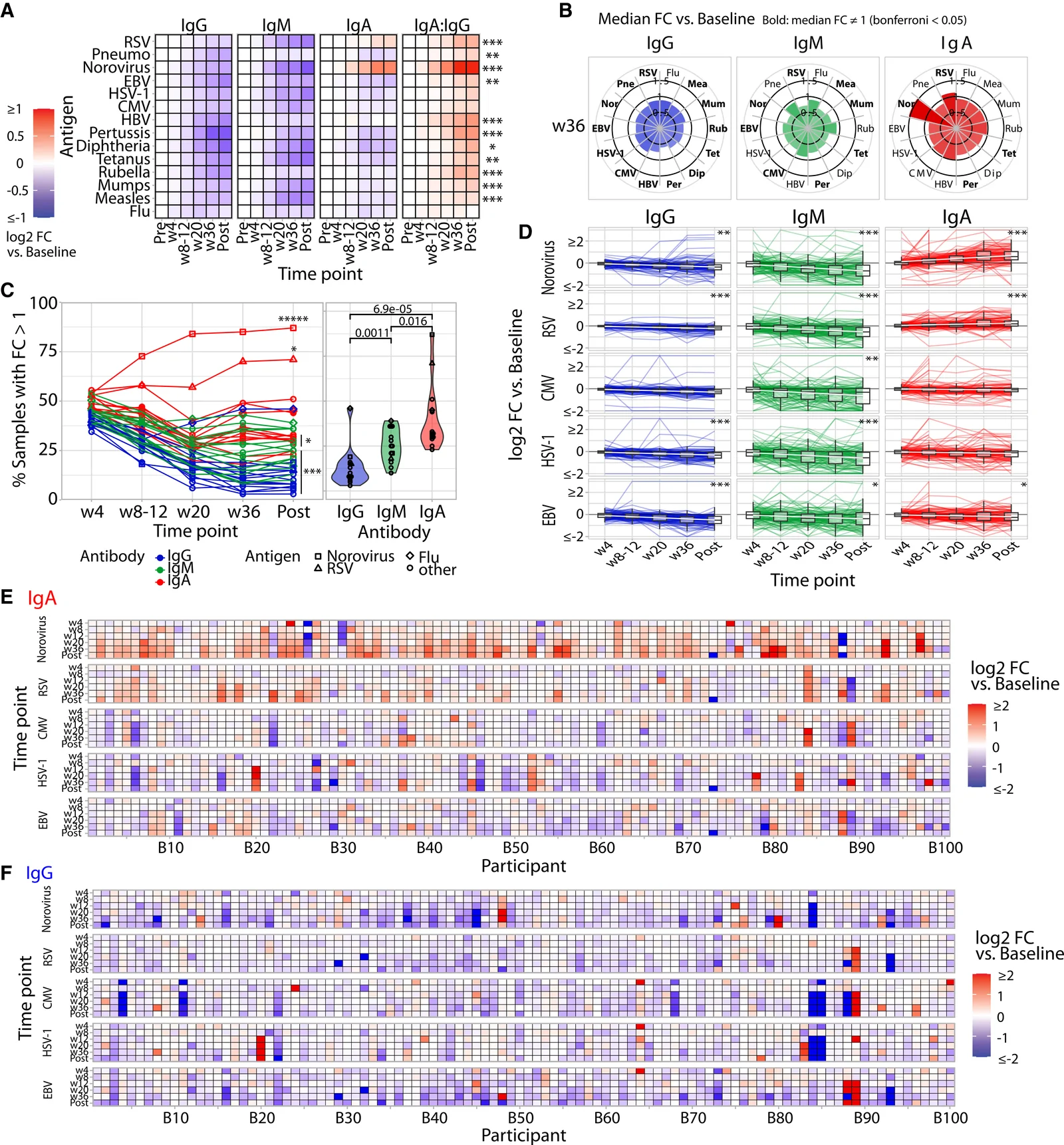
IgA-specific pregnancy-induced maternal enhancement (A) Median fold-change in Luminex MFI compared to pre-pregnancy baseline. The fourth column shows the ratio of median fold-changes for IgA to IgG. Asterisks indicate Bonferroni-corrected *p* values for *t* tests for the null hypothesis that the log2 week 36 IgA:IgG fold-change ratio is equal to 0. ∗∗∗<0.001, ∗∗<0.01, ∗<0.05. (B) Flower plot showing median fold-change in Luminex MFI at week 36 compared to pre-pregnancy. Bolded virus abbreviations indicate that a two-sided Wilcoxon rank-sum test yielded a Bonferroni-corrected *p* value <0.05 for the null hypothesis that median FC = 1. Flu, influenza; Mea, measles; Mum, mumps; Rub, rubella; Tet, tetanus; Dip, diphtheria; Per, pertussis; HBV, Hepatitis B virus; CMV, cytomegalovirus; HSV, 1-herpes simplex virus 1; EBV, Epstein-Barr virus; Nor, norovirus; Pne, streptococcus pneumoniae. (C) (Left) Sample fraction exhibiting fold-change greater than 1 for each Luminex antigen-antibody combination. Asterisks indicate FDR significance values for chi squared tests comparing the number of samples with FC > 1 versus FC ≤ 1. FDR ∗<0.1; ∗∗∗<0.001; ∗∗∗∗∗<0.00001. (Right) Violin plots show percentage increasing scores across all viruses assayed by Luminex. (D) Line plot showing participant Luminex fold-change compared to pre-pregnancy for selected viruses of interest. Boxplots show median, 25^th^ and 75^th^ percentiles, and whiskers spanning up to 1.5 inter-quartile range. Asterisks indicate Bonferroni-corrected *p* values for t-tests for the null hypothesis that the log2 week 36 fold-change is equal to 0. P_corr_ ∗∗∗<0.001, ∗∗<0.01, ∗<0.05. (E and F) Heatmap showing individual fold-change scores for each participant (column) over gestational time (rows) for IgA (E) and IgG (F). See also Figures S3, S4, and S12, Table S4.

For each participant, two pre-pregnancy time points were averaged to establish a baseline, and fold-changes were calculated by comparing Luminex mean fluorescence intensity of each sample to this baseline. Overall, median IgG and IgM titers consistently decline over gestation, while median IgA titers remained relatively stable. Notably, increases in IgA levels were observed for anti-norovirus and anti-RSV (respiratory syncytial virus) antibodies (Figures 3A and 3B; Figure S3A). For several other tested viruses, IgA levels increased relative to IgG or IgM (Figure 3A; Figures S3B and S4B), suggesting that an overall increase in IgA levels outside of anti-norovirus and anti-RSV may be obscured by the dilution effect of maternal blood volume. Significant increases in antigen-specific IgA relative to bulk IgA were observed at the post-partum time point for rubella, RSV, norovirus, mumps, measles, Hepatitis B virus (HBV), flu, and cytomegalovirus (CMV) (Figure S12).

FcRn is important for the transfer of IgG across the placenta from mother to fetus, especially in the third trimester, and that the Fc region of IgG can be modified to encourage preferential transfer of particular IgG antibodies across the placenta.^3^^,^^50^^,^^51^ Since FcRn binds to IgG^52^ and measured maternal antiviral IgG signals tend to decline over gestational time, it is expected that maternal FcRn-binding signal also declines in maternal circulation over gestational time as we observed (Figure S3C).

We also visualized each individual’s fold-change in Luminex signal (Figure 3D; Figures S3C and S3D; Figure S4). Despite variability, at the post-partum time point, 87% of participants had higher measured levels of anti-norovirus IgA than at their pre-pregnancy baseline, and 71% of participants had higher measured levels of anti-RSV IgA than at their pre-pregnancy baseline (Figures 3C–3E), in contrast to other measured antibody-antigen combinations. On average, anti-norovirus IgA titer increased by 62% in this cohort (95% CI 49.1%–78.7%) over the course of pregnancy. Increases in anti-norovirus IgA and anti-RSV IgA titers co-occurred with a decline in corresponding IgG titers (Figures 3E and 3F; Figures S3A and S3D).

To rule out explanations for observed IgA increases involving seasonality, we stratified the longitudinal trajectories by month of conception (Figure S3E). Increases in anti-norovirus IgA titer and anti-RSV IgA titer occur regardless of the month of conception and are, therefore, unlikely to be explained by seasonal waves of infection. Similarly, increases in IgA antiviral titers are not confined to a particular sample collection site and thus do not seem to be explained by a regional viral outbreak (Figure S3F) and are not measurably affected by receipt of aspirin vs. placebo in the original trial. Thus, these data indicate that IgA levels in pregnant women overall do not follow the general decline seen for IgG antibodies and, for some mucosal viruses such as norovirus and RSV, exhibit a phenomenon that we will refer to as IgA pregnancy induced maternal enhancement (IgA PRIME)—an augmented IgA response during pregnancy.

VirScan data also suggested increased IgA signal to norovirus and RSV over gestation (Figures S3G and S3H), which further supports this finding. Public epitope peptides from norovirus capsid protein VP1 and RSV surface glycoproteins, for which whole proteins were used as antigens in Luminex, exhibited increasing enrichment by VirScan over gestational time (Figures S3G and S3H). Thus, we find an IgA-specific increase in certain antiviral antibodies both using linear peptides whole proteins. Together, these data suggest a boosting of an IgA-specific, antigen-specific response to norovirus and RSV during pregnancy.

Clinically determined viral infection status was not available, so we cannot reference clinical infection data for active infection. However, data collected during the EAGeR trial of influenza vaccination aligns well with our serological profiling of anti-influenza antibodies. We observed a heterogeneous influenza response by Luminex (Figure S3I), which is partially explained by the fact that 21 of 119 participants received a flu vaccination during their pregnancy. The vaccinated individuals showed a week-36-versus-baseline fold-change in flu IgG Luminex signal with a mean of 2.74 (95% CI range 2.15–3.33) compared with 1.22 (95% CI range 1.12–1.31) for women who did not receive a documented flu vaccination during pregnancy. This difference is enhanced for FcRn-binding antibodies, for which the same fold-change has a mean of 18.52 (95% CI range 9.99–27.05) for women who received a flu vaccination during pregnancy and a mean of 1.66 (95% CI range 1.42–1.89) for women who did not (Figure S3I).

### Differential patterns of antiviral antibody specificity in pregnancies that end in live birth versus miscarriage

**Figure 4:**
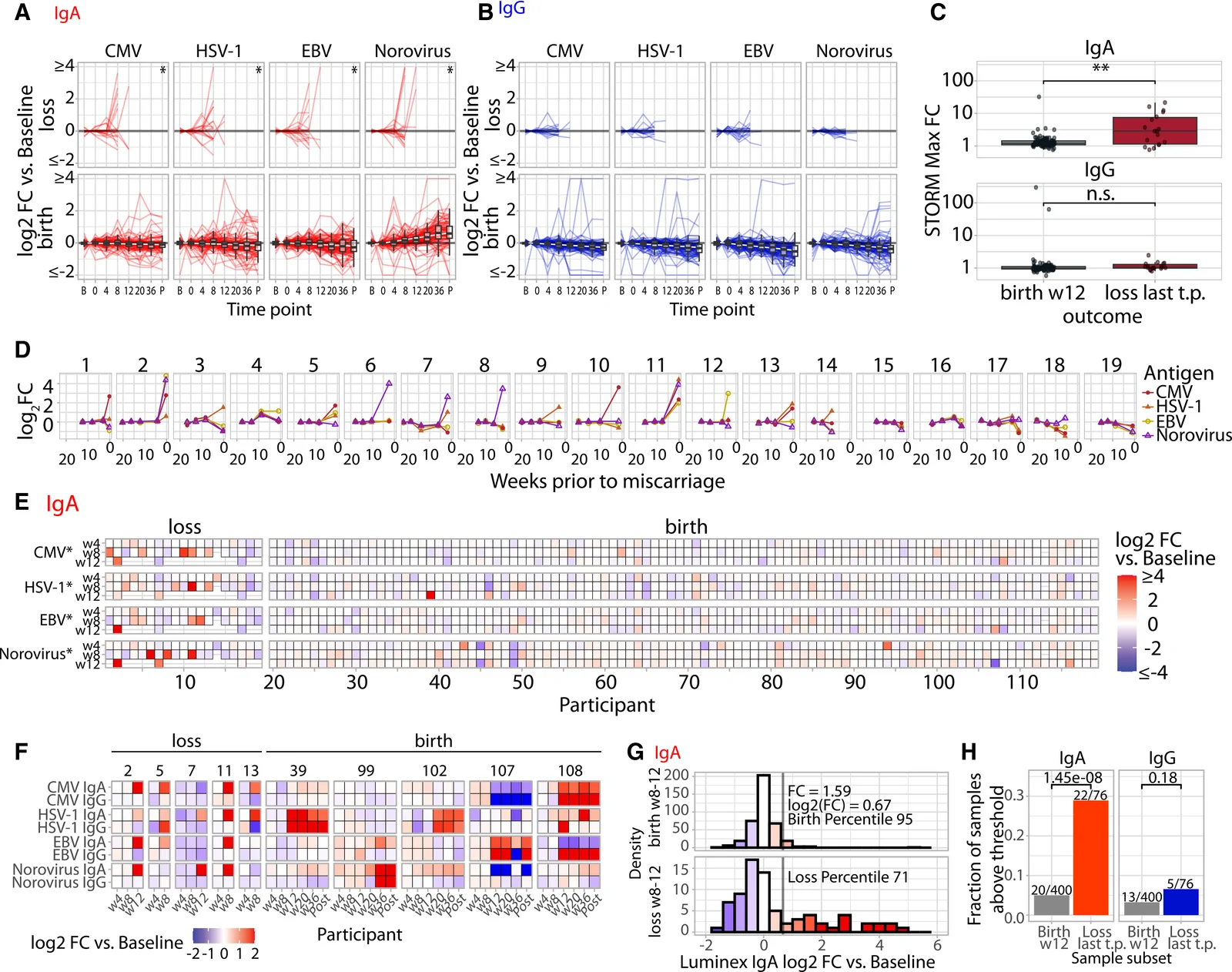
IgA spikes tracking onset of repeat miscarriage (A and B) Line plot split by pregnancy outcome showing participant Luminex log2 fold-change compared to pre-pregnancy for selected viruses for (A) IgA and (B) IgG. X labels indicate time point. B, pre-pregnancy baseline, 0–36 gestational week; P, post-partum. Asterisks indicates that loss-outcome samples at weeks 8–12 were significantly enriched over birth-outcome samples for scores above FC > 2 by Fisher’s exact test. For birth-outcome pregnancies, boxplots show median, 25^th^ and 75^th^ percentiles; whiskers spanning up to 1.5 inter-quartile range; white lines show linear regression fit. (C) Boxplot comparing a simplified IgA STORM metric in pregnancies that ended in livebirth versus miscarriage for IgA (top) and IgG (bottom). For each pregnancy, the highest Luminex fold-change for CMV, HSV-1, EBV, or norovirus was recorded from the last sample prior to pregnancy loss, or from gestational time matched samples from pregnancies that ended in live birth. Significance values indicate the FDR from *t* tests comparing the score distribution for birth-outcome and loss-outcome samples: ∗∗ FDR<0.01, n.s. not significant. (D) Line plots for each of 19 pregnancies that ended in miscarriage, showing Luminex IgA fold-change compared to pre-pregnancy for CMV, HSV-1, EBV, and norovirus. The *x* axis shows the weeks between sample collection and estimated pregnancy loss. (E) Heatmap splitting by pregnancy outcome showing individual log2 fold-change compared to pre-pregnancy baseline for Luminex antibody binding signal for selected viruses of interest for IgA. Asterisks indicates that loss-outcome samples at weeks 8–12 were significantly enriched (FDR<0.1) over birth-outcome samples for scores above FC > 2 by Fisher’s exact test. (F) Selected participant data from (E) and Figures 3E and 3F. Data are displayed for loss-outcome participants with IgA signal increases against more than one of: CMV, HSV-1, EBV, and norovirus. Additionally, data are displayed for birth-outcome participants for whom we observe concurrent increases in IgA and IgG for a particular virus. (G) Distribution of IgA fold-change vs. pre-pregnancy baseline for selected viruses of interest (CMV, HSV-1, EBV, and norovirus) for week 12 birth-outcome pregnancy samples (top) and last time point loss-outcome pregnancy samples (bottom). Vertical line indicates 95^th^ percentile score for birth-outcome (FC = 1.59). The heatmap color scale is the same as used in (C)–(E). (H) Fraction of birth and loss samples scoring above FC of 1.59, for IgA (left) and IgG (right). Brackets are labeled with FDR values from Fisher’s exact test. See also Figures S4–S10 and S14; Table S4.

In the RSV and norovirus IgA data, we observed abundant spikes in antibody levels early in some pregnancies (weeks 8–12) with no data at later time points (Figure 3C). We then identified that spikes were occurring in the last samples collected from pregnancies that would shortly thereafter result in miscarriage. We call this phenomenon IgA STORM (spikes tracking onset of repeat miscarriage). To explore further, individuals’ trajectories in fold-change for antibodies recognizing different viruses were stratified by pregnancy outcome (Figures 4A and 4B). We assessed all antibody-antigen combinations, performed multiple hypothesis correction (STAR Methods), and then limited our subsequent analysis to a subset of pathogens: high-seroprevalence viruses for which there is no vaccine and that had a statistically significant false discovery rate (FDR): CMV, Epstein-Barr virus (EBV), herpes simplex virus 1 (HSV-1), and norovirus.

Several pregnancies ending in miscarriage exhibited a spike in fold-change IgA signal against CMV, HSV-1, EBV, and/or norovirus at the last available time point before the pregnancy ended (Figure 4A). This spike in IgA signal is not accompanied by a corresponding spike in IgG signal (Figure 4B), and therefore a spike in IgA signal tends to correspond to a spike in IgA:IgG ratio (Figure S5A). When comparing the last samples available from pregnancies that ended in miscarriage versus samples from pregnancies that ended in livebirth matched for gestational time, we find a miscarriage-associated, IgA-specific elevation in the maximum Luminex fold-change (Figure 4C) and in number of viruses with a detectible spike (Figure S4C) across these four viruses. These IgA spikes without a corresponding IgG spike are distinct from the expected pattern observed in likely viral infections observed in the birth-outcome, for which we observed concurrent spikes in both IgG and IgA targeting a particular antigen (Figure 4F; Figure S5G).

To visualize the co-occurrence of spike in IgA signal for multiple viruses in a single individual, we plotted heatmaps of isotype-specific fold-change for viruses of interest across gestational age (Figure 4E; Figure S6D), line plots for the CMV, HSV-1, EBV, and norovirus signals for the 19 pregnancies that ended in miscarriage (Figure 4D; Figure S5D), and equivalent line plots for the pregnancies that ended in live birth (Figure S6). Nine of 19 miscarriages exhibited an IgA FC > 4 (above the 99.75^th^ percentile of week 12 birth-outcome IgA fold-change scores) for at least one of CMV, HSV-1, EBV, or norovirus, with some individuals exhibiting a high fold-change against multiple viruses at the same time point. Five additional miscarriages exhibited an IgA FC above the 95th percentile of equivalent scores from week 12 birth-outcome pregnancies (FC > 1.6) (Figure 4G).

Notably, some of the most pronounced IgA STORM events were detected in samples collected in the two days prior to miscarriage, often accompanied by concurrent IgA spikes against multiple viruses (Figure 4D, participant IDs 2 and 11). Among five participants who exhibited an IgA STORM spike against multiple viruses (participant IDs 2, 5, 7, 11, and 13), the mean interval between last sample collection and estimated date of miscarriage was 4.8 days (SD 4.6 days). In contrast, the remaining 14 participants, who underwent miscarriage but did not display multi-virus IgA STORM, had a substantially longer average interval of 16.7 days (ranging up to 40 days). Despite the small sample size (*n* = 19), this difference was statistically significant (two-sided *t* test *p* = 0.023). These findings suggest that IgA STORM may be most detectable within approximately one week of miscarriage, implying that our current data likely underestimate its overall prevalence. Notably, we detect IgA STORM at the last time point preceding miscarriage but not at the second-to-last time point (approximately 4 weeks in between), suggesting that IgA STORM may not be detectable four or more weeks prior to miscarriage. Nevertheless, spikes in recognition of proteins from different viruses seem to foreshadow an adverse outcome for almost three-quarters of the miscarriage-bound pregnancies in this cohort. Of the 14 individuals who experienced a miscarriage for whom an IgA spike was observed, only one (participant ID 12) had a concurrent IgG spike of similar magnitude (Figure S5D).

To confirm statistical enrichment of spikes in antiviral antibody titers preceding miscarriage, we compared the frequency of large increases in Luminex signals across pregnancy outcome. Combining data for CMV, HSV-1, EBV, and norovirus, we observed that the IgA fold-change data from the week 12 birth-outcome samples followed a normal distribution (Figure 4G, top). We defined a threshold for calling an antibody titer spike based on the 95^th^ percentile of scores in the birth distribution, FC > 1.59. For the equivalent distribution of IgA fold-change from the samples just preceding miscarriage, we observed a disproportionate fraction of data exhibiting an IgA fold-change greater than the 95^th^ percentile threshold of the birth-outcome. We then counted the number of samples that scored above or below the IgA FC > 1.59 threshold for week 12 birth-outcome samples and last time point miscarriage outcome samples, performed Fisher’s exact test, and found a significant enrichment in IgA spikes in the miscarriage outcome compared to the birth-outcome (Figure 4H, left). We did not observe a statistically significant difference in IgG spikes between the two populations (Figure 4H, right). Performing the same IgA analysis with samples from all non-baseline time points (week 4 through postpartum) yielded a similarly significant IgA-specific enrichment in titer spikes among pregnancies that ended in miscarriage (Figures S5B and S5C). We further tested internal robustness of IgA STORM to distinguish pregnancy outcome by bootstrapping (Figure S9) and permutation testing (Figure S10) and observe significantly higher IgA fold-change preceding miscarriage compared to control pregnancies (95% confidence interval for difference in means [2.28, 9.27]) by bootstrapping, with our observed difference (+5.6 higher average fold-change preceding miscarriage) generating a permutation test *p* value of 0.0001. This significant difference was only observed for IgA, not IgG. Receipt of aspirin versus placebo in the original trial is not associated with a significant difference in these IgA-specific spikes. Together, these results show that the magnitude of the IgA spikes that we observe preceding miscarriage are higher than can be explained simply by inter-individual variability.

The observed large spikes in fold-change are often associated with low pre-pregnancy levels. The distribution of antigen-specific antibody titers observed by our Luminex screening is often bimodal, and thus, we can draw a threshold line with which a seropositivity call can be made (Figure S5E). The miscarriage samples for which a large fold-change spike is observed often start with low magnitude Luminex MFI at pre-pregnancy, which increases to a level consistent with the “seropositive” mode of our score distributions (Figure S5F). However, in most birth-outcome samples, for these antigens, IgA-negative samples are also IgG-negative (Figure S5F) and IgA spikes tend to occur alongside a concurrent IgG spike (Figure S5G). By contrast, in individuals experiencing miscarriage, we observed spikes in IgA magnitude without corresponding spikes in IgG magnitude (Figure S5G). Additionally, the pre-pregnancy Luminex titers are not significantly lower for any of our tested viruses for the miscarriage-outcome pregnancies compared to the birth-outcome pregnancies (Figure S14), indicating that differences in the frequency of low basal levels of signals between the loss and birth cohorts are not responsible for differences in spike detection.

Regarding the possibility that IgA STORM represents poly-reactive antibodies, we do not find evidence of a repertoire-wide bias toward epitopes with any particular amino acid composition. Since some of antigens are from viruses for which the patient has not been infected, some of the signal is likely due to cross-reactivity for IgA STORM. Regardless of the source, the observed signal remains likely to be meaningful given how strongly it diverges from the inter-individual variation observed in the birth-outcome cohort. Examining the distribution of IgA Luminex scores from pregnancies that ended in live birth, the 95^th^ percentile of birth-outcome fold-change score at trimester 1 (relative to pre-pregnancy) is exceeded by 14 of 19 miscarriage-outcome pregnancies. This suggests a single metric indicator with approximately 74% sensitivity and 95% specificity within this cohort (95% confidence intervals: sensitivity 58.3%–90.0%; specificity 91.9%–98.4%). Statistical analyses further corroborate that the miscarriage outcome pregnancies are enriched for high IgA fold-change scores by Luminex (Figures S9 and S10). This trend matches the patterns we observed on follow-up with anti-HLA and broader anti-self-antigen screening, where high levels of IgA targeting human proteins were observed prior to miscarriage.

### MIPSA and PhIP-seq reveals broader IgA self-antigen recognition preceding miscarriage

We examined the possibility that IgA STORM may correlate with a maternal immune response against human proteins by two orthogonal techniques. The first, MIPSA,^44^ involves ∼15,000 whole protein human antigens, and the second, PhIP-seq, involves the phage display peptidome approach of VirScan but with a ∼260,000 peptide library tiling across human proteins. Based on the IgA-specific pattern preceding miscarriage that we originally observed by Luminex, we chose to specifically explore the hypothesis that pregnancies that end in miscarriage differ from pregnancies that end in livebirth matched for gestational age in their self-antigen repertoires. To this end, we included samples from two time points: week 0 and week 8–12 (typically the last time point available prior to miscarriage) and profiled both IgG and IgA repertoires by both methods.

By MIPSA, we detected a broader range of whole human proteins that are targeted at week 8–12 prior to miscarriage compared to those detected in pregnancies that result in a live birth for IgA, but not for IgG (Figure S7A and Table S6).

From PhIP-seq, we detect a larger set of human peptides that are bound by IgA antibodies from the samples from miscarriage-outcome pregnancies compared to those from birth-outcome pregnancies, without an equivalent difference for IgG (Figure S7B; Tables S7 and S8).

For both MIPSA and PhIP-seq, we checked for consistent enrichment of recurring targets across participants, which would suggest a pathway. While PhIP-seq and MIPSA have both been used to discover autoantigens that are corroborated by orthogonal studies,^44^^,^^45^^,^^53^^,^^54^ such analyses involved enrichment of a consistent self-antigen across multiple representative of a disease class. In contrast, we observed a much more heterogeneous result in this pregnancy cohort, including for individuals who exhibit a spike in self-antigen breadth prior to miscarriage.

These two assays suggest that the IgA repertoires from samples just preceding miscarriage exhibited an increase in self-antigen recognition compared to gestational time-matched samples from pregnancies that ended in live birth.

### Elevated levels of anti-HLA IgA antibodies detected prior to miscarriage

**Figure 5:**
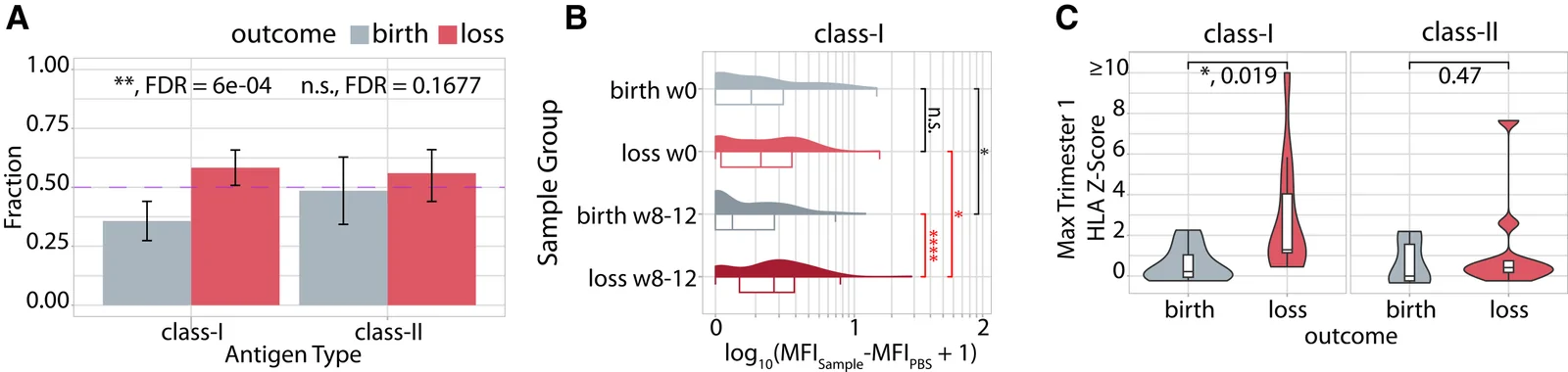
Higher levels of IgA targeting class I human leukocyte antigens detected preceding miscarriage (A) Fraction of measurements with elevated reactivity at week 12 in birth and loss-outcome pregnancies, separated by antigen class. Error bars indicate bootstrap confidence intervals. FDRs correspond to the result of permutation tests comparing the observed difference fraction of elevated scores to the distribution of differences obtained by permuting sample labels (FDR ∗∗< 0.001). (B) Distributions of background-subtracted scores across all tested class-1 HLAs and samples, stratified by pregnancy outcome and time point. Asterisks indicate significance of Wilcoxon tests comparing each group of scores (p ∗∗∗∗<0.0001, ∗<0.1, n.s. not significant). Red brackets with asterisks indicate an increase in anti-HLA signal, either over time or associated with miscarriage, while the black bracket and asterisk indicate a reduction in anti-HLA signal over time in the birth cohort. (C) Box and violin plots showing the maximum trimester 1 population-normalized class-I score for each tested participant. *p* value from a Wilcoxon test comparing the distribution of these scores for birth vs. loss-outcome (FDR ∗<0.05). See also Figure S7; Tables S6, S7. S8, S9, S11, S12, and S13.

Of particular interest in a maternal immune response targeting human proteins are antibodies against HLA, which are associated with prior pregnancy^55^^,^^56^ and miscarriage risk.^57^^,^^58^ We adapted OneLambda LabScreen Mixed, a Luminex-based assay which detects anti-HLA IgG preceding organ transplantation (Figure 5A and Table S9), to detect anti-HLA IgA. We compared pregnancies ending in miscarriage with those ending in birth, matched for gestational age. To this end, we again included samples from two time points: week 0 and week 8–12 (typically the last time point available prior to miscarriage). We observed significantly higher IgA antibodies targeting class I HLA in pre-miscarriage samples from week 8–12 compared with pre-pregnancy that we did not detect in samples from birth-outcome pregnancies (Figure 5A; Figure S7C). Notably, while pregnancies that end in miscarriage exhibit a significant increase in their anti-class I HLA IgA over the first trimester of pregnancy, we observe a subtle but slightly significant decrease in anti-class I HLA IgA over the first trimester of pregnancy for birth-outcome pregnancies (Figure 5B). Additionally, significantly higher levels of IgA antibodies targeting class I HLA were found in pre-miscarriage samples from week 8–12 compared to birth-outcome samples from the same gestational age (Figures 5B and 5C; Figures S7C–S7E).

Presumably, an HLA-directed humoral response would involve antibodies targeting one particular HLA. We sought to overcome lack of HLA typing for this cohort by collapsing our anti-HLA antibody data to a single strongest antigen per person (STAR Methods). Comparing this measure across pregnancies that ended in miscarriage and livebirth, the samples preceding miscarriage exhibited significantly higher maximum anti-HLA IgA scores for class I, but not class II (Figure 5C).

## Discussion

Pregnancy represents a profound alteration of physiological homeostasis. Broad hormonal changes set in motion a series of adaptations to support the growth and development of the fetus in an environment that protects both the fetus and the mother-to-be. Changes to maternal immunity are critical because the mother’s immune system must be tamed to enable tolerance of the fetus, but, at the same time, protect the fetus from infection *in utero* and after birth. In this study, we report a high-throughput, longitudinal, multi-isotype analysis of the antiviral antibody repertoire in 119 pregnant women to understand the antibody response at high resolution using two orthogonal technologies: PhIP-seq and Luminex.

The first major finding from this study involves antibody breadth during pregnancy. Maternal antibody concentrations are known to decrease during pregnancy,^10^^,^^16^ which has been hypothesized to decrease the likelihood of a deleterious immune response against the fetus.^18^ Unexpectedly, we observed that epitope-level antiviral IgG and IgA diversity increases over gestational age. These observations support the notion that maternal B cell immunity is not wholly suppressed during pregnancy, but rather the mother may be employing a deeper recall of immune memory, potentially enhancing protection to the mother and fetus. The magnitude of the antiviral antibody breadth increase observed here is 22% for IgG and 33% for IgA when accounting for change in bulk antibody concentration. The precedence for a change of this magnitude being meaningful comes from the observation that a 20% reduction in the breadth of diversity as a consequence of measles infection may increase the risk of disease from subsequent pathogen exposure.^40^ Accordingly, increasing antibody breadth would be expected to increase protection. Pregnancy causes an intricate cascade of immunological changes, particularly within the T cell compartment.^59^ After implantation, immunity transitions from a Th1 immunity to Th2 dominance, a change that reverses post-partum.^60^^,^^61^ A higher Th2:Th1 ratio favors B cell activation,^62^ consistent with the broader antibody repertoire observed by VirScan.

The second major finding involves the phenomenon that we preliminarily refer to as IgA PRIME. Unlike IgG and IgM levels that decrease during gestation, Luminex and VirScan both showed IgA levels for antigens increased. Although the most striking increases were observed for IgA targeting mucosal pathogens, norovirus and RSV, we also observed relative increases in IgA for many viruses when compared with the bulk IgA level or with antigen-specific IgG or IgM. There are several biological changes during pregnancy that may be involved. As previously mentioned, pregnancy is associated with a shift toward Th2-mediated immunity,^60^ with an environment rich in TGF-β and IL-5 that facilitates class switch recombination to produce IgA.^63^ Furthermore, conversion of IgA memory cells into plasma cells, facilitated by other Th2-associated cytokines, notably IL-6 and IL-10,^64^ could play a role. Similarly, shifts in B cell populations toward greater preference for IgA could be driven by pregnancy-associated changes in the microbiota and hormone levels.^65^^,^^66^ B cells express high levels of estrogen receptor and respond to the elevated levels of estrogen and progesterone characteristic of pregnancy. Monteiro et al. reported that high estrogen and progesterone levels favor expansion of particular follicular helper T (T_FH_) subsets, as well as memory B cells and plasma cells,^16^ findings consistent with our observation of a more robust maternal immune response during pregnancy, as in the increasing antibody breadth and IgA PRIME signals we observe. The possibility that IgA PRIME could be explained by subclinical viral exposures cannot be excluded, although the absence of parallel antigen-specific serum IgG reduces this possibility. We speculate that IgA PRIME could be part of maternal preparation or “priming” for breastfeeding, which is primarily IgA transfer.^8^^,^^9^^,^^67^^,^^68^ Notably, we still see an increase in overall VirScan breadth even if norovirus and RSV are excluded, emphasizing that IgA PRIME and antibody breadth increase are distinct phenomena.

The third major observation concerns distinct antibody repertoire patterns between full-term pregnancies and miscarriages, specifically a phenomenon we preliminarily refer to as IgA STORM. Careful analysis of IgA antibody dynamics revealed sharp spikes in IgA levels recognizing certain viral proteins for many women immediately prior to miscarriage, often to an extent well above that observed at any stage of healthy pregnancies resulting in live birth. Although we initially observed IgA STORM spikes in IgA levels in viral Luminex data, we also found that pre-miscarriage samples in this cohort show significantly higher levels of IgA against HLA class I and overall self-antigens, compared to controls from pregnancies that resulted in birth. Prior pregnancy and miscarriage are both associated with the presence of detectable antibodies against HLA.^55^^,^^56^^,^^57^^,^^58^ These previous studies provide precedent for the concept of a pregnancy termination mechanism involving maternal antibodies recognizing paternal HLA. However, previous associations involved IgG rather than IgA. This work does not establish spikes in IgA as a cause of adverse pregnancy outcomes; rather, it reports a potential signal of immune dysregulation that will require future validation. Although the biological mechanism and physiological significance of IgA STORM remain unclear, its prevalence is striking. Given the multifactorial, heterogeneous nature of miscarriage, a preliminary biomarker that tracks with nearly 75% sensitivity (specificity 95%), even as an internal performance estimate, is of considerable interest for potential follow-up.^69^

One possible explanation for IgA STORM is viral induction of antigen-specific antibody responses that contribute to miscarriage. Viral infection has long been linked to miscarriage^70^^,^^71^^,^^72^^,^^73^ and CMV is associated with adverse outcomes such as stillbirth,^74^^,^^75^ although definitive evidence remains limited for many viruses.^31^^,^^76^^,^^77^ However, infection alone is unlikely to explain these miscarriages for several reasons. First, IgG recognition of the same proteins is not elevated, as would be expected in a typical viral response. Viral infection in the context of impaired IgG production, while possible, is unlikely. For instance, some SARS-CoV-2 infections elicit IgA responses without detectable IgG.^78^^,^^79^^,^^80^^,^^81^ Yet, one IgA STORM case showed robust IgG and IgA responses to flu vaccination, comparable to those in live-birth pregnancies, arguing against a general IgG deficiency. Second, IgA STORM often shows spikes against multiple pathogens per individual, with five of the ten strongest cases targeting two or more viruses. This pattern is inconsistent with typical infections and not observed in full-term pregnancies, where IgA responses to a single virus are usually accompanied by IgG. Together, these observations suggest that IgA STORM is unlikely to result from viral infection. Instead, the IgA reactivity likely reflects cross-reactivity, potentially as part of an aberrant anti-fetal immune response, since many targeted viral antigens show no evidence of actual infection.

A second possible explanation is that the IgA spikes could represent a dysregulated acceleration of pregnancy stage. IgA PRIME and IgA STORM both include IgA-specific effects that may result from aforementioned conditions favorable for IgA class-switching during pregnancy. However, the magnitude of fold-change spikes that we observe is much larger in IgA STORM than in IgA PRIME, even accounting for differences in timing, such that we believe that these phenomena arise from distinct mechanisms.

A third explanation involves an adverse immune response targeting the fetus. Pregnancy is a known risk factor for immunological dysregulation,^81^^,^^82^ including in antiphospholipid syndrome, systemic lupus erythematosus, and rheumatoid arthritis (RA), all of which can complicate pregnancy. Additionally, IgA autoantibodies have been linked to tissue damage. IgA can recruit neutrophils via FcαR1 (CD89) on myeloid cells and has been implicated in diseases, including linear IgA bullous disease (LABD), dermatitis herpetiformis (DH), and RA.^83^ When bound to neutrophils or macrophages, IgA can trigger phagocytosis or cytotoxic responses.^84^^,^^85^ Moreover, IgA and IL-39 have been associated with neutrophil NETosis,^86^^,^^87^ which is known to cause deleterious systemic inflammation in preeclampsia.^88^ Additional pathogenic immune responses may similarly contribute to miscarriage in this cohort. Prior studies of recurrent miscarriage suggest that individuals with a history of prior miscarriage may harbor antibodies against preexisting fetal microchimeric cells, which can contribute to adverse outcomes in subsequent pregnancies.^89^ All participants in the EAGeR cohort had experienced 1 to 2 prior miscarriages, which could prime maladaptive immune responses in latent pregnancies. Of the many possible explanations for IgA STORM considered, we believe that our data best support the hypothesis of a maternal anti-fetal immune response.

### Limitations of the study

Although this study offers epitope-level resolution of maternal B cell immunity, the sample size (*n* = 119) limits statistical power, particularly for subgroup analyses. A larger cohort would enhance the robustness of these findings. Secondly, this study is also limited by the lack of parental and fetal HLA typing. Incorporating comprehensive HLA genotyping in future serological work would help clarify the relationship between anti-HLA IgA and miscarriage risk and allow determination of whether fetal HLA antigens might be targeted. Finally, this study cannot determine causality between the observed IgA STORM and miscarriage. Mechanistic questions remain open about whether elevated IgA responses precede and contribute to pregnancy loss, or whether immunological events triggered by impending miscarriage elicit the IgA response. It remains to be seen whether the mucosal IgA correlates with the presumably monomeric serum IgA detected in this study. Moreover, prior pregnancy losses could also prime the maternal immune system, influencing IgA responses in subsequent pregnancies.

Altogether, this study demonstrates that pregnancy is associated with dynamic remodeling of the maternal humoral immune system, likely to support fetal success, and that disruptions in this process may contribute to adverse outcomes. One key mechanism involves an expansion of antibody breadth, likely reflecting a deeper representation of the mother’s antigen exposure history and potentially enhancing protection for both mother and fetus. Additionally, IgA PRIME may represent an upregulation of IgA production for post-natal transfer to the infant. Together, breadth expansion and IgA PRIME illustrate how gestational immunity reshapes the maternal antibody repertoire, potentially optimizing offspring protection. In contrast, IgA STORM, an abnormal induction of cross-reactive IgA in some women prior to miscarriage, may offer an early clue into the etiology of subsets of pregnancy loss. It is marked by elevated IgA against class I HLA and a broader range of IgA targeting human proteins. Whether IgA STORM is a response to an underlying pathology or a causal factor remains unresolved. Clarifying this will require analyses of the IgA antibodies induced prior to miscarriage to determine if they are detrimental to pregnancy. If causally linked, these findings could inform the development of targeted interventions to prevent immune-mediated pregnancy loss. The antibody repertoire changes observed here likely reflect upstream dynamics, and continued investigation of the humoral immune landscape during pregnancy holds promise for deepening our understanding of these complex and finely tuned immunological adaptations.

## Resource availability

### Lead contact

Requests for further information and resources should be directed to and will be fulfilled by the lead contact, Stephen J. Elledge (selledge@genetics.med.harvard.edu).

### Materials availability

Any reagents that are unique to this study will be made available upon request with a completed materials transfer agreement.

### Data and code availability

• Original antibody repertoire profiling data were deposited to Gene Expression Omnibus as GEO: GSE314505. They are publicly available as of the date of the publication. The DOIs are listed in the key resources table under “Deposited Data” as “Antibody repertoire profiling data” (GEO). Processed PhIP-seq, Luminex, MIPSA, and HLA data, along with library metadata, are available in supplementary tables.
• This paper does not report original code.
• Any additional information required to reanalyze the data reported in this paper is available from the lead contact upon request.

## Acknowledgments

Portions of this research were conducted on the O2 High Performance Compute Cluster, supported by the Research Computing Group, at Harvard Medical School. See https://it.hms.harvard.edu/our-services/research-computing for more information. We thank Michael Mina for helping access the cohort samples and Lindsey Sjaarda (NIH) and Elizabeth DeVilbiss (NIH) for information on the cohorts.

This study was supported by the following sources: the 10.13039/100000011Howard Hughes Medical Institute (S.J.E.). Biospecimens and associated data were provided by the EAGeR trial, an initiative developed through intramural funding from the 10.13039/100009633Eunice Kennedy Shriver National Institute of Child Health and Human Development, and supported by contract no. HHSN267200,603423, HHSN267200,603424, HHSN267200603426, HHSN275200403394C, HHSN275201100002I, and HHSN2750000I.

## Author contributions

Conceptualization, S.J.E., G.A., and B.M.S.; data curation, B.M.S., N.N., Y.L., and N.E.W.; formal analysis, B.M.S.; funding acquisition, S.J.E. and G.A.; investigation, B.M.S., Y.L., N.N., M.M.M., N.E.W., and Y.L., resources, S.J.E. and G.A., supervision, S.J.E., G.A., B.J., and H.B.L.; visualization, B.M.S.; writing – original draft, B.M.S. and S.J.E.; writing – review and editing, B.M.S., S.J.E., G.A., E.F.S., S.L.M., and H.B.L.

## Declaration of interests

S.J.E. is a founder of and hold equity in Tscan Therapeutics, MAZE Therapeutics, Infinity Bio, Inc., and Mirimus, serves on the scientific advisory boards of Infinity Bio, Inc., and T-Scan Therapeutics, none of which affect this work. H.B.L. is a founder and SAB member of Infinity Bio, Inc. S.J.E. and H.B.L. have a patent on the VirScan technology. G.A. is an employee of AstraZeneca.

## STAR★Methods

### Key resources table

**Table:** 

REAGENT or RESOURCE	SOURCE	IDENTIFIER
**Antibodies**		

Goat anti-human IgA-BIOT	Southern Biotech	Cat#2050-08; RRID:AB_2795706

**Biological samples**		

EAGeR trial participant sera	National Institute of Child Health and Human Development	N/A

**Chemicals, peptides, and recombinant proteins**		

Protein A Dynabeads	Thermo Fisher, Invitrogen	Cat#10008D
Protein G Dynabeads	Thermo Fisher, Invitrogen	Cat#10009D
Pierce Streptavidin Beads	Thermo Fisher	Cat#C88817

**Critical commercial assays**		

LabScreen Mixed	One Lambda	Cat#LSM12
HuSight (MIPSA)	Infinity Bio	https://www.infinitybio.com/mipsa/

**Deposited data**		

Antibody repertoire profiling data	This paper	GEO: GSE314505

**Oligonucleotides**		

Primer: IS7: ACACTCTTT CCCTACACGACTCCAGTCAGG TGTGATGCTC	Shrock et al.^90^	N/A
Primer: IS8: GTGACTGGAG TTCAGACGTGTGCTCTTCCGA TCCGAGCTTATCGTCGTCATCC	Shrock et al.^90^	N/A
Primer: IS4: AATGATACGGC GACCACCGAGATCTACACTCTT TCCCTACACGACTCCAGT	Shrock et al.^90^	N/A
Primer: Index: CAAGCAGAA GACGGCATACGAGATNNNN NNNGTGACTGGAGTTCAGACGTGT	Shrock et al.^90^	N/A
Primer: T7-Illumina-READ1-A: TGCTCGGGGATCCAGGAATT CCGCTGCGT	Shrock et al.^90^	N/A

**Software and algorithms**		

Bowtie	Langmead et al.^91^	https://bowtie-bio.sourceforge.net/index.shtml
Samtools	Li et al.^92^	https://www.htslib.org/
DIAMOND	Buchfink et al.^93^	https://github.com/bbuchfink/diamond
R (version 4.2.1)	R Core Team.^94^	https://www.r-project.org/
Targets R package	Landau.^95^	https://books.ropensci.org/targets/
Tidyverse r package	Wickham et al.^96^	https://tidyverse.org/
Tidytable r package	Fairbanks.^97^	https://cran.r-project.org/package=tidytable
Ggpubr r package	Kassambra.^98^	https://rpkgs.datanovia.com/ggpubr/

**Other**		

VirScan library	Xu et al.^39^	N/A
pep 2.2 library	Larman et al.^45^	N/A
pep 4 library	This paper	N/A
Nunc 96-Well Polypropylene DeepWell Plates	Thermo Fisher	Cat#260251

### Experimental model and study participant details

#### Human samples

Serum samples were obtained from 119 women originally participating in the EAGeR clinical trial^33^ from the National Institute of Child Health and Human Development as indicated in the key resources table. As described initially, the Institutional Review Board for each clinical center approved the study, and written informed consent was obtained from participants.^33^ For all participants, two pre-pregnancy time points were included, approximately 1 month apart, representing the two monthly visits immediately preceding a positive pregnancy test. Additionally, samples were included from gestational weeks 4, 8, 12, 20, and 36, along with one sample collected within a few days post-partum, typically within 48h. Of the 119 participants in Cohort 1, 19 had pregnancies that resulted in miscarriage. Samples were included until the latest pregnancy time point available. Anonymized demographic information (age, sex, pregnancy history, etc.) for EAGeR study participants whose samples are included in the present study are provide in Table S1, and analysis of covariates is included in Figure S1A.

### Method details

#### Phage immunoprecipitation and sequencing

The phage immunoprecipitation and sequencing assay was performed as previously described.^39^^,^^40^^,^^41^^,^^45^^,^^46^ T7 bacteriophage was used to display linear peptides representing the VirScan library. Serum samples were evenly diluted with PBS and a constant volume was assayed such that approximately 2 μg of antibody would be included from a typical serum sample. Diluted serum was mixed with peptide-displaying phage and incubated with end-over-end rotation. We used protein A and protein G Dynabeads (Invitrogen) to capture IgG, following the standard protocol. To capture IgA, we used 4 μg of Goat Anti-Human IgA-BIOT antibody overnight (Southern Biotech), followed by 20 μL of Pierce Streptavidin Magnetic Beads (Thermo-Fisher). Then, from each sample, DNA from the immunoprecipitated phage was amplified for the peptide-coding insert. A sample-specific barcode was attached by polymerase chain reaction,^90^ and pooled samples were sequenced using an Illumina NextSeq. Samples were screened in duplicate, and each plate included no-serum mock immunoprecipitations as controls. All samples were screened by IgG VirScan. A subset of samples, including all miscarriage samples and samples from ∼20% of birth-outcome pregnancies were screened by IgA VirScan. The utilized VirScan peptide library contains 115,753 uniquely barcoded 56-mers with 50% overlap between adjacent tiles tiling across proteins from viruses that infect humans. Two human peptidome libraries were used in this study. One contains ∼260,000 90-mer peptides tiling across human proteins with 50% overlap between adjacent tiles as previously described. For a subset of samples, IgG PhIP-Seq with a human peptidome was performed with an augmented version of the library, pep4, containing ∼600,000 90-mer peptide tiles, tiling across the same human proteome but with two barcodes representing each peptide and some additional control sequences. Samples were processed in 96-well plates, and so multiple 96-well plates were required to accommodate the 878 samples. To avoid the possibility that signal changes observed over the course of gestational time could be explained by batch effect, samples from each participant’s time course were always processed on the same 96-well plate. Additionally, VirScan samples were screened in duplicate, and correlation between replicates was performed as quality control. Normalized read counts from VirScan replicates correlated strongly, consistent with previous VirScan studies, with a Pearson correlation mean ± SD 0.85 ± 0.15. Furthermore, a strong correlation was also observed across different time points from the same participant’s time course, far beyond that observed from samples from mismatched individuals (Figures S1F and S1G).

#### Luminex

Antibody quantification was performed as described previously.^26^^,^^48^ Briefly, a multiplexed Luminex assay was used to determine relative titer of antigen-specific isotypes and subclasses using the antigens described in Table S1. Antigens were covalently linked to carboxyl-modified Magplex© Luminex beads using Sulfo-NHS (N-hydroxysulfosuccinimide, Pierce) and ethyl dimethylaminopropyl carbodiimide hydrochloride (EDC). Antigen-coupled microspheres were blocked, washed, resuspended in PBS, and stored at 4°C. To form immune complexes, appropriately diluted plasma (1:100 for IgG2/3, IgA, IgM; 1:500 for IgG1) was added to the antigen-coupled microspheres, and plates were incubated overnight at 4°C, shaking at 700 rpm. The following day, plates were washed with 0.1% BSA 0.02% Tween 20. PE-coupled mouse anti-human detection antibodies (Southern Biotech) were used to detect antigen-specific antibody binding. Fluorescence was acquired using an Intellicyt iQue.

#### Profiling antibodies against HLA

A commercially available kit from OneLambda, “LabScreen Mixed” was purchased. Instead of the provided PE anti-human IgG detection antibody, we used a PE goat anti-human IgA antibody to adapt the assay for detecting IgA antibodies that target HLA. An optimization test was performed using the IgA detection antibody and a concentration series of a pool of week 12 serum samples. Based on this, a serum concentration was chosen and used to screen the remaining serum samples using OneLambda’s protocol.

#### MIPSA

IgG and IgA MIPSA (Molecular indexing of proteins by self-assembly) assays were performed by Infinity Bio as previously described^44^: a library of proteins was tagged with unique DNA barcodes using the HaloTag-HaloLigand interaction. This library was incubated with human serum to allow antibodies to bind, then immunoprecipitated. The DNA barcodes were then amplified and sequenced.

#### ELISA

Plates were coated with 100 ng/mL per well of anti-human IgG, anti-human IgM, or anti-human IgA. Plates were incubated overnight at 4°C and washed in wash buffer (phosphate-buffered saline with 0.05% Tween 20). Plates were blocked with a 5% FBS solution then washed again with wash buffer. Serum samples were diluted and added to the plates. Plates were incubated on ice for 30 min. After incubation, plates were washed, and anti-human IgG, anti-human IgM, or anti-human IgA coupled to horseradish peroxidase (HRP) (Bethyl Laboratories) was added for detection. Plates were incubated for 1 h at room temperature in the dark and washed. The ELISA was developed with TMB and stopped with sulfuric acid. The signal was read at 450 nm.

### Quantification and statistical analysis

#### Data analysis

Read counts from Illumina sequencing were aligned to the VirScan and PhIP-Seq libraries using bowtie^91^ and processed into read counts with samtools.^92^ Peptide enrichment scores were calculated as z-scores as previously described.^40^^,^^41^^,^^42^ Briefly, null distributions were defined by binning peptides into groups of 300 or larger based on abundance in mock immunoprecipitation reactions, and per-sample per-peptide enrichment scores were calculated as the deviation from the mean of the middle 90% of a sample’s scores within each peptide bin. *Z* score thresholds for calling significant enrichment were chosen as Z > 5 for IgG and Z > 10 for IgA, such that roughly 1% of library scores as enriched for an average sample. The peptides that score above these thresholds in both replicates for a sample are termed “hits.” A small number of samples with fewer than 100 enriched peptides (∼0.1% of the library) were considered QC failures and excluded from subsequent analysis. From each sample set of peptide hits, we calculate a non-redundant breadth measure representing the number of distinct epitopes for which a set of peptide hits provides some supporting evidence. Briefly, the library peptides were queries for sequence similarity against each other using DIAMOND,^93^ a network graph was constructed with nodes representing peptide and edges representing alignments between peptides, and the size of the maximal independent set of nodes was computed for each sample.

For some analyses, measurements were normalized per person to pre-pregnancy levels. For each participant, two initial samples (denoted “w0” for a sample taken around the last menses prior to a positive pregnancy test and “Pre” for a sample taken four weeks prior to w0) are averaged to define a pre-pregnancy immune baseline. The mean of these two scores was taken, and then the ratio was calculated between the score at the time point of interest and the pre-pregnancy average. Some ratios were taken with pseudo-counting, for example (A+1)/(B+1), with specific details described where applicable. For LabScreen Mixed assay data, each sample’s readout consisted of median fluorescence intensity for 19 distinct Luminex beads. After checking replicate correlation, replicate data were combined by taking a weighted average of scores for each analyte, weighted by the number of read counts per replicate. Deconvolution of bead readouts to measures for individual HLAs was performed by taking the mean of scores from beads that had that specific HLA protein on its bead surface.

Data processing, statistical analysis, and figure generation were performed in R version 4.2.1,^94^ using packages targets,^95^ tidyverse,^96^ tidytable,^97^ and ggpubr.^98^ Figures were arranged using Adobe Illustrator 2022. The Wilcoxon rank-sum test, Welch’s *t* test, Fisher’s exact test, and chi-squared test were performed as described in the text, with statistical parameters reported in the figure legend corresponding to each analysis. Tests such as Welch’s *t* test and nonparametric tests were chosen to minimize the possibility of data violating test assumptions. Multiple hypothesis correction was performed using the `p.adjust` function and either Benjamini-Hochberg “FDR” correction or Bonferroni correction as specified in the text. Library metadata is provided in Tables S10, S11, S12, and S13.

#### Multiple hypothesis correction for IgA STORM

To evaluate potential statistical significance of IgA STORM with multiple hypothesis correction, we performed Fisher’s exact test for each antibody-antigen combination, comparing week 8–12 samples from pregnancies ending in live birth and miscarriage, based on the 95th percentile of fold-change scores in the birth-outcome, with a one-sided alternative hypothesis that miscarriage is associated with higher frequency of above-threshold scores. We then performed multiple hypothesis correction using the Benjamini-Hochberg method. The associations that remained statistically significant after correction are IgA-Rubella, IgA-Pertussis, IgA-CMV, IgA-HSV-1, IgA-EBV, IgA-norovirus, IgA-pneumo, and IgA-RSV. We noticed more noise in the influenza data, likely related to mid-pregnancy vaccinations. Thus, as a conservative measure, to exclude the effect of vaccination, we focused subsequent analysis on antibody-antigen combinations with significant FDRs for which there are not common vaccines.

#### Collapsing LabScreen HLA data to a single strongest score

For each of 12 beads containing class I antigens and each of 5 beads containing class II antigens, we centered and scaled our anti-HLA scores across our tested population and calculated a *Z* score for each sample. Then for each week 8–12 sample, the maximum *Z* score across all antigens is chosen to represent the strongest indication of anti-HLA IgA response. Then for class I and class II antigens separately, this maximum anti-HLA score is compared across pregnancy outcomes.

#### Z-scores

High-throughput serology on this scale is an area of ongoing development, and we have used our best judgment to adopt responsible practices. The thresholds used in this study for defining peptide enrichment by VirScan/PhIP-Seq and for scoring differences in between pregnancies that end in miscarriage to those that end in live birth have precedent from previous studies. For *Z* score, our lab and others have used a range of *Z* score enrichment thresholds, from Z > 3.5 across duplicates^40^^,^^41^^,^^42^ to Z > 10 without replicates.^53^ We choose a threshold of Z > 5 across duplicates but find similar trends regardless of chosen *Z* score threshold (Figure S11). For calling aberrant IgA signal in samples preceding miscarriage, we used thresholds defined by the 95^th^ percentile of scores in the control population of birth-outcome pregnancies. This 95^th^ percentile threshold has precedent in a previous PhIP-Seq study^53^ and we additionally show that there is a strong difference in the distribution of IgA signal in first-trimester samples preceding miscarriage and first-trimester samples preceding live birth regardless of our chosen threshold (Figure S8).

#### Covariates

Additionally, we assessed available metadata from the EAGeR as possible covariates. We found that participants who received a documented influenza vaccination during pregnancy show a marked increase in anti-influenza antibodies, particularly IgG that binds to the neonatal Fc receptor (FcRn) (Figure S3I). We do not observe a significant association with maternal age, maternal BMI, maternal race, gestational diabetes, preeclampsia, number of prior pregnancies, number of prior miscarriages, birthweight, month of delivery, sample collection site, documented allergic reaction, or documented rash with any of our major findings of antibody breadth increase, IgA PRIME, and IgA STORM. This is partially explained by very low documented incidence of gestational diabetes, preeclampsia, allergy, and rash in our cohort.

#### Asterisks

For some figures, we represent *p*-values adjusted for multiple hypothesis testing with asterisks to reduce visual clutter. The FDR thresholds corresponding to each quantity of asterisks are described in corresponding figure legends. Except where otherwise specified, we use the following FDR thresholds: ∗<0.05, ∗∗<0.01, ∗∗∗<0.001, ∗∗∗∗<0.0001, ∗∗∗∗∗<0.00001.

## Supplemental information

Document S1. Figures S1–S13 and Table S4

Table S1. EAGeR participant information, related to Figures 1, 2, 3, and 4 Demographic and background information about participants from the EAGeR trial whose antibody repertoires were profiled in this study. Column participant_id shows the numeric ID for each participant, corresponding to the labels in Figures 4; Figures S4, and S5. The remaining columns describe the participant age at study enrollment (age), participant body mass index at study enrollment (bmi), whether the participants identify as white (white), number of prior pregnancies (prior_pregnancy_num), number of prior pregnancy losses (loss_num), indication of known flu vaccination during study period (flu_vacc), indication of whether the participant’s pregnancy during the study period ended in miscarriage (loss), indication of whether the participant’s pregnancy during the study period ended in a live birth (lb), birthweight of offspring for live birth pregnancies (bw), gestational age at the end of pregnancy (gest_age), and whether the participant was assigned aspirin (1) or placebo (0) treatment in the original EAGeR trial (tx).

Table S2. VirScan IgG, related to Figure 2 VirScan enrichment data for IgG IPs. “peptide_id” refers to the numeric identifier for the 56-mer peptide from the VirScan library. Other column names indicate the participant’s numeric id and study time point, separated by an underscore. Table values show the per peptide enrichment scores from each sample, given as the mean of the enrichment scores from two replicates, with the value set to zero if either replicate gave an enrichment *Z* score less than 2.5.

Table S3. VirScan IgA, related to Figure 2 VirScan enrichment data for IgA IPs. “peptide_id” refers to the numeric identifier for the 56-mer peptide from the VirScan library. Other column names indicate the participant numeric id and study time point, separated by an underscore. Table values show the per peptide enrichment scores from each sample, given as the mean of the enrichment scores from two replicates, with the value set to zero if either replicate gave an enrichment *Z* score less than 2.5.

Table S5. ELISA and Luminex, related to Figures 2, 3, and 4 ELISA and Luminex serology data. The first two columns, “participant_id” and “tp” indicate the numeric identifier for each participant and the study timepoint, respectively. The remaining columns provide data for a particular assay. Bulk ELISA for IgG, IgM, and IgA are indicated with column names ending in “_Total”. The remaining columns indicate an antibody subclass (IgG, IgM, IgA, or FcRn for antibodies that bind the neonatal Fc receptor), and the whole protein antigen source against which binding was measured, separated by an underscore. Values indicate the fluorescence or colorimetric score from each assay, corrected for background signal.

Table S6. MIPSA IgG and IgA data, related to Figure 5 MIPSA enrichment FDR-corrected p-values for IgG and IgA assays detecting antibodies targeting human proteins. The first five columns show screen metadata: isotype, antigen UniProt accession, protein sequence, gene symbol, gene product name. Additional columns show fold-changes over mock immunoprecipitation background for each sample, with values set to 1 if the BH-corrected *p* value (false discovery rate) was less than 0.001 or if the fold-change was less than 2.

Table S7. Human PhIP-seq IgG data, related to Figure 5 PhIP-seq human peptidome enrichment data for IgG IPs. “id_600k” refers to the numeric identifier for the 90-mer peptide from the human peptidome libraries. Other column names indicate the participant numeric id and study time point, separated by an underscore. Table values show the per peptide enrichment scores from each sample, given as the mean of the enrichment scores from two replicates, with the value set to zero if either replicate gave an enrichment *Z* score less than 1.

Table S8. Human PhIP-seq IgA data, related to Figure 5 PhIP-seq human peptidome enrichment data for IgA IPs. “id_260k” refers to the numeric identifier for the 90-mer peptide from the human peptidome libraries. Other column names indicate the participant numeric id and study time point, separated by an underscore. Table values show the per peptide enrichment scores from each sample, given as the mean of the enrichment scores from two replicates, with the value set to zero if either replicate gave an enrichment *Z* score less than 1.

Table S9. HLA data, related to Figure 5 LabScreen Mixed data for IgA targeting human HLA. The first two columns, “participant_id” and “tp” indicate the numeric identifier for each participant and the study time point, respectively. The next column, “outcome”, indicates whether the sampled pregnancy resulted in live birth or miscarriage (loss). The next column, “bead_name”, indicates the different readouts obtained from LabScreen Mixed, each of which corresponds to a bead with different HLAs conjugated to its surface. The next column, “analyte_class”, categorized the different bead readouts based on whether their conjugated antigens are from HLA class I, HLA class II, or MICA. The next two columns, count_rep1 and count_rep2, represent the number of measurements collected for that sample and analyte. The next two columns, “median_rep1” and “median_rep2” indicate the median fluorescence intensity measured across collected measurements for that sample and analyte for each of two sample replicates. The next column, “score_mean”, represents the average of the two replicate median fluorescence intensities, weighted based on the relative measurement counts. The next column, “pbs_score_minus_pbs”, represents the background signal measured for that analyte from samples where phosphate-buffered saline (PBS) was substituted participant sera. The next column, “score_minus_pbs”, represents the background-corrected score for that sample and analyte, calculated by subtracting the pbs_score from the previous column, floored at zero.

Table S10. VirScan library metadata, related to Figure 2 Peptide metadata for the VirScan library. Column “id” contains a numeric id that corresponds to the “peptide_id” in Tables S2 and S3. “Species” contains the viral species that the peptide was designed from. “peptide” contains the peptide amino acid sequence. “first_50_nt” contains the 50 nucleotide sequence used for alignment from sequencing reads.

Table S11. PhIP-seq 260k (PEP2.2) library metadata, related to Figure 5 Peptide metadata for the 260k peptide PhIP-seq library (pep 2.2). Column “id” contains a numeric id that corresponds to “id_260k” in Table S8. “gene” contains the gene name that the peptide was designed from and “refseq” contains a corresponding accession number. ‘peptide’ contains the peptide amino acid sequence and “first_50_nt” contains the 50 nucleotide sequence used for alignment from sequencing reads.

Table S12. PhIP-seq 600k (PEP4) library metadata, related to Figure 5 Peptide metadata for the 600k peptide PhIP-seq library (pep4). Column “id” contains a numeric id that corresponds to “peptide id” in Table S7. “Fragment” contains the UniProt accession number and numeric fragment ID for that peptide. “peptide” contains the peptide amino acid sequence and “first_50_nt” contains the 50 nucleotide sequence used for alignment from sequencing reads.

Table S13. MIPSA library metadata, related to Figure 5 Antigen metadata for the MIPSA library. “pep_id” is a numeric peptide id, “UniProt_acc” contains the UniProt accession number, pep_aa contains the peptide amino acid sequence, “gene_symbol” describes contains the gene name.

Document S2. Article plus supplemental information
