# Supplementary material for: Humoral pregnancy analyses reveal strategies for fetal protection and aberrancy in miscarriage
Source: Cell Rep Med. 2026 Aug 19;7(9):102993. doi: 10.1016/j.xcrm.2026.102993 (PMC13589508; doi:10.1016/j.xcrm.2026.102993)
Supplement: Supplementary file 1 [file mmc1.pdf]

Cell Reports Medicine, Volume *7*

Supplemental information

Humoral pregnancy analyses reveal strategies for fetal protection and aberrancy in miscarriage

Brandon M. Sie, Yumei Leng, Nadege Nziza, Moriah M. Mitchell, Nicholas E. Webb, Yunjoo Lee, Sunni L. Mumford, Enrique F. Schisterman, H. Benjamin Larman, Boris Julg, Galit Alter, and Stephen J. Elledge

Supplementary Figure S1

A

Pregnancy Outcome	Participants (n)	Age (years)	BMI	Prior Pregnancies (n)	Prior Pregnancy Losses (n)	Birthweight (g)	Gestational Age at End of Pregnancy (weeks)
Birth	100	28.3 ± 4.0 (19.5 -37.9)	24.8 ± 4.7 (17.1 -40.2)	2.3 ± 1.1 (1-7)	1.3 ± 0.5 (1-2)	3435 ± 444 (2393 -4990)	39.2 ± 1.3 (37-44.9)
Loss	19	29.4 ± 4.9 (20.3 -39.8)	25.5 ± 6.2 (17.8 -40.4)	2.3 ± 1.2 (1-5)	1.4 ± 0.5 (1-2)	NA	10.6 ± 1.8 (8-14.6)
Wilcoxon FDR		0.68	0.95	0.95	0.68		

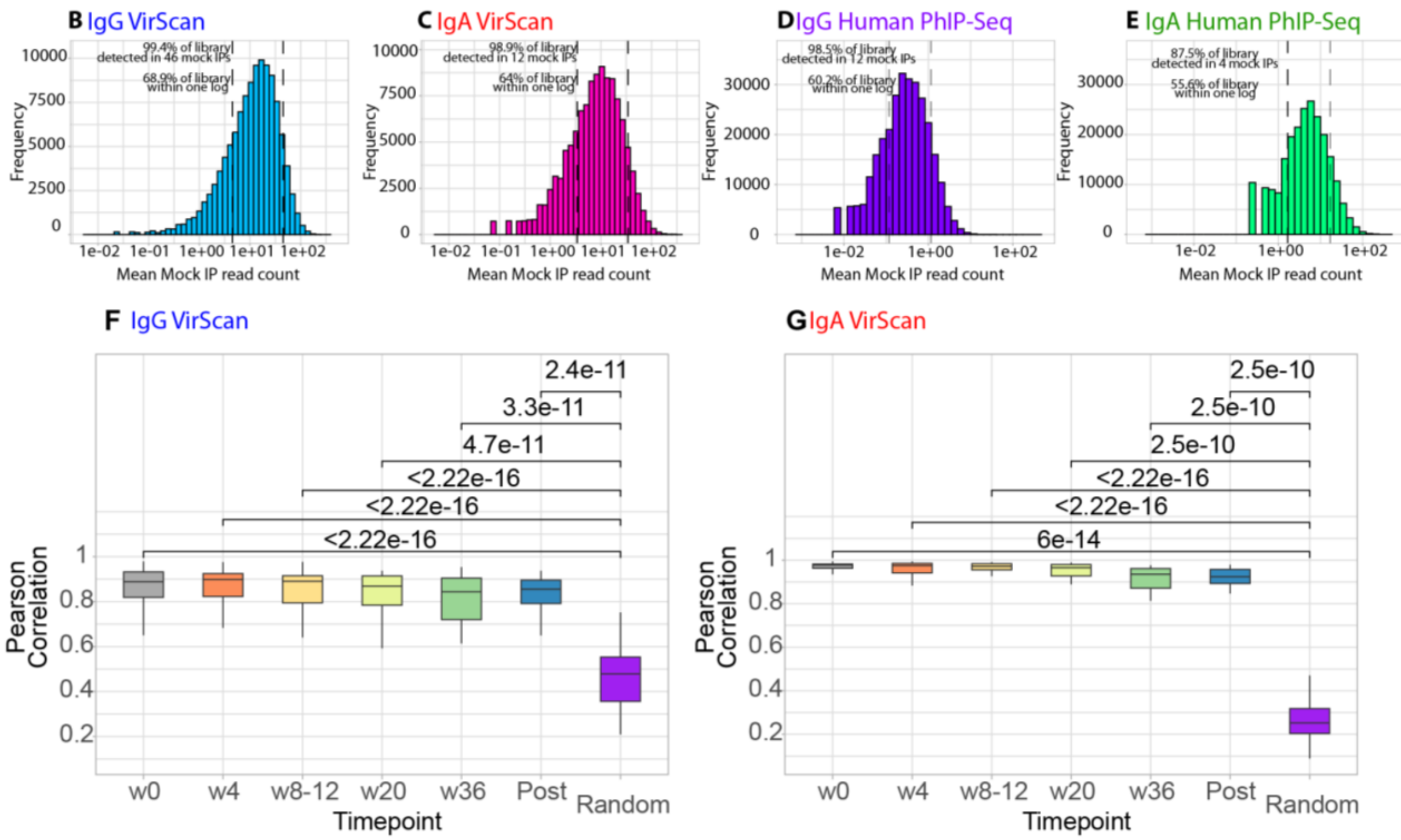

Fig. S1. Sample and library background information. Related to Figure 1.
(**A**) Demographic information for the EAGeR trial participants with samples screened in the current study, separated by pregnancy outcome. Values are listed as "mean ± standard deviation (minimum-maximum)." FDR values at the bottom of the chart show the Benjamini-Hochberg corrected p-values for a Wilcoxon rank-sum test comparing birth and loss outcome values for the metric specified at the top of that column. (**B-E**) Distribution of Illumina sequencing reads, averaged across no-serum mock immunoprecipitation reactions for (B) IgG VirScan, (C) IgA VirScan (D) IgG human PhIP-Seq, (E) IgA human PhIP-Seq. Dotted lines show a one log interval. The percentage of the library observed within one log and across all mock IP reactions are specified. For instances that multiple oligos code for the same peptide sequence, read counts were summed across redundant oligos. (**F-G**) Correlation of reads per million (RPM) for samples across gestational time compared to pre-pregnancy baseline for (F) IgG VirScan and (G) IgA VirScan. The "Random" category is comprised of one comparison each between a pre-pregnancy (Pre) sample and one other pre-pregnancy sample from different participant. P-values indicate the significance of a two-sided Wilcoxon rank-sum test comparing each of the two bracketed populations.

Supplementary Figure S2

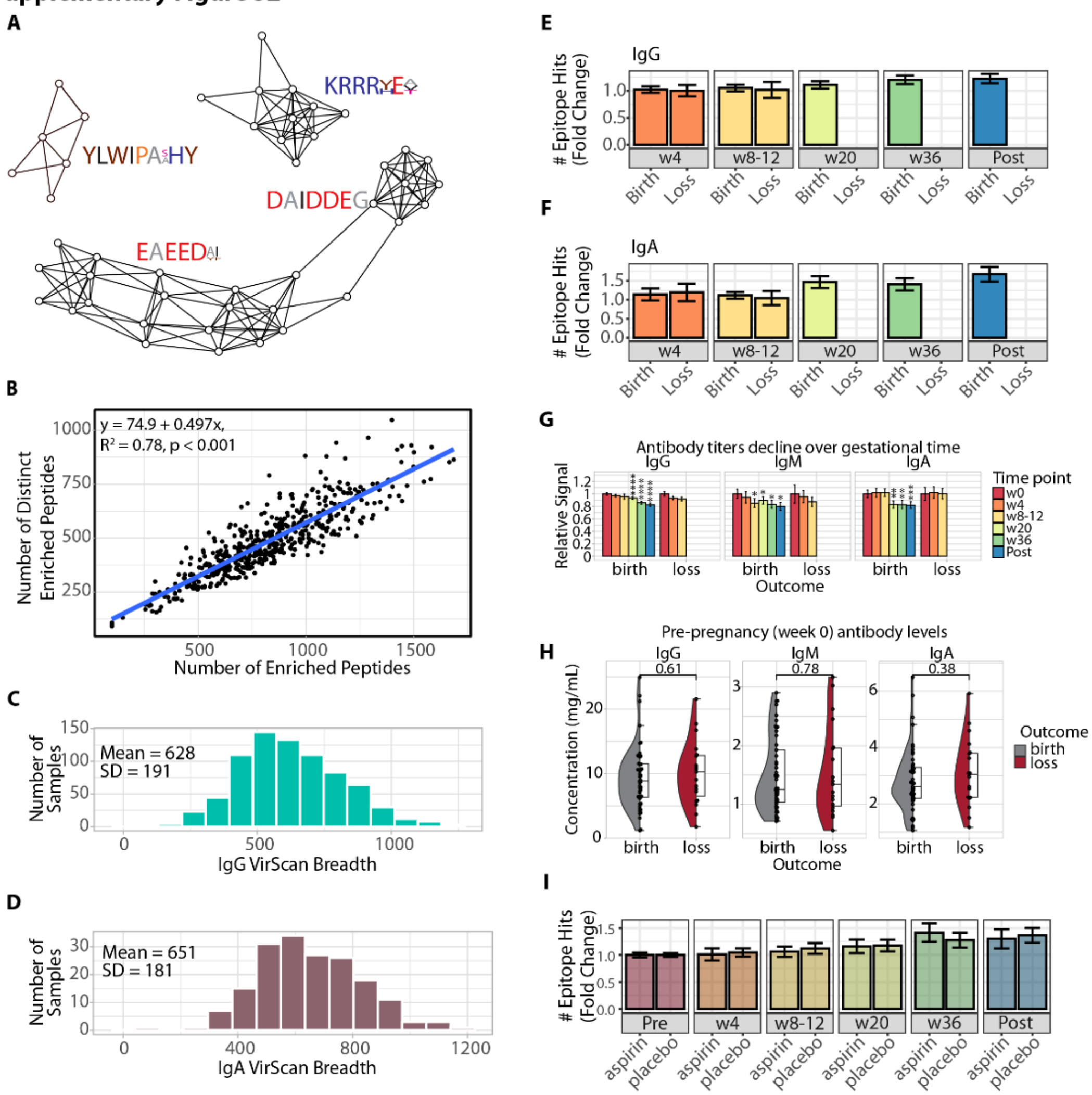

Fig. S2. Features of VirScan breadth quantification. Related to Figure 2.

(**A**) Network graph representation of relationship between peptides and epitopes from VirScan hits. Nodes represent selected peptides enriched by phage immunoprecipitation with the VirScan library for a particular donor sample. Edges represent peptides with sequence similarity. Sequence motif logos represent distinct epitopes, each comprised of a cluster of homologous peptides. (**B**) Scatterplot depicting per-sample non-collapsed number of enriched peptides on the x axis and post-collapse number of distinct enriched peptides on the y axis. The blue line represents a best-fit linear regression, corresponding to the equation, R-squared value, and p-value listed at the top left of the plot. (**C-D**) Distribution of the per-sample number of distinct enriched peptides ("breadth") per sample for (C) IgG and (D) IgA. (**E-F**) Bar plot showing fold change in VirScan breadth compared to pre-pregnancy baseline across gestational time for birth-outcome and loss-outcome pregnancies for (E) IgG and (F) IgA. Error bars indicate standard error of the mean (SEM). (**G**) ELISA measurements for IgG, IgM, and IgA concentration over gestational time, split by pregnancy outcome, normalized to pre-pregnancy baseline. Scores are normalized to the median week 0 scores per isotype and per pregnancy outcome. Error bars indicate SEM. Asterisks indicate significance for a Wilcox test for the null hypothesis that the median relative score at a given timepoint is equal to 1. * FDR < 0.1, ** FDR < 0.05, *** FDR < 0.01, **** FDR < 0.001. (H) Combined violin, box, and scatter plots showing the distribution of pre-pregnancy antibody titers in birth vs. loss outcome samples. Nominal p-values for t-tests comparing loss and birth are shown. (**I**) Bar plot showing fold change in VirScan breadth compared to pre-pregnancy baseline across gestational age for aspirin-receiving and placebo-receiving participants from the original EAGeR trial. Error bars indicate standard error of the mean (SEM).

Supplementary Figure S3

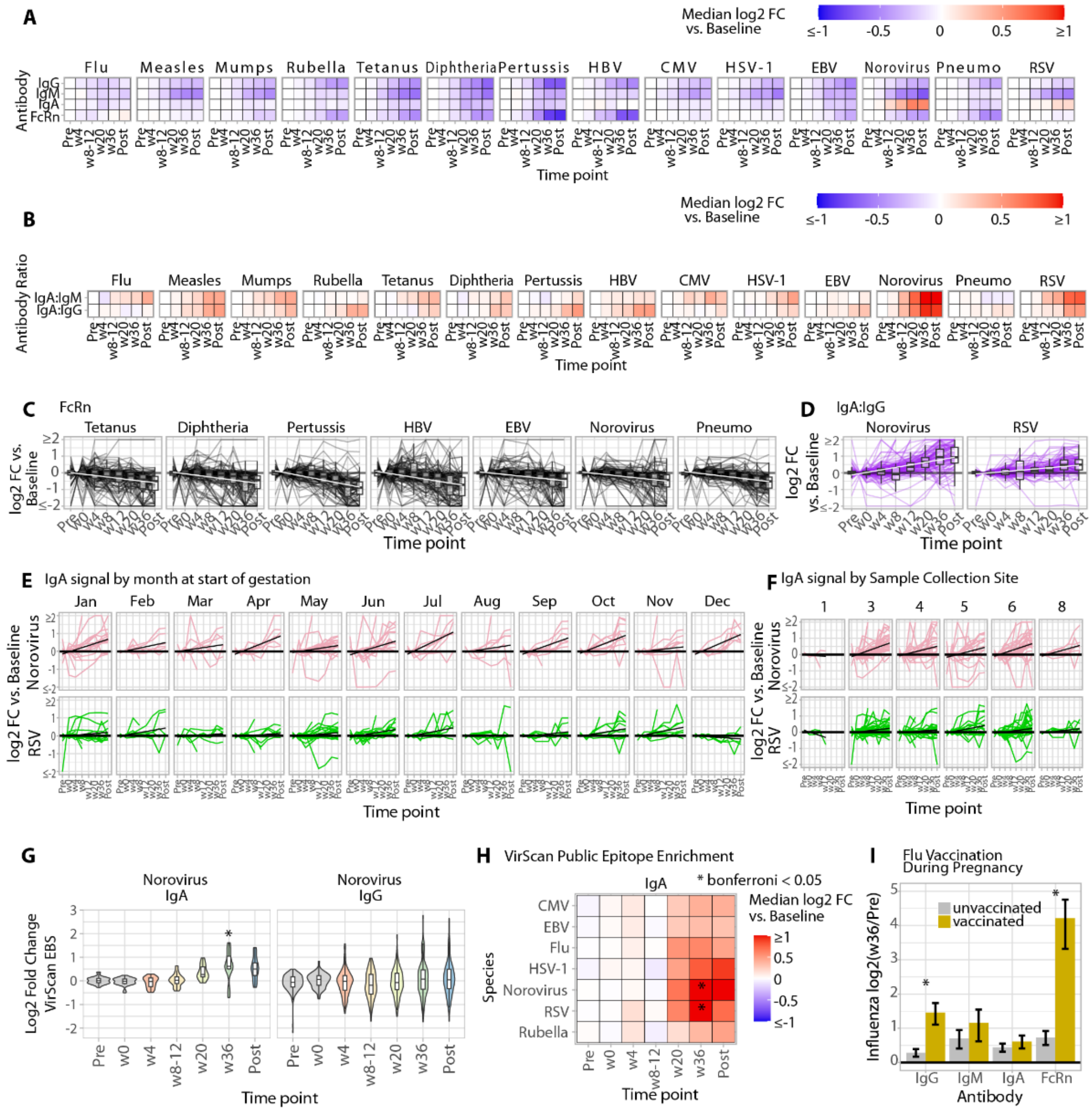

Fig. S3. Trends in Luminex antibody titers over gestational time. Related to Figure 3.

(**A-B**) Median fold change in Luminex MFI compared to pre-pregnancy baseline for antibody binding across all tested antigens. (A) for individual antibody isotypes, or for antibodies that bind to FcRn. (B) for the ratio of signal between two antibody types. (**C-F**) Line plot showing individual participant fold change compared to pre-pregnancy baseline for Luminex. Boxplots show median, 25th and 75th percentiles, and whiskers spanning up to 1.5 inter-quartile range. Trendlines show linear regression fit. (C) FcRn-binding antibodies for selected antigens of interest (D) the ratio of IgA fold change and IgG fold-change for norovirus and RSV (E) IgA:norovirus and IgA:RSV, stratified by month at the start of pregnancy (F) IgA:norovirus and IgA:RSV, stratified by physical location at which sample collection occurred. (**G**) Per-sample mean of IgA and IgG VirScan enrichment scores for 5 norovirus public epitope peptides, plotted as log2 fold change relative to pre-pregnancy baseline. Asterisks indicate Bonferroni-corrected p-value < 0.05. (**H**) Mean for public epitope enrichment score across all samples per time point for IgA for specified viruses. Per-donor public epitope scores were normalized by IgA concentration. Asterisks indicate Bonferroni-corrected p-value < 0.05. (**I**) Luminex influenza fold change at week 36 compared to pre-pregnancy baseline, stratified by antibody subset and whether influenza vaccination was administered during pregnancy. Asterisks indicate FDR < 0.05 for a t-test comparing log2 fold change scores of vaccinated and unvaccinated participants.

Supplementary Figure S4

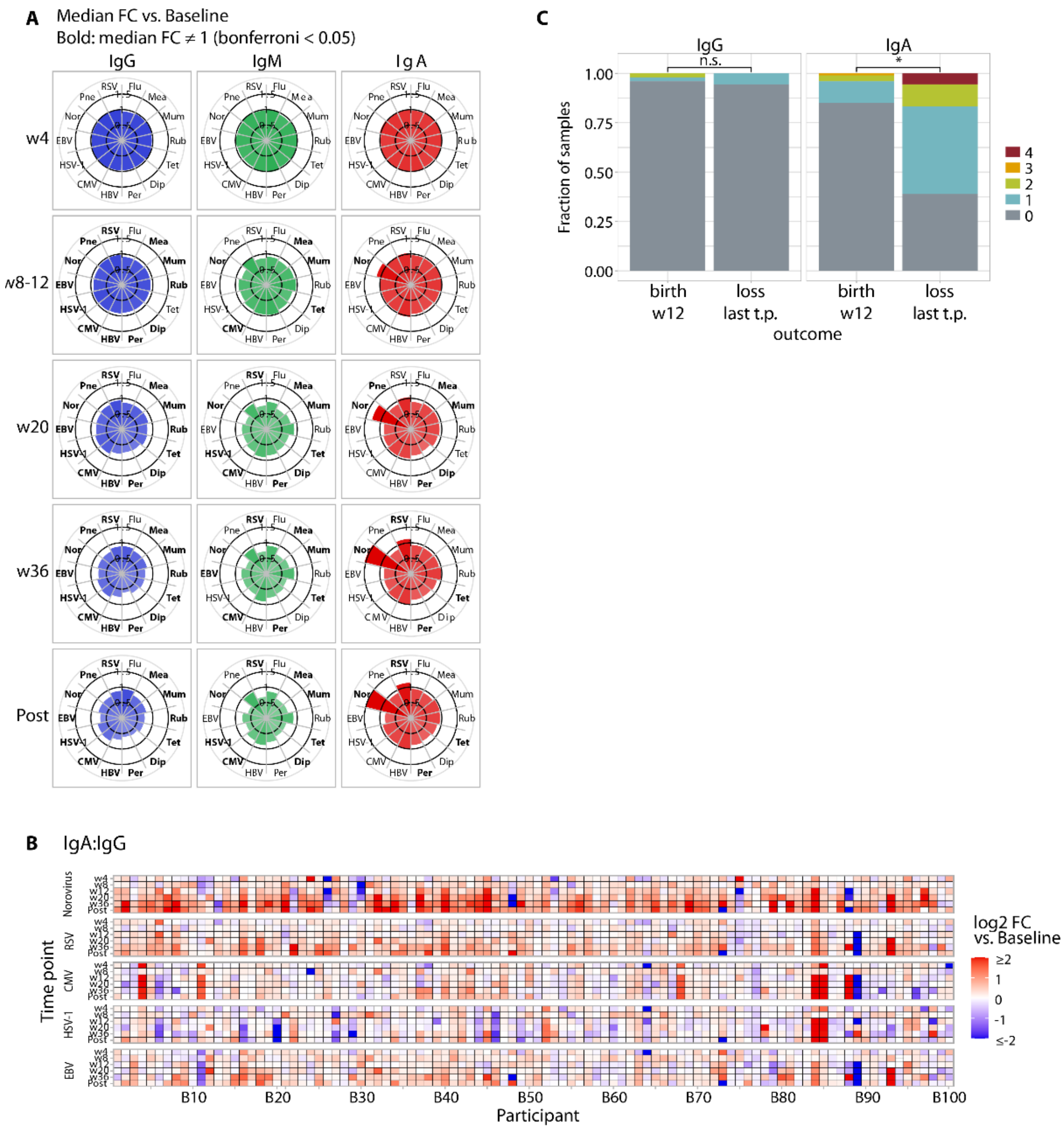

Fig. S4. Luminex antibody fold changes show IgA-specific trends. Related to Figures 3 and 4.

(A) Flower plot showing median fold change in Luminex MFI at compared to pre-pregnancy baseline. Bolded virus abbreviations indicate that a two-sided Wilcoxon rank-sum test yielded a Bonferroni-corrected p-value < 0.05 for the null hypothesis that median FC = 1. Abbreviations: Flu influenza, Mea measles, Mum mumps, Rub rubella, Tet tetanus, Dip diphtheria, Per pertussis, HBV Hepatitis B virus, CMV cytomegalovirus, HSV-1 herpes simplex virus 1, EBV Epstein-Barr virus, Nor norovirus, Pne streptococcus pneumoniae. **(B)** Heatmap showing the individual fold change scores for each participant (column) over gestational time (rows) for the ratio of IgA and IgG. **(C)** Stacked bar plot with colors indicating number of spikes to CMV, HSV-1, EBV, and/or Norovirus per sample. Bar plot expresses the fraction of samples with each number of spikes to these viruses, comparing the last time point available for pregnancies that ended in miscarriage to samples from birth outcome pregnancies that are matches for gestational time. Significance values indicate the FDRs for t-tests comparing per-isotype distributions of number of antiviral spikes in the specified loss- and birth-outcome samples (FDR *<0.05).

Supplementary Figure S5

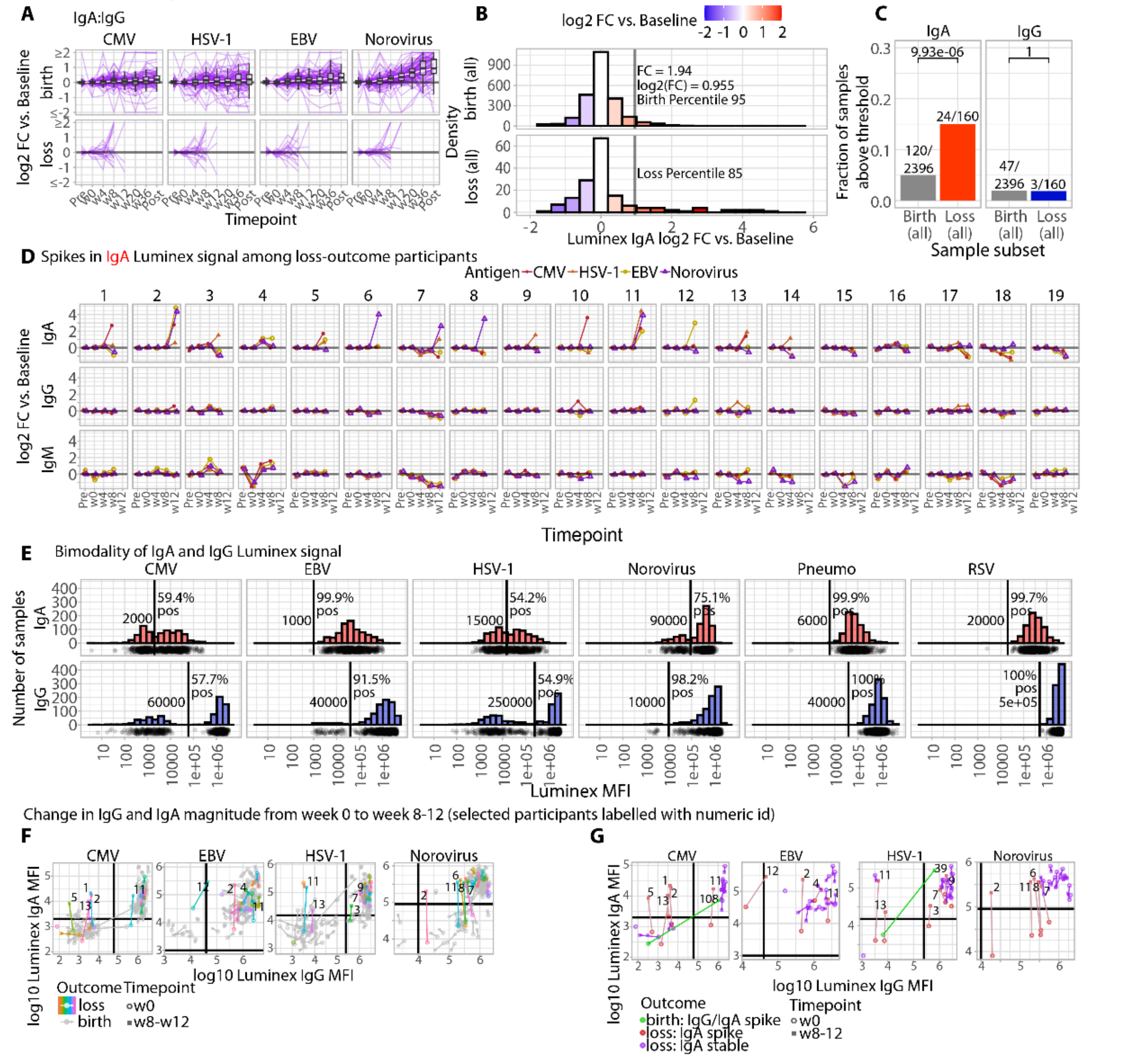

Fig. S5. IgA spikes precede miscarriage. Related to Figure 4.
(**A**) Ratio of IgA and IgG Luminex fold change relative to pre-pregnancy baseline for birth and loss outcome pregnancies for specified viruses. (**B**) Distribution of IgA fold-change vs. pre-pregnancy baseline for selected viruses of interest (CMV, HSV-1, EBV, and norovirus) for birth-outcome pregnancies at the week 4, week 8, week 12, week 20, week 36, and postpartum time points (top) and loss outcome pregnancies at the week 4, week 8, and week 12 time points (bottom). Vertical line indicates 95th percentile score for birth outcome (FC = 1.94). (**C**) Fraction of birth and loss samples from (B) scoring above FC of 1.94 for IgA (left) and IgG (right). Brackets are labeled with FDR values from Fisher's exact test. (**D**) Per-person fold change in Luminex antiviral titers for each loss outcome pregnancy for specified viruses for IgA, IgG, and IgM. Points are jittered horizontally slightly for improved visibility of overlapping data. (**E**) Distribution of Luminex MFI for IgA (red) and IgG (blue) for selected viruses. Vertical lines indicate threshold used for calling seropositivity with percentage of samples scoring as seropositive specified. (**F-G**) Scatterplot of Luminex IgG MFI on the x axis and IgA MFI on the y axis with lines connecting week 0 and week 12 samples per person for (F) birth outcome samples in grey and loss outcome samples in color. For pregnancies that terminated prior to week 12, the week 8 sample was used instead. (G) for loss outcome samples for which an IgA spike is observed in red, loss outcome samples for which an IgA spike is not observed in purple, and selected birth outcome samples with suspected true infection in green.

Supplementary Figure S6

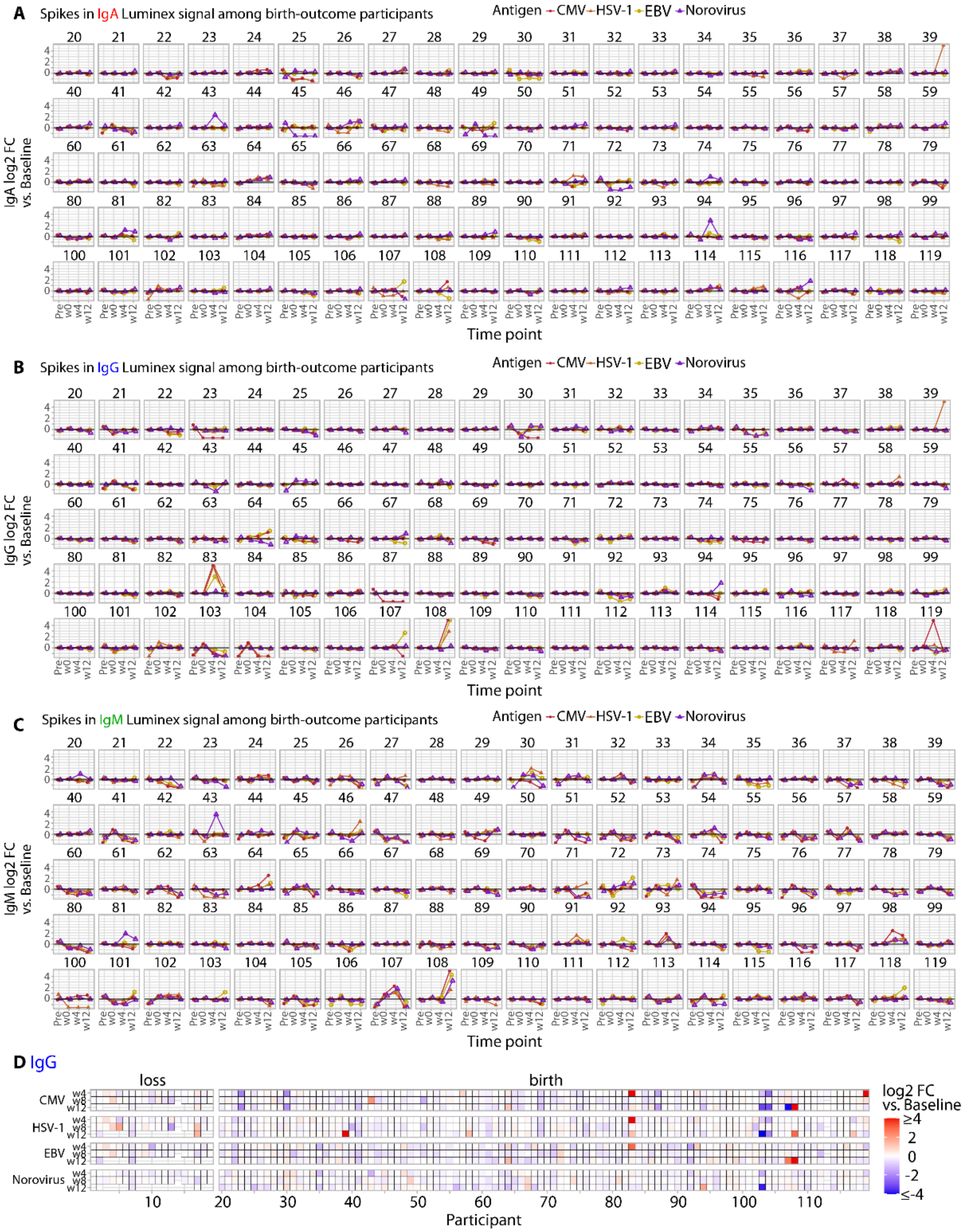

Fig. S6. Multi-isotype antiviral Luminex fold change. Related to Figure 4.
(**A-C**) Per-person fold change in Luminex antiviral titers for each birth outcome pregnancy for CMV, HSV-1, EBV, and norovirus for (A) IgA (B) IgG and (C) IgM. **(D)** Heatmap splitting by pregnancy outcome showing individual log2 fold change compared to pre-pregnancy baseline for Luminex antibody binding signal for selected viruses of interest for IgG.

Supplementary Figure S7

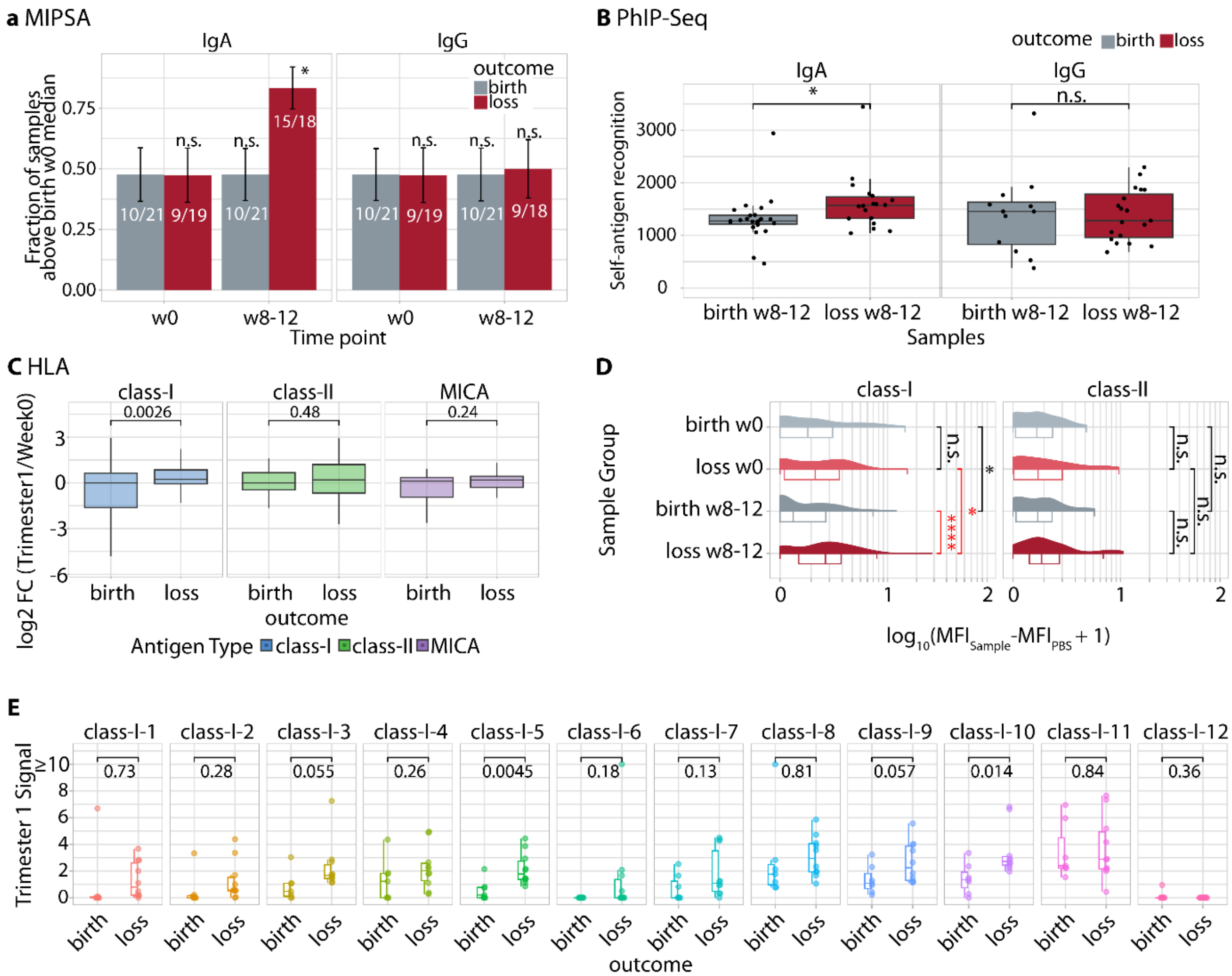

Fig. S7. Enhanced self-antigen recognition detected preceding miscarriage. Related to Figures 4 and 5. (**A**) Bar plots showing the fraction of samples with MIPSA self-antigen recognition higher than the median week 0 MIPSA self-antigen recognition for that isotype. Error bars show the standard deviation from 1000 bootstrapped samplings of each subgroup. Asterisk indicates FDR < 0.05 for a Fisher's exact test comparing the number of samples above and below the median score for birth outcome week 0 and the subgroup of interest. **(B)** Comparison of the number of peptides that score as enriched from PhIP-Seq for IgA (left) and IgG (right) using a human peptidome library for samples from the last time point collected prior to miscarriage or samples from birth outcome pregnancies, matched for gestational age. Asterisks indicate FDR < 0.05 for a Wilcoxon test comparing self-antigen recognition scores from birth and loss outcome pregnancies. (**C)** Box plots showing the distribution of IgA LabScreen Mixed scores as a ratio of each participant's trimester 1 score and their pre-pregnancy score. For each participant, there are 12 points for class I, 5 for class II, and 2 for MHC Class I-Related Chain A (MICA), corresponding to the number of distinct bead readouts included in the assay. Statistics indicate nominal p-values from a Wilcoxon test comparing the distributions of ratios for birth and loss-outcome pregnancies. **(D)** Distributions of background-subtracted scores across all class I (repeated from Fig. 5 for comparison) and class II antigens and samples, stratified by antigen class, pregnancy outcome, and time point. Significance indicators indicate the results of Wilcoxon tests comparing each group of scores (p ****<0.0001, *<0.1, n.s. not significant). Red brackets with asterisks indicate an increase in anti-HLA signal, either over time or associated with miscarriage, while the black bracket and asterisk indicate a reduction in anti-HLA signal over time in the birth cohort. **(E)** Birth and loss outcome LabScreen for each of the 12 separate class I read outs. Brackets are labeled with nominal p-values from a Wilcoxon test comparing birth and loss outcome scores.

Supplementary Figure S8

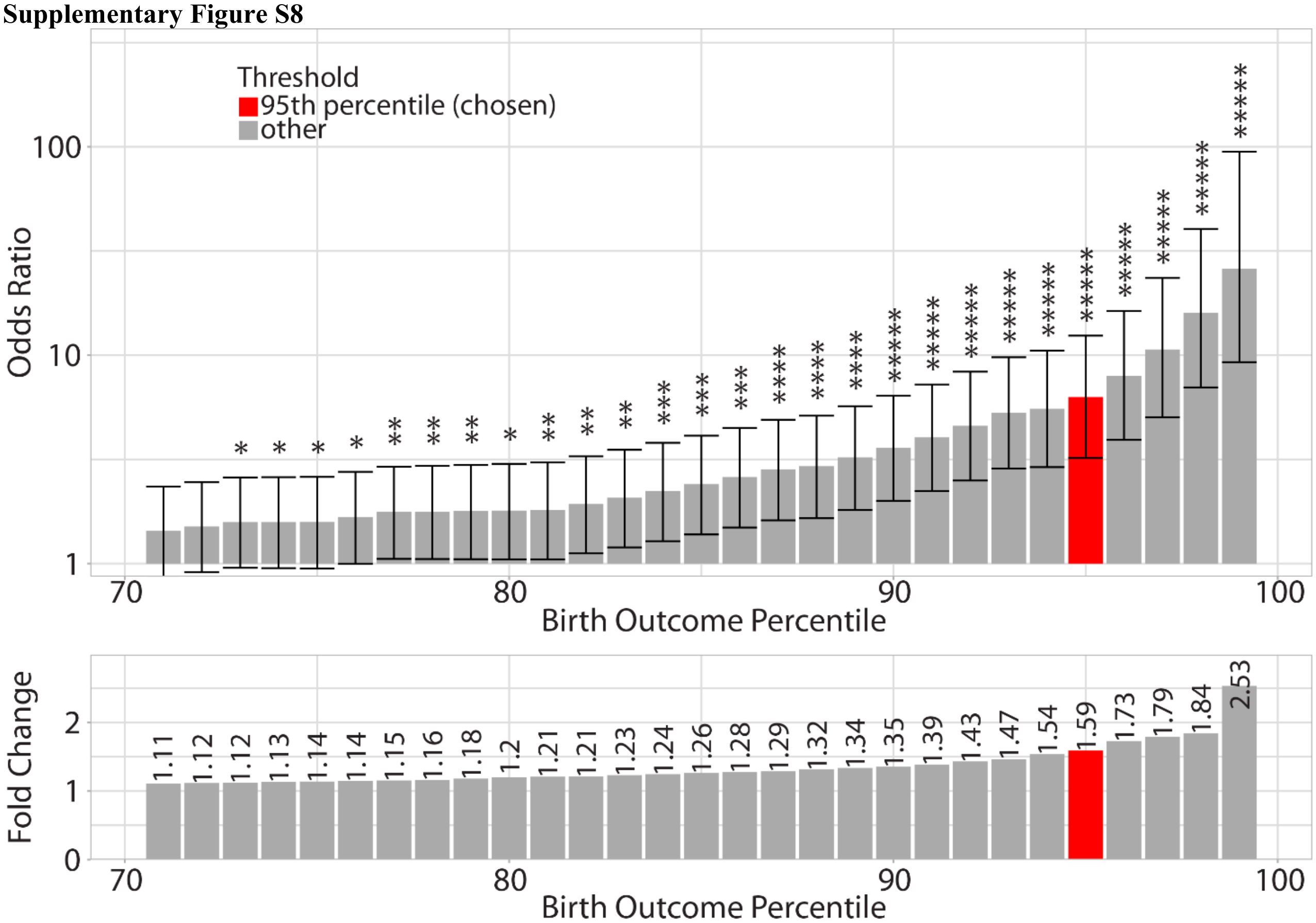

Figure S8. **IgA STORM is robustly associated with miscarriage across a wide range of thresholds. Related to Figure 4**. Thresholds for IgA fold-change ranging from fold increase of 11% (71st percentile of week 12 birth outcome scores) up to a fold increase of 153% (99th percentile of week 12 birth outcome scores) were evaluated for discrimination of pregnancy outcome (birth, miscarriage). On top, odds ratios are calculated, with y axis values on a log scale. Error bars indicate the 95% confidence interval for odds ratio. Asterisks indicate the significance level emerging from a Fisher's exact test for each threshold (* $p < 0.1$, ** $p < 0.05$, *** $p < 0.01$, **** $p < 0.001$, ***** $p < 0.0001$). Threshold chosen in the study of 95th percentile of birth outcome scores is highlighted in red.

Supplementary Figure S9

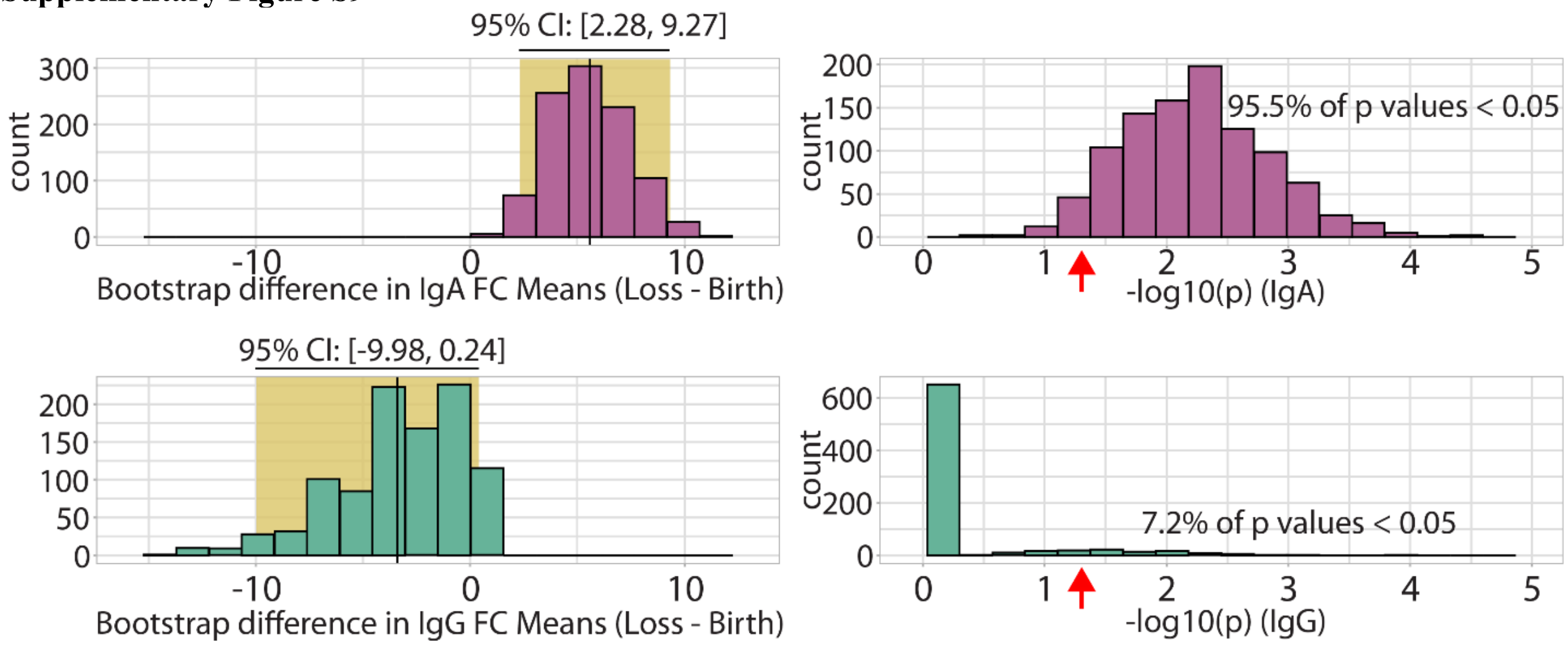

Figure S9. Bootstrapping evaluation of IgA STORM. Related to Figure 4. For IgA (top, pink) and IgG (bottom, teal), random sampling with replacement was performed 1,000 times. In each sampling iteration, for each isotype, paired data from week 0 and the week 12 time point was considered (or week 8 if week 12 sample was not collected/available). Across the four STORM antigens (CMV, EBV, HSV-1, and norovirus), the maximum fold change was calculated for each participant. Then, the mean maximum fold change was calculated for sampled participants, stratified by pregnancy outcome (miscarriage or live birth). The difference in mean fold change between pregnancies that end in miscarriage and pregnancies that end in live birth was computed for each iteration, and the resultant distribution is plotted in the two plots on the left. Text above the plot and the yellow rectangular area indicate the 95% confidence interval defined by these distributions. The vertical bars on these two left-side plots indicate the difference in means observed in the study. On the right, for each iteration, a t-test was performed to compare the distribution of fold change scores observed in pregnancies that ended in miscarriage and those that ended in live birth. The distribution of these p-values is plotted, and the red arrow below the x-axis indicates a p-value of 0.05 for reference.

Supplementary Figure S10

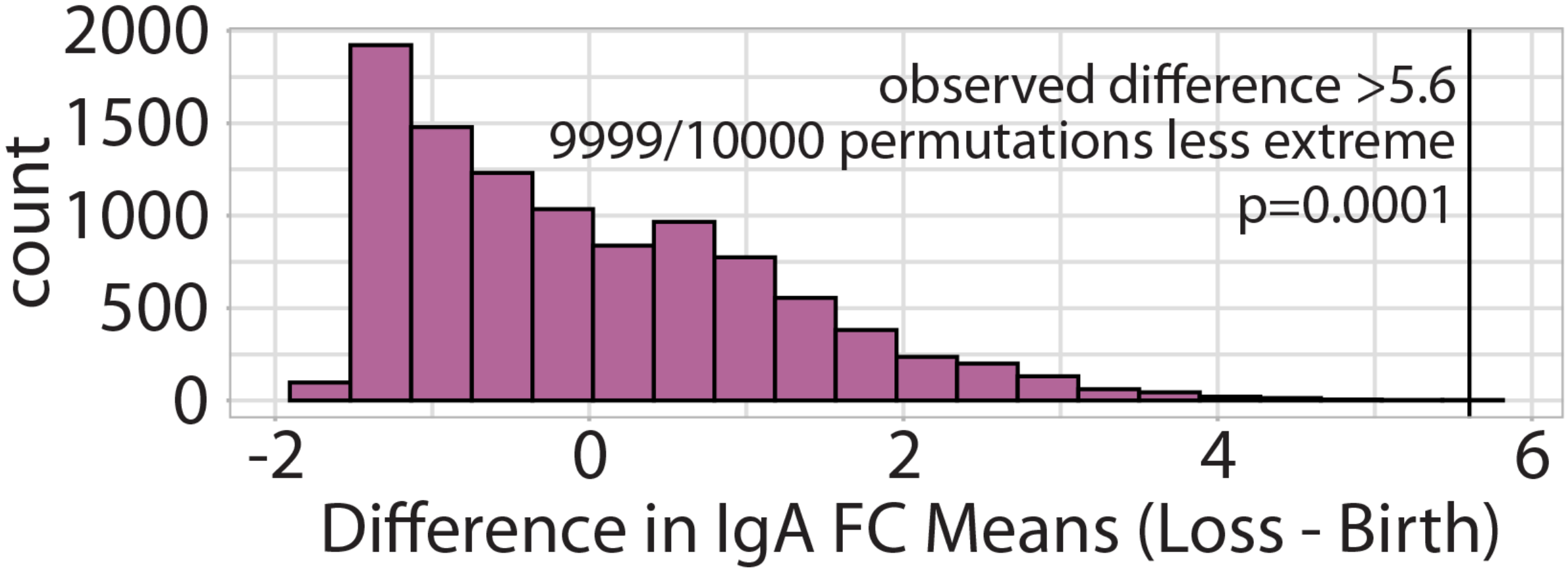

Figure S10. Permutation test evaluation of the significance of IgA STORM. Related to Figure 4. Paired IgA data was considered from week 0 and week 12 (or week 8 if week 12 sample was not collected/available). For all 119 participants, the maximum fold change was calculated across the four STORM antigens (CMV, EBV, HSV-1, and norovirus). Then, repeated for 10,000 iterations, metadata labels of whether each participant's pregnancy ended in live birth or miscarriage were randomized and recorded. From each randomized set of metadata labels, the mean STORM FC score was calculated for pregnancies labeled as ending in miscarriage and those labeled as ending in live birth. The histogram represents the distribution of difference in fold change means obtained from these randomly permuted metadata. The vertical line indicates the difference in means actually observed in the study (5.6), which correspond to a permutation p-value of 0.0001.

Supplementary Figure S11

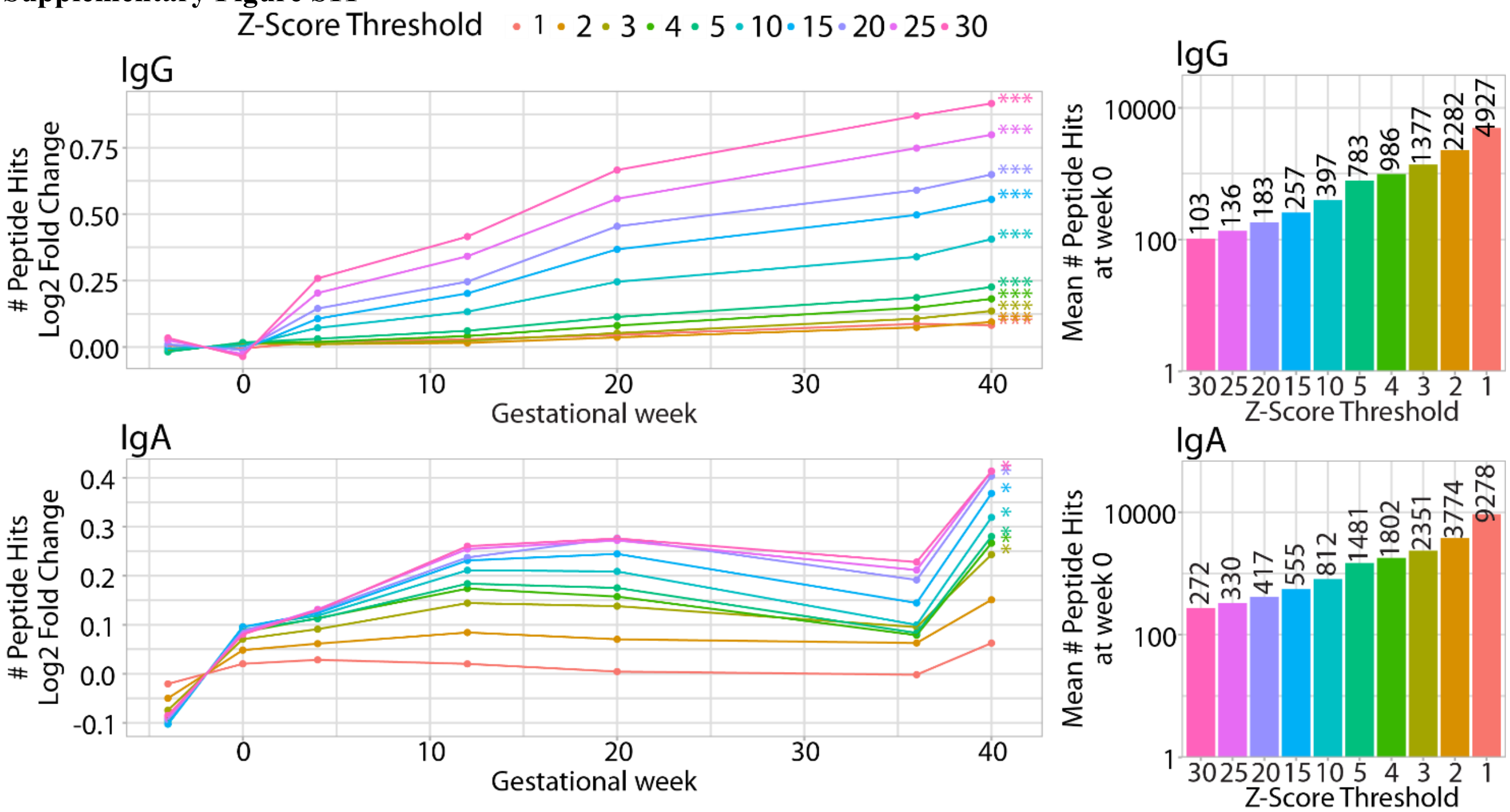

Figure S11.Antibody breadth increase is observed across a broad range of z-score thresholds. Related to Figure 2. (left) Change in antibody breadth over the observed gestational time course at different z-score thresholds for IgG on top and IgA below. Z-score threshold legend is at the top. The post-partum time point is coded as week 40. Asterisks to the right indicate significance of a Wilcoxon test testing the null hypothesis that the median fold change in antibody breadth at the post-partum time point is equal to 0 (log2 FC equal to 1) (*<0.05, ***<0.001). (right) bar plots indicating the average number of peptide hits at week 0 for each Z-score threshold for IgG on top and IgA below.

Supplementary Figure S12

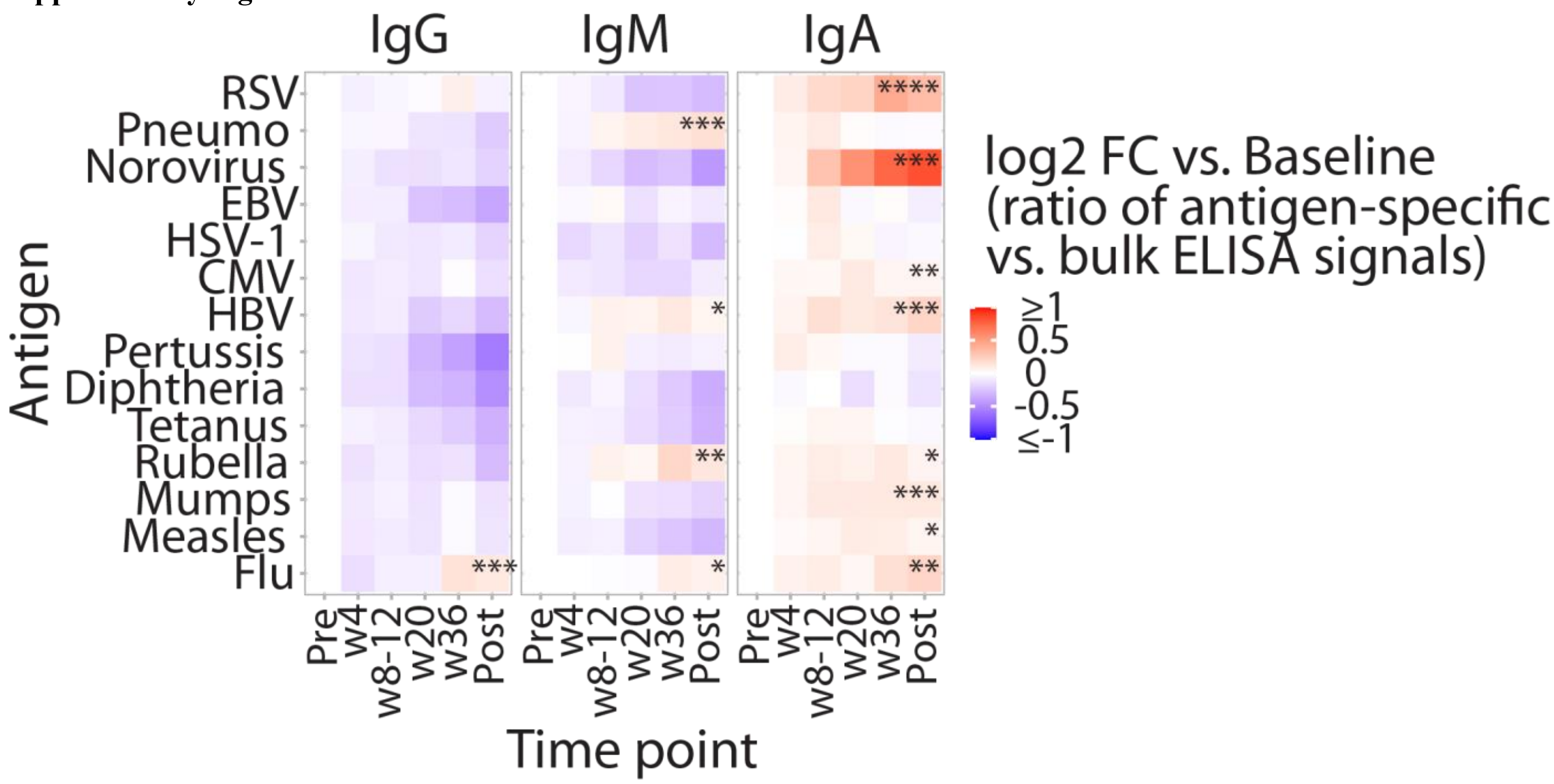

Figure S12. Median Luminex fold change vs. pre-pregnancy baseline, normalized to bulk isotype concentration by ELISA. Related to Figure 3. Asterisks indicate the BH-corrected significance level for one-sided t-test for the null hypothesis that post-partum fold-change in antigen-specific signal normalized to total isotype signal is less than or equal to 1 (*<0.05, **<0.01, ***<0.001, ****<0.0001).

Supplementary Figure S13

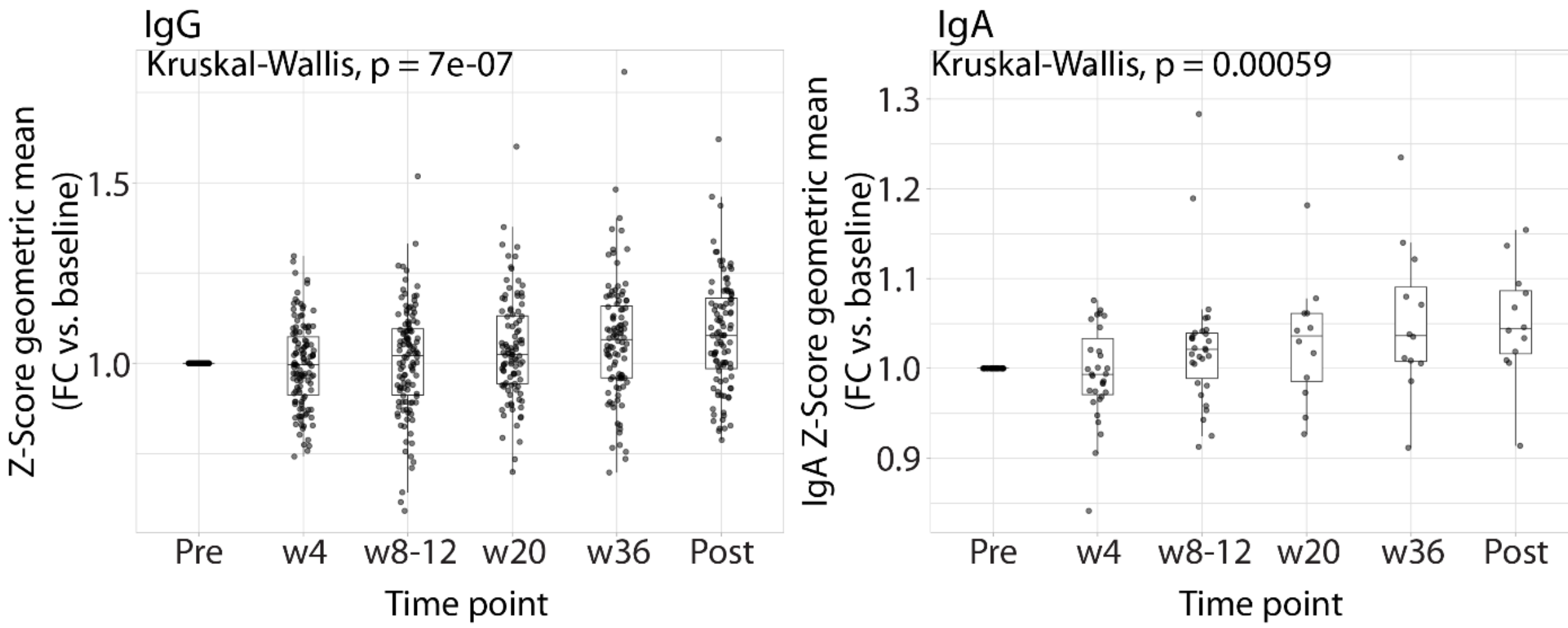

Figure S13. **VirScan hit magnitude increases over gestational time. Related to Figure 2**. Boxplots overlayed with horizontally jittered scatterplots representing the distribution of the geometric mean of Z-score magnitudes across the cohort and over gestational time for IgG (left) and IgA (right). For each isotype, text on top indicates the p-value from a Kruskal-Wallis test testing that the distributions at all time points are drawn from the same underlying distribution.

Supplementary Figure S14

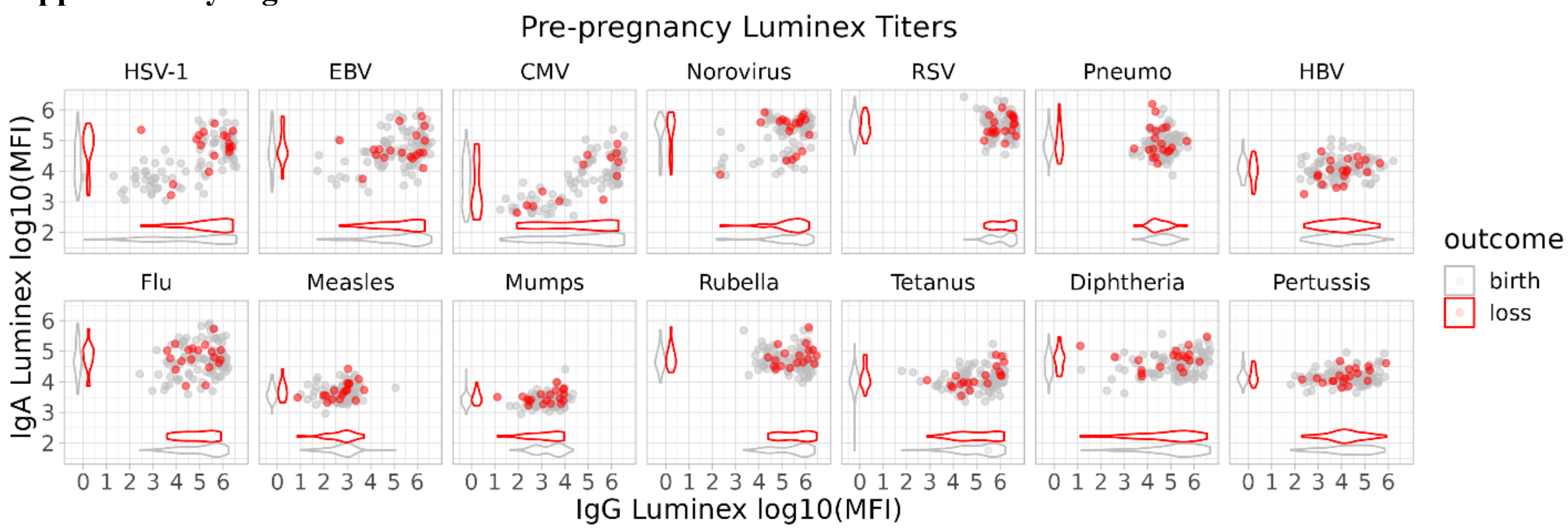

Figure S14. Distribution of pre-pregnancy raw Luminex MFI is not significantly different in pregnancies that end in miscarriage and those that end in live birth. Related to Figure 4. Raw Luminex MFIs are plotter for IgG (x) and IgA (y) for birth-outcome pregnancies (grey) and miscarriage-outcome pregnancies (red). Violin plots to the left and bottom of each scatterplot indicate the distribution of scores by pregnancy outcome for IgA (left) and IgG (bottom). Data is separated for each of the 14 antigens tested by Luminex. None of the antigens showed a statistically significant difference between pregnancy outcomes by t-test.

Table S4. Commercial antigens used for Luminex assay. Related to Figures 3 and 4.

Commercial protein antigens purchased for Luminex serological profiling. The first three columns indicate the supplier name, the product name, and the product catalog number when available. Items with the same label in the "Pool" column were mixed together prior to coupling to Luminex beads.

**Vendor**	**Item**	**Catalog #**	**Pool**
List Biological Laboratories	Pertussis toxin (50ug)	180	
Native Antigen (Vendor: Cedarlane)	Tetanus Toxoid, Recombinant Heavy Chain Fragment C (100ug)	REC31801-100	
Immune Tech	EBV gp350/220 (100ug)	IT-005-035p	
Immune Tech	HA1 (A/New Caledonia/20/1999) (100ug)	IT-003-001p	A
Immunetech	HA1(B/Phuket/3073/2013)	IT-003-B11p	A
Immunetech	HA1(H1N1)(A/Michigan/45/2015)	IT-003-00105p	A
BioRad Antibodies	Measles - Endmonston	PIP013	
ATCC	Pneumococcal Polysaccharide Powder Type 19A	301-X	B
ATCC	Pneumococcal Polysaccharide Powder Type 7F	108-X	B
Cedarlane	Diphtheria toxin	DIP-TNL	
BioRad Antibodies	Mumps - Enders	PIP014	
Vendor: Cedarlane	Norovirus VP1 GII.4	REC31620-100	
BioRad Antibodies	Rubella HPV-77	PIP044	
Immune Tech	HSV-1 gD(Ectodomain)	IT-005-055p	
Aviva systems biology	HBV HBsAg Adw	OPPA00881	
Sino Biological	Human cytomegalovirus (HCMV) Glycoprotein B / gB Protein (His Tag)	10202-V08H1	
NIH	RSV A/B Pre F Fusion Protein		C
NIH	RSV A/B Post F Fusion Protein		C
