# Supplementary material for: Humoral pregnancy analyses reveal strategies for fetal protection and aberrancy in miscarriage
Source: Cell Rep Med. 2026 Aug 19;7(9):102993. doi: 10.1016/j.xcrm.2026.102993 (PMC13589508; doi:10.1016/j.xcrm.2026.102993)
Supplement: Supplementary file 2 [file mmc14.pdf]

Cell Reports
Medicine

Article

Humoral pregnancy analyses reveal strategies for fetal protection and aberrancy in miscarriage

Graphical abstract

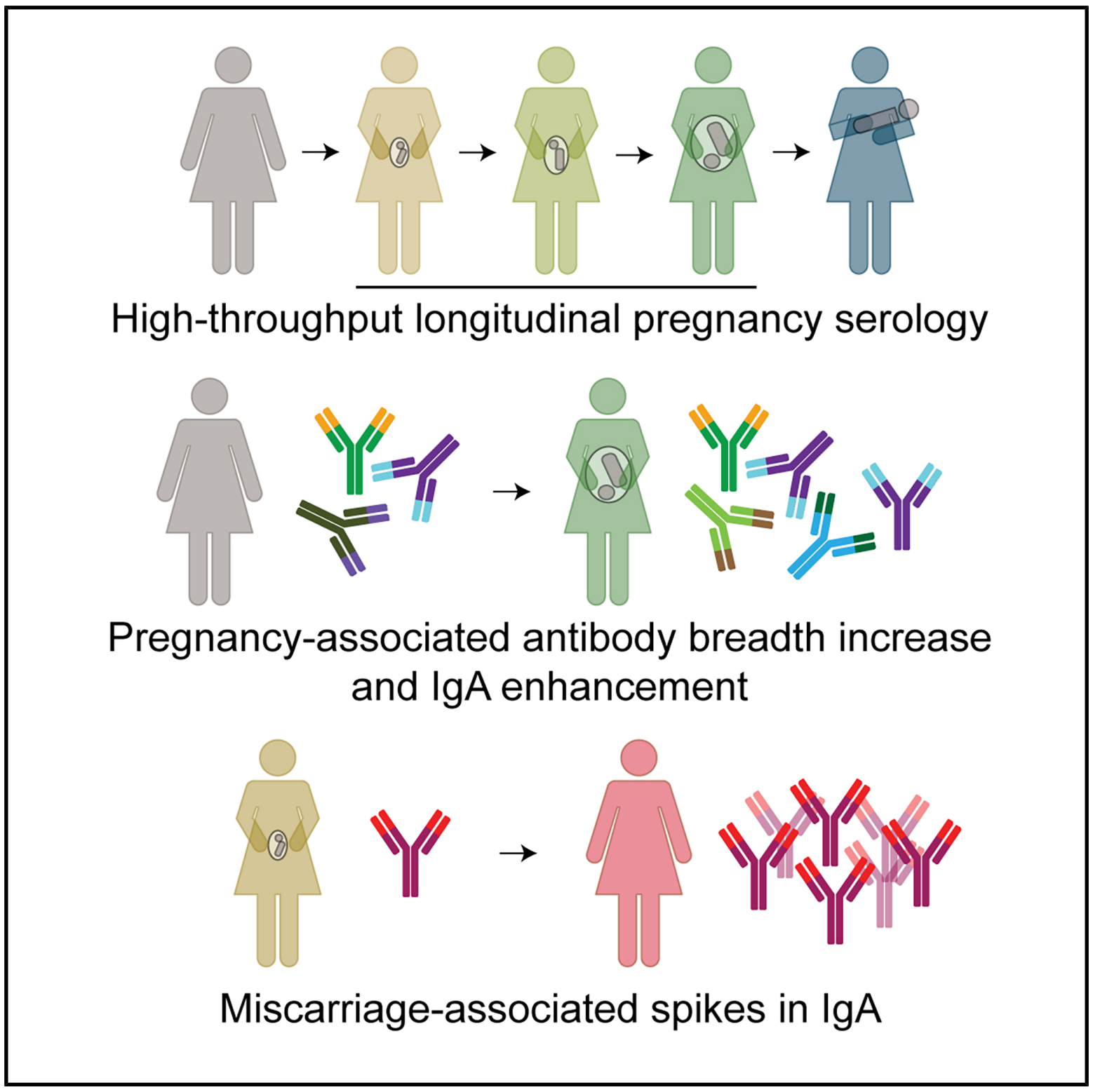

Highlights

- Longitudinal serology of 119 pregnancies reveals maternal antibody dynamics
- Antibody repertoire diversity increases during pregnancy for many pathogen classes
- Levels of antiviral serum IgA increase relative to matched antiviral IgG or IgM
- Increases in IgA for cross-reactive antigens and HLA are linked to miscarriage

Authors

Brandon M. Sie, Yumei Leng, Nadege Nziza, ..., Boris Julg, Galit Alter, Stephen J. Elledge

Correspondence

selledge@genetics.med.harvard.edu

In brief

Sie et al. performed high-throughput, longitudinal serology during human pregnancy, revealing maternal immunological dynamics. They found enhancement of maternal humoral immunity over pregnancy, both in antibody diversity and in relative concentrations of IgA. Furthermore, they identified spikes in IgA antigen signals preceding miscarriage, targeting both cross-reactive antigens and HLA.

Sie et al., 2026, Cell Reports Medicine 7, 102993
September 15, 2026 © 2026 The Author(s). Published by Elsevier Inc.
https://doi.org/10.1016/j.xcrm.2026.102993

CellPress

Cell Reports Medicine

CellPress
OPEN ACCESS

Article

Humoral pregnancy analyses reveal strategies for fetal protection and aberrancy in miscarriage

Brandon M. Sie,[1,2] Yumei Leng,[1,2] Nadege Nziza,[3] Moriah M. Mitchell,[1,2] Nicholas E. Webb,[3] Yunjoo Lee,[3] Sunni L. Mumford,[4,5] Enrique F. Schisterman,[5] H. Benjamin Larman,[6] Boris Julg,[3] Galit Alter,[3] and Stephen J. Elledge[1,2,7,*]

[1]Division of Genetics, Department of Medicine, Howard Hughes Medical Institute, Brigham and Women's Hospital, Boston, MA 02115, USA

[2]Department of Genetics, Harvard Medical School, Boston, MA 02115, USA

[3]Ragon Institute of Mass General, MIT, and Harvard, Cambridge, MA 02139, USA

[4]Epidemiology Branch, Division of Intramural Population Health Research, Eunice Kennedy Shriver National Institute of Child Health and Human Development, National Institutes of Health, Bethesda, MD 20892, USA

[5]Departments of Biostatistics, Epidemiology, and Informatics, and Obstetrics and Gynecology Perelman School of Medicine, Blockley Hall, 423 Guardian Drive, Philadelphia, PA 19104-6021, USA

[6]Department of Pathology, Johns Hopkins University School of Medicine, Baltimore, MD 21205, USA

[7]Lead contact

*Correspondence: selledge@genetics.med.harvard.edu

https://doi.org/10.1016/j.xcrm.2026.102993

SUMMARY

The maternal immune system must simultaneously defend against pathogens with tolerance of the antigenically distinct fetus. Despite the central importance of immune modulation during pregnancy, epitope-level analyses of the maternal antibody repertoire remain lacking. Here, we performed high-throughput, multi-isotype longitudinal serology of 119 women from preconception through pregnancy, combining phage display of linear peptides with whole protein-based antigen assays to profile antibody responses to viral and self-antigens. We observed a broad expansion of the maternal IgG repertoire and a relative increase in IgA titers, both tracking with pregnancy progression. These changes may augment transplacental and mucosal immunity in the fetus and newborn, respectively. Notably, pregnancies ending in miscarriage exhibited pronounced IgA titer spikes against both viral and self-antigens unrelated to active infection (sensitivity 14/19, 74%, specificity 95%), indicating an underlying immune perturbation may precede pregnancy loss.

INTRODUCTION

During pregnancy, expectant mothers' immune systems must balance generation of effective immune protection for both mother and offspring and prevention complications that might arise from an immune response that targets the fetus. Notably, the developing immune system relies on maternal antibodies to confer passive protection against common pathogens.[1–7] To this end, IgG is transferred through the placenta prior to birth, and both IgG and IgA are transferred through breast milk.[3–5,8,9]

Pregnancy has generally been reported to be associated with a decline in maternal antibody titers over gestational age,[10–12] which occurs concurrently with an increase in maternal blood volume,[13] transfer of maternal antibodies across the placenta to the fetus,[3,5,6,14] and a rise in estrogen and progesterone levels.[15,16] Estrogen and progesterone affect the relative frequency of B cell subsets during pregnancy, resulting in a decline in naive B cells and an increase in plasma cells.[1,16] These changes in maternal B cells contributed to speculation that expectant mothers may experience changes in their susceptibility to infections or reduced response to vaccination.[3,17–19]

While analyses of immune changes during pregnancy have been performed,[1,20–25] a systematic understanding of the breadth and dynamics of gestational immunity at the epitope level is still lacking. Some recent studies have increased the resolution and throughput with which the depth of the maternal antibody repertoire can be profiled. However, these approaches have typically prioritized a relatively focused selection of antigens.[26,27] An enhanced understanding of the immune response during pregnancy could inform clinical decision-making, including responding to mid-gestational infections and optimizing the timing of vaccination.[28] Moreover, there is a need to understand potential differences between pregnancies that end in live birth versus miscarriage. Miscarriage affects 10%–25% of known pregnancies,[29,30] and approximately 28% of miscarriages occur after a previously experienced miscarriage.[31] Although chromosomal abnormalities and maternal age contribute to some miscarriage risk, it is estimated that 30%–50% of miscarriages are from unidentified causes.[32]

RESULTS

Longitudinal profiling of antiviral antibody specificity

To begin a system level analysis of the architecture of the humoral immune response during pregnancy at the epitope level, we employed longitudinal serum samples for 119 women who participated in the Effect of Aspirin in Gestation and Reproduction (EAGeR) trial[33–37] (Figure 1; Table S1). This trial evaluated

© 2026 The Author(s). Published by Elsevier Inc.
This is an open access article under the CC BY license (http://creativecommons.org/licenses/by/4.0/).

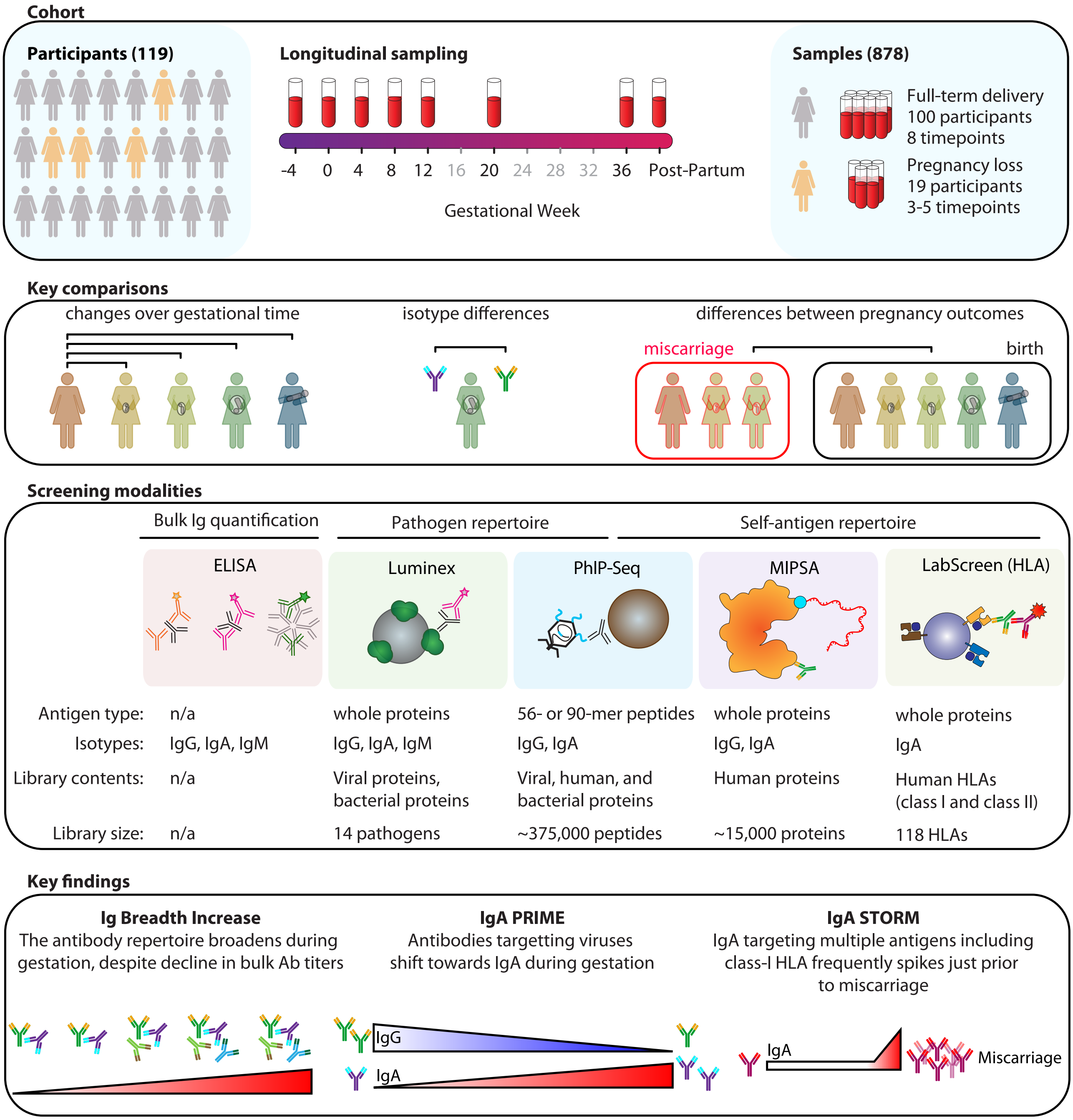

Figure 1. High-throughput, multimodal longitudinal profiling of antibody specificities during pregnancy
Overview of the current study. See also Figure S1 and Table S1.

effects of low-dose aspirin on pregnancy outcomes. Overall, our cohort contains 878 samples, representing 119 pregnancies, of which 100 resulted in full-term births (at gestational age ≥37 weeks), and 19 resulted in miscarriage between gestational weeks 8 and 15 (Figure 1; Figure S1A; Table S1, STAR Methods). Pregnancies not carried to term were followed through the last time point available, typically week 8 or 12, and was collected on average 9.5 ± 7.2 days prior to the estimated pregnancy loss. The birth- and loss-outcome cohorts do not statistically differ in maternal age, maternal BMI, prior pregnancies, prior pregnancy losses, or race (Figure S1A and Table S1). Gestational diabetes (1/119), preeclampsia (8/119), and allergic reaction (5/119) were only observed in the birth-outcome pregnancies. The 100 pregnancies described as "birth outcome" each resulted in a live birth, with typical birthweights (Figure S1A). All miscarriages during the study occurred during the first trimester.

None of the 119 pregnancies ended in preterm birth or stillbirth. Overall, ∼17% of pregnancies confirmed by ultrasound in the EAGeR trial ended in miscarriage,[33] in line with the estimated rate in a general US population of 10%–25%.[29,30,38]

Longitudinal antiviral antibody repertoires were examined using orthogonal high-throughput serology profiling technologies: high throughput immunoprecipitation and Luminex. Quantitative anti-viral antibody analyses were chosen because most individuals have previous immunity to hundreds of viral peptides whose analysis can provide great insight into the overall state of the humeral immune system. VirScan, a programmable phage display technology,[39] was used to systematically profile IgG and IgA antibody specificity using a library of 115,753 phage-displayed peptide epitopes representing ∼400 species and strains of human and several bacterial antigens from Immune Epitope Database (IEDB) (Figure 1; Tables S2 and S3). A custom Luminex assay was performed to profile antibody specificity against 14 common viruses and bacteria proteins, with separate scores for IgG, IgM, IgA, and for antibodies that bind to the neonatal Fc (fragment crystallizable) receptor (FcRn) (Figure 1; Tables S4 and S5). Total IgG, IgM, and IgA titers were also measured using ELISA.

We initially focused on viral epitopes, which are readily detected using VirScan.[39–42] Importantly, the humoral immune response to viruses is typical of other antigens,[43] thus patterns observed in the antiviral antibody repertoire are likely to reflect broader principles of immunity. We used two high-throughput assays to broadly profile potential self-antigens. Molecular indexing of proteins by self-assembly (MIPSA)[44] was used to profile IgG and IgA repertoires against a library of ∼15,000 human proteins. Additionally, phage immunoprecipitation sequencing (PhIP-seq) with a human peptidome[45,46] was performed as an orthogonal method using a peptide library of ∼260,000 peptides representing possible self-antigens. Finally, we performed an additional assay specifically designed to reveal antibody specificities for human leukocyte antigens (HLA) in an allele-specific manner.

Altogether, we profiled ∼146 million interactions between antibody repertoires and peptides by PhIP-seq and ∼2.5 million interactions between antibody repertoires and proteins by Luminex and MIPSA.

Quantification of antiviral antibody diversity by VirScan

Previous VirScan studies call viral seropositivity based on the number of peptides from a given virus that score as enriched and correlated clinical phenotypes to VirScan data.[39–42,47] This study explored the possibility of extending VirScan's utility beyond the characterization of specific viral infections. Here, we considered each person's antibody repertoire across the entire virome library as an indicator of their overall antibody-mediated immune function. We performed this analysis in the context of longitudinal pregnancy samples to examine whether this library-wide paradigm can reveal general insight into the immune response during pregnancy for pregnancies that end in live birth as well as miscarriage.

We quantified peptide enrichment[39–41] and verified proper representation of displayed peptides in our phage libraries (Figures S1B–S1E). We confirmed that the antibody repertoire measured by VirScan is stable throughout pregnancy (Figures S1F and S1G). The VirScan library has 56-mer linear peptides tiled every 28 amino acids, so most of the virome is directly represented by two peptides. Additionally, some proteins in the library share homology with others due to viral subfamily relationships and can be clustered into smaller groups of peptides that together represent distinct epitopes (Figure S2A). The number of distinct epitopes identified by VirScan for a given sample is referred to as VirScan "breadth". It represents the diversity of antiviral antibody specificity within a given sample. After collapsing enriched peptides for sequence similarity (Figure S2B), individuals in this cohort recognized a mean ± SD of 628 ± 191 IgG epitopes per sample (Figure S2C) and a mean ± SD of 651 ± 181 IgA epitopes per sample (Figure S2D), which is in the expected range for a typical healthy adult.[40,41]

Enhancement of epitope diversity occurs over gestational time

VirScan breadth is anticipated to decline over pregnancy due to the increase in blood volume, which dilutes antibody concentration.[13] We computed a fold-change (FC) in epitope breadth relative to the pre-pregnancy baseline (STAR Methods) for each non-baseline time point. The diversity of the maternal antiviral antibody repertoire is not detectibly changed during the first trimester but is significantly elevated in the second and third trimesters and at the post-partum time point for both IgG and IgA (Figures 2A and 2B). On average, IgG breadth increased by 15.6% (95% confidence interval [CI] 8.3%–23.0%), and IgA breadth increased by 19.6% (95% CI 2.6%–36.6%). VirScan enrichment magnitudes similarly increases over the course of pregnancy (Figure S13). This broadening of specificities occurs despite the expected decline in maternal antibody concentration over gestation, confirmed in this cohort for IgG, IgM, and IgA (Figure 2C; Figure S2G). Some individuals exhibit a decrease in breadth, possibly attributed to an active infection occurring near the start, which resolves with a subsequent decline and apparent drop in breadth at later time points. Nevertheless, the cohort overall shows an increase in VirScan breadth over the course of gestation.

To rule out an artifact due to increase in blood volume during pregnancy, we performed serial dilutions of serum input to VirScan (Figure 2D) which showed that dilution alone resulted in a reliable reduction of breadth. As antibody input into the assay decreases (ranging from 100% of our standard VirScan input to 12.5% of standard), the number of peptides that score as enriched per sample decreases following a logarithmic curve (Figure 2D). This is consistent with prior work.[40] Thus, the observed increase in maternal antibody repertoire diversity occurs despite declining maternal antibody concentration. Accounting for antibody concentration, IgG breadth increased by 22.4% (95% CI 13.7%–31.0%) (Figure 2E), and IgA breadth increased by 33% (95% CI 11.4%–55.4%). A chi-squared test also indicates a statistically significant change in antibody repertoire breadth over time (Figure 2F). A generalized estimating equations model with an autoregressive AR(1) working correlation structure corroborates significant positive association between gestational time and VirScan breadth, with an estimated

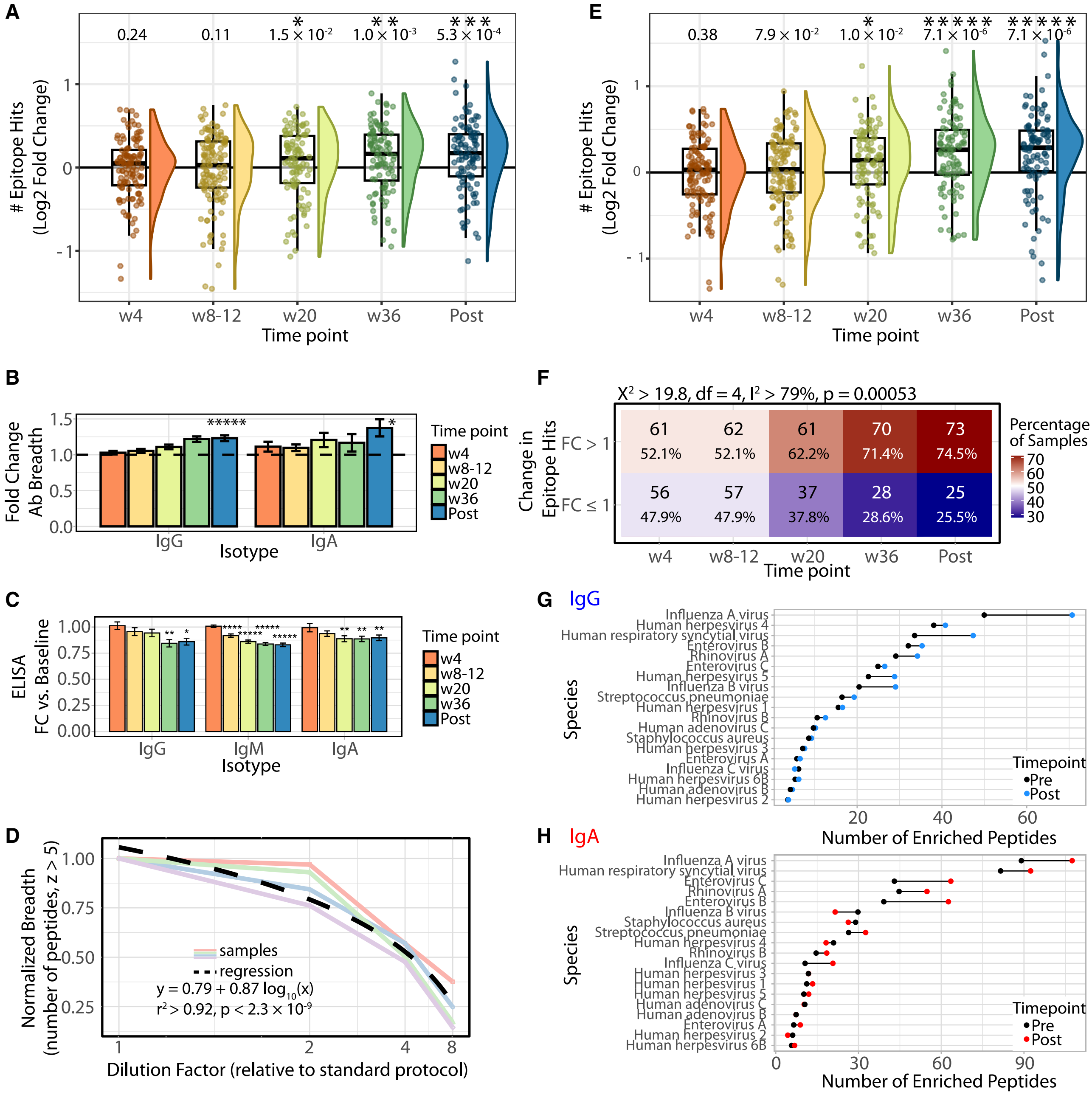

Figure 2. Antiviral antibody diversity broadens during pregnancy

(A and E) Boxplots for fold-change in IgG VirScan breadth. Significance values are FDR from a two-sided Wilcoxon rank-sum test comparing median of fold-change of samples at each time point to FC = 1. FDR *<0.05, **<0.01, ***<0.001, ****<0.0001. (A) Without antibody concentration correction (normalization) (E) after adjusting breadth for sample antibody concentration based on the curve generated in (D). Samples were screened in duplicate.

(B) Mean fold-change in normalized antibody breadth over pregnancy time by IgG VirScan and IgA VirScan. Error bars indicate standard error of the mean (SEM). FDR-corrected *p* values are shown from one sided *t* tests for the null hypothesis that postpartum breadth fold-change equals 1. FDR *<0.1, *****<0.00001.

(C) Bar plot of mean change in ELISA IgG, IgM, and IgA titers for the relative to pre-pregnancy baseline. Error bars indicate standard error of the mean (SEM). Asterisks indicate FDRs from one sided *t* tests for the null hypothesis that ELISA fold-change is greater than or equal to 1. FDR *****<0.00001, ****<0.0001, ***<0.001, **<0.01, *<0.05, unlabeled not significant.

(D) Effect of serum dilution on IgG VirScan breadth. The dashed curve shows a logarithmic fit to the data.

(F) Chi-squared table for samples with breadth fold-change greater than 1 versus less than or equal to 1.

(G and H) Number of non-collapsed enriched peptides for selected pathogens, before and after pregnancy for (G) IgG VirScan, (H) IgA. See also Figures S2, S11, and S13, Tables S2, S3, and S10.

CellPress
OPEN ACCESS

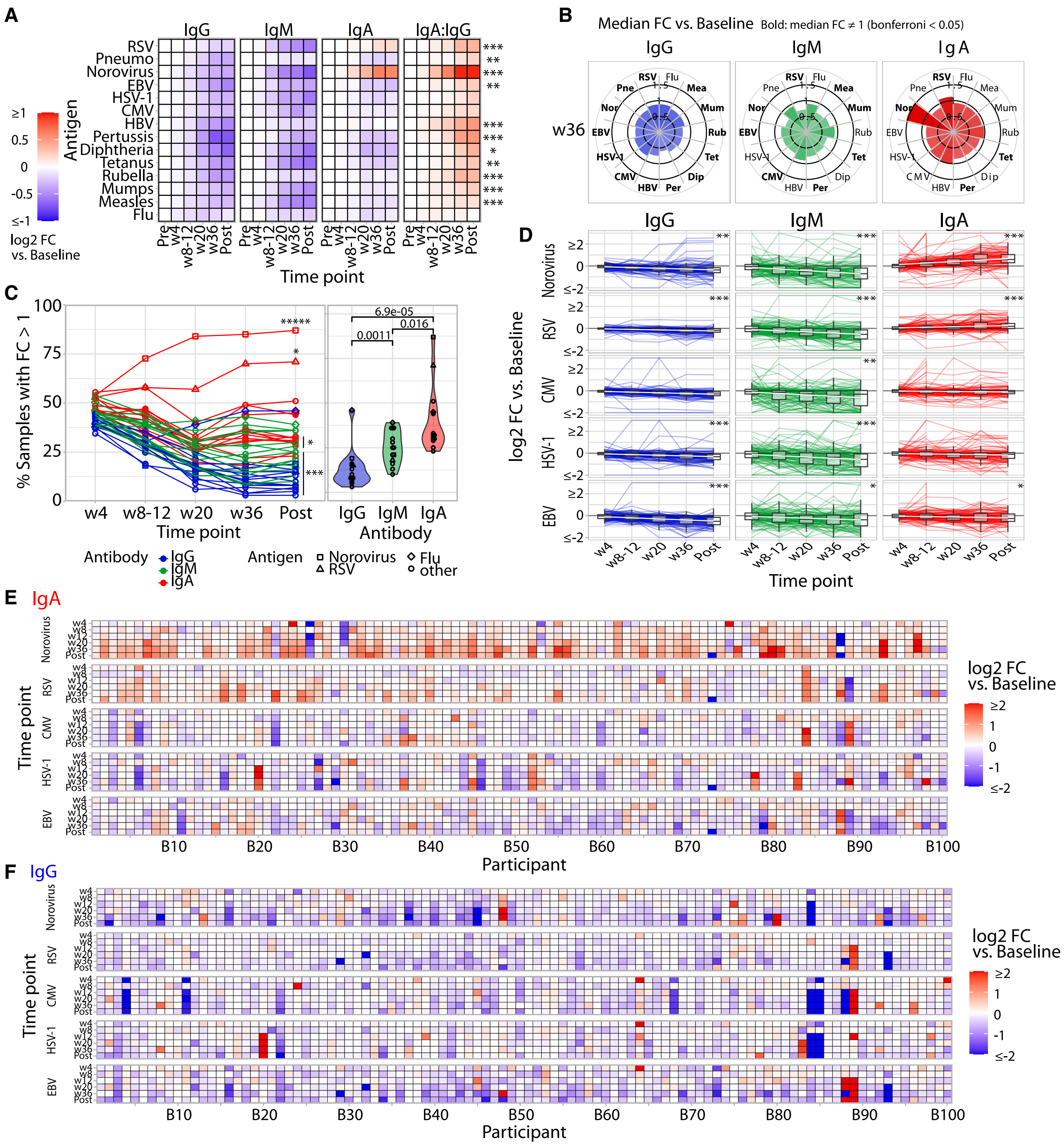

Figure 3. IgA-specific pregnancy-induced maternal enhancement

(A) Median fold-change in Luminex MFI compared to pre-pregnancy baseline. The fourth column shows the ratio of median fold-changes for IgA to IgG. Asterisks indicate Bonferroni-corrected *p* values for *t* tests for the null hypothesis that the log2 week 36 IgA:IgG fold-change ratio is equal to 0. ***<0.001, **<0.01, *<0.05.

(B) Flower plot showing median fold-change in Luminex MFI at week 36 compared to pre-pregnancy. Bolded virus abbreviations indicate that a two-sided Wilcoxon rank-sum test yielded a Bonferroni-corrected *p* value <0.05 for the null hypothesis that median FC = 1. Flu, influenza; Mea, measles; Mum, mumps; Rub, rubella; Tet, tetanus; Dip, diphtheria; Per, pertussis; HBV, Hepatitis B virus; CMV, cytomegalovirus; HSV, 1-herpes simplex virus 1; EBV, Epstein-Barr virus; Nor, norovirus; Pne, streptococcus pneumoniae.

(C) (Left) Sample fraction exhibiting fold-change greater than 1 for each Luminex antigen-antibody combination. Asterisks indicate FDR significance values for chi squared tests comparing the number of samples with FC > 1 versus FC ≤ 1. FDR *<0.1; ***<0.001; *****<0.00001. (Right) Violin plots show percentage increasing scores across all viruses assayed by Luminex.

(legend continued on next page)

0.59% increase in VirScan breadth per week compared to pre-pregnancy baseline ($\beta = 0.0059$, SE = 0.0010, Wald = 35, $p = 3.4 \times \cdot 10^{-9}$, $\alpha = 0.55$). We observed broadening of the number of enriched peptides across multiple pathogen classes in both IgG and IgA VirScan, especially among common viruses for which we detect antibody signal by VirScan prior to pregnancy (Figures 2G and 2H). Pregnancy outcome was not associated with differential change in VirScan breadth (Figures S2E and S2F) or different bulk antibody levels by ELISA (Figures S2G and S2H). Receipt of aspirin versus placebo in the EAGeR trial has no measurable effect on VirScan breadth (Figure S2I). Thus, despite decrease in antibody concentration during pregnancy,[10–12] and experimental confirmation that the VirScan breadth decreases as antibody concentration decreases, the observed diversity of the maternal antibody repertoire broadens over gestational age.

Identification of isotype-specific and virus-specific trends in antibody signals by Luminex

VirScan was particularly useful for assessing the overall breadth of the immune response through high-throughput linear epitope profiling. However, we expect most epitopes to be conformational. To complement VirScan and gain deeper insight into the humoral immune system, we employed an orthogonal Luminex-based assay. Luminex was chosen due to its high sensitivity, ability to capture both linear and conformational epitopes, and its ability to readout signals against multiple different antibody isotypes. By Luminex, we profiled antibody specificity against 14 full-length viral protein antigens, chosen for high prevalence and known compatibility with the assay (Figure 1; Tables S4 and S5), with separate readouts for IgG, IgM, IgA, and FcRn binding. Each antigen was conjugated to distinct Luminex beads, and detection antibodies were used to generate fluorescent signals corresponding to the binding of distinct antibody subclasses, as previously described.[5,26,48,49]

For each participant, two pre-pregnancy time points were averaged to establish a baseline, and fold-changes were calculated by comparing Luminex mean fluorescence intensity of each sample to this baseline. Overall, median IgG and IgM titers consistently decline over gestation, while median IgA titers remained relatively stable. Notably, increases in IgA levels were observed for anti-norovirus and anti-RSV (respiratory syncytial virus) antibodies (Figures 3A and 3B; Figure S3A). For several other tested viruses, IgA levels increased relative to IgG or IgM (Figure 3A; Figures S3B and S4B), suggesting that an overall increase in IgA levels outside of anti-norovirus and anti-RSV may be obscured by the dilution effect of maternal blood volume. Significant increases in antigen-specific IgA relative to bulk IgA were observed at the post-partum time point for rubella, RSV, norovirus, mumps, measles, Hepatitis B virus (HBV), flu, and cytomegalovirus (CMV) (Figure S12).

FcRn is important for the transfer of IgG across the placenta from mother to fetus, especially in the third trimester, and that the Fc region of IgG can be modified to encourage preferential transfer of particular IgG antibodies across the placenta.[3,50,51] Since FcRn binds to IgG[52] and measured maternal antiviral IgG signals tend to decline over gestational time, it is expected that maternal FcRn-binding signal also declines in maternal circulation over gestational time as we observed (Figure S3C).

We also visualized each individual's fold-change in Luminex signal (Figure 3D; Figures S3C and S3D; Figure S4). Despite variability, at the post-partum time point, 87% of participants had higher measured levels of anti-norovirus IgA than at their pre-pregnancy baseline, and 71% of participants had higher measured levels of anti-RSV IgA than at their pre-pregnancy baseline (Figures 3C–3E), in contrast to other measured antibody-antigen combinations. On average, anti-norovirus IgA titer increased by 62% in this cohort (95% CI 49.1%–78.7%) over the course of pregnancy. Increases in anti-norovirus IgA and anti-RSV IgA titers co-occurred with a decline in corresponding IgG titers (Figures 3E and 3F; Figures S3A and S3D).

To rule out explanations for observed IgA increases involving seasonality, we stratified the longitudinal trajectories by month of conception (Figure S3E). Increases in anti-norovirus IgA titer and anti-RSV IgA titer occur regardless of the month of conception and are, therefore, unlikely to be explained by seasonal waves of infection. Similarly, increases in IgA antiviral titers are not confined to a particular sample collection site and thus do not seem to be explained by a regional viral outbreak (Figure S3F) and are not measurably affected by receipt of aspirin vs. placebo in the original trial. Thus, these data indicate that IgA levels in pregnant women overall do not follow the general decline seen for IgG antibodies and, for some mucosal viruses such as norovirus and RSV, exhibit a phenomenon that we will refer to as IgA pregnancy induced maternal enhancement (IgA PRIME)—an augmented IgA response during pregnancy.

VirScan data also suggested increased IgA signal to norovirus and RSV over gestation (Figures S3G and S3H), which further supports this finding. Public epitope peptides from norovirus capsid protein VP1 and RSV surface glycoproteins, for which whole proteins were used as antigens in Luminex, exhibited increasing enrichment by VirScan over gestational time (Figures S3G and S3H). Thus, we find an IgA-specific increase in certain antiviral antibodies both using linear peptides whole proteins. Together, these data suggest a boosting of an IgA-specific, antigen-specific response to norovirus and RSV during pregnancy.

Clinically determined viral infection status was not available, so we cannot reference clinical infection data for active infection. However, data collected during the EAGeR trial of influenza vaccination aligns well with our serological profiling of anti-influenza antibodies. We observed a heterogeneous influenza response by Luminex (Figure S3I), which is partially explained by the fact that 21 of 119 participants received a flu vaccination during their pregnancy. The vaccinated individuals showed a

(D) Line plot showing participant Luminex fold-change compared to pre-pregnancy for selected viruses of interest. Boxplots show median, 25th and 75th percentiles, and whiskers spanning up to 1.5 inter-quartile range. Asterisks indicate Bonferroni-corrected *p* values for t-tests for the null hypothesis that the log2 week 36 fold-change is equal to 0. P_{corr} ***<0.001, **<0.01, *<0.05.

(E and F) Heatmap showing individual fold-change scores for each participant (column) over gestational time (rows) for IgA (E) and IgG (F). See also Figures S3, S4, and S12, Table S4.

week-36-versus-baseline fold-change in flu IgG Luminex signal with a mean of 2.74 (95% CI range 2.15–3.33) compared with 1.22 (95% CI range 1.12–1.31) for women who did not receive a documented flu vaccination during pregnancy. This difference is enhanced for FcRn-binding antibodies, for which the same fold-change has a mean of 18.52 (95% CI range 9.99–27.05) for women who received a flu vaccination during pregnancy and a mean of 1.66 (95% CI range 1.42–1.89) for women who did not (Figure S3I).

Differential patterns of antiviral antibody specificity in pregnancies that end in live birth versus miscarriage

In the RSV and norovirus IgA data, we observed abundant spikes in antibody levels early in some pregnancies (weeks 8–12) with no data at later time points (Figure 3C). We then identified that spikes were occurring in the last samples collected from pregnancies that would shortly thereafter result in miscarriage. We call this phenomenon IgA STORM (spikes tracking onset of repeat miscarriage). To explore further, individuals' trajectories in fold-change for antibodies recognizing different viruses were stratified by pregnancy outcome (Figures 4A and 4B). We assessed all antibody-antigen combinations, performed multiple hypothesis correction (STAR Methods), and then limited our subsequent analysis to a subset of pathogens: high-seroprevalence viruses for which there is no vaccine and that had a statistically significant false discovery rate (FDR): CMV, Epstein-Barr virus (EBV), herpes simplex virus 1 (HSV-1), and norovirus.

Several pregnancies ending in miscarriage exhibited a spike in fold-change IgA signal against CMV, HSV-1, EBV, and/or norovirus at the last available time point before the pregnancy ended (Figure 4A). This spike in IgA signal is not accompanied by a corresponding spike in IgG signal (Figure 4B), and therefore a spike in IgA signal tends to correspond to a spike in IgA:IgG ratio (Figure S5A). When comparing the last samples available from pregnancies that ended in miscarriage versus samples from pregnancies that ended in livebirth matched for gestational time, we find a miscarriage-associated, IgA-specific elevation in the maximum Luminex fold-change (Figure 4C) and in number of viruses with a detectible spike (Figure S4C) across these four viruses. These IgA spikes without a corresponding IgG spike are distinct from the expected pattern observed in likely viral infections observed in the birth-outcome, for which we observed concurrent spikes in both IgG and IgA targeting a particular antigen (Figure 4F; Figure S5G).

To visualize the co-occurrence of spike in IgA signal for multiple viruses in a single individual, we plotted heatmaps of isotype-specific fold-change for viruses of interest across gestational age (Figure 4E; Figure S6D), line plots for the CMV, HSV-1, EBV, and norovirus signals for the 19 pregnancies that ended in miscarriage (Figure 4D; Figure S5D), and equivalent line plots for the pregnancies that ended in live birth (Figure S6). Nine of 19 miscarriages exhibited an IgA FC > 4 (above the 99.75th percentile of week 12 birth-outcome IgA fold-change scores) for at least one of CMV, HSV-1, EBV, or norovirus, with some individuals exhibiting a high fold-change against multiple viruses at the same time point. Five additional miscarriages exhibited an IgA FC above the 95th percentile of equivalent scores from week 12 birth-outcome pregnancies (FC > 1.6) (Figure 4G).

Notably, some of the most pronounced IgA STORM events were detected in samples collected in the two days prior to miscarriage, often accompanied by concurrent IgA spikes against multiple viruses (Figure 4D, participant IDs 2 and 11). Among five participants who exhibited an IgA STORM spike against multiple viruses (participant IDs 2, 5, 7, 11, and 13), the mean interval between last sample collection and estimated date of miscarriage was 4.8 days (SD 4.6 days). In contrast, the remaining 14 participants, who underwent miscarriage but did not display multi-virus IgA STORM, had a substantially longer average interval of 16.7 days (ranging up to 40 days). Despite the small sample size ($n = 19$), this difference was statistically significant (two-sided t test $p = 0.023$). These findings suggest that IgA STORM may be most detectable within approximately one week of miscarriage, implying that our current data likely underestimate its overall prevalence. Notably, we detect IgA STORM at the last time point preceding miscarriage but not at the second-to-last time point (approximately 4 weeks in between), suggesting that IgA STORM may not be detectable four or more weeks prior to miscarriage. Nevertheless, spikes in recognition of proteins from different viruses seem to foreshadow an adverse outcome for almost three-quarters of the miscarriage-bound pregnancies in this cohort. Of the 14 individuals who experienced a miscarriage for whom an IgA spike was observed, only one (participant ID 12) had a concurrent IgG spike of similar magnitude (Figure S5D).

To confirm statistical enrichment of spikes in antiviral antibody titers preceding miscarriage, we compared the frequency of large increases in Luminex signals across pregnancy outcome. Combining data for CMV, HSV-1, EBV, and norovirus, we observed that the IgA fold-change data from the week 12 birth-outcome samples followed a normal distribution (Figure 4G, top). We defined a threshold for calling an antibody titer spike based on the 95th percentile of scores in the birth distribution, FC > 1.59. For the equivalent distribution of IgA fold-change from the samples just preceding miscarriage, we observed a disproportionate fraction of data exhibiting an IgA fold-change greater than the 95th percentile threshold of the birth-outcome. We then counted the number of samples that scored above or below the IgA FC > 1.59 threshold for week 12 birth-outcome samples and last time point miscarriage outcome samples, performed Fisher's exact test, and found a significant enrichment in IgA spikes in the miscarriage outcome compared to the birth-outcome (Figure 4H, left). We did not observe a statistically significant difference in IgG spikes between the two populations (Figure 4H, right). Performing the same IgA analysis with samples from all non-baseline time points (week 4 through postpartum) yielded a similarly significant IgA-specific enrichment in titer spikes among pregnancies that ended in miscarriage (Figures S5B and S5C). We further tested internal robustness of IgA STORM to distinguish pregnancy outcome by bootstrapping (Figure S9) and permutation testing (Figure S10) and observe significantly higher IgA fold-change preceding miscarriage compared to control pregnancies (95% confidence interval for difference in means [2.28, 9.27]) by bootstrapping, with our observed difference (+5.6 higher average fold-change preceding miscarriage) generating a permutation test p value of 0.0001. This significant difference was only

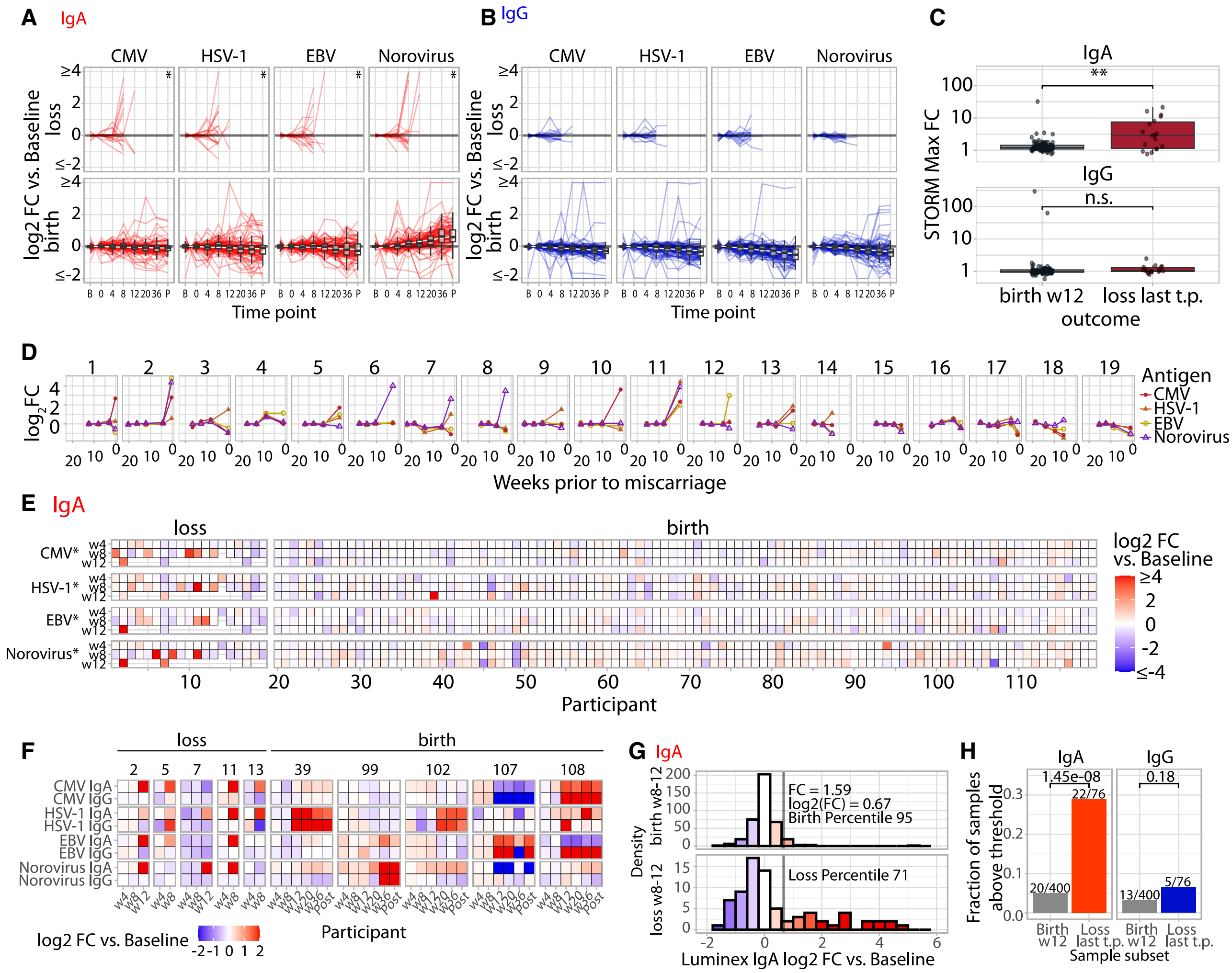

Figure 4. IgA spikes tracking onset of repeat miscarriage

(A and B) Line plot split by pregnancy outcome showing participant Luminex log2 fold-change compared to pre-pregnancy for selected viruses for (A) IgA and (B) IgG. X labels indicate time point. B, pre-pregnancy baseline, 0–36 gestational week; P, post-partum. Asterisks indicates that loss-outcome samples at weeks 8–12 were significantly enriched over birth-outcome samples for scores above FC > 2 by Fisher's exact test. For birth-outcome pregnancies, boxplots show median, 25th and 75th percentiles; whiskers spanning up to 1.5 inter-quartile range; white lines show linear regression fit.

(C) Boxplot comparing a simplified IgA STORM metric in pregnancies that ended in livebirth versus miscarriage for IgA (top) and IgG (bottom). For each pregnancy, the highest Luminex fold-change for CMV, HSV-1, EBV, or norovirus was recorded from the last sample prior to pregnancy loss, or from gestational time matched samples from pregnancies that ended in live birth. Significance values indicate the FDR from *t* tests comparing the score distribution for birth-outcome and loss-outcome samples: ** FDR<0.01, n.s. not significant.

(D) Line plots for each of 19 pregnancies that ended in miscarriage, showing Luminex IgA fold-change compared to pre-pregnancy for CMV, HSV-1, EBV, and norovirus. The *x* axis shows the weeks between sample collection and estimated pregnancy loss.

(E) Heatmap splitting by pregnancy outcome showing individual log2 fold-change compared to pre-pregnancy baseline for Luminex antibody binding signal for selected viruses of interest for IgA. Asterisks indicates that loss-outcome samples at weeks 8–12 were significantly enriched (FDR<0.1) over birth-outcome samples for scores above FC > 2 by Fisher's exact test.

(F) Selected participant data from (E) and Figures 3E and 3F. Data are displayed for loss-outcome participants with IgA signal increases against more than one of: CMV, HSV-1, EBV, and norovirus. Additionally, data are displayed for birth-outcome participants for whom we observe concurrent increases in IgA and IgG for a particular virus.

(G) Distribution of IgA fold-change vs. pre-pregnancy baseline for selected viruses of interest (CMV, HSV-1, EBV, and norovirus) for week 12 birth-outcome pregnancy samples (top) and last time point loss-outcome pregnancy samples (bottom). Vertical line indicates 95th percentile score for birth-outcome (FC = 1.59). The heatmap color scale is the same as used in (C)–(E).

(H) Fraction of birth and loss samples scoring above FC of 1.59, for IgA (left) and IgG (right). Brackets are labeled with FDR values from Fisher's exact test. See also Figures S4–S10 and S14; Table S4.

observed for IgA, not IgG. Receipt of aspirin versus placebo in the original trial is not associated with a significant difference in these IgA-specific spikes. Together, these results show that the magnitude of the IgA spikes that we observe preceding miscarriage are higher than can be explained simply by inter-individual variability.

The observed large spikes in fold-change are often associated with low pre-pregnancy levels. The distribution of antigen-specific antibody titers observed by our Luminex screening is often bimodal, and thus, we can draw a threshold line with which a seropositivity call can be made (Figure S5E). The miscarriage samples for which a large fold-change spike is observed often start with low magnitude Luminex MFI at pre-pregnancy, which increases to a level consistent with the "seropositive" mode of our score distributions (Figure S5F). However, in most birth-outcome samples, for these antigens, IgA-negative samples are also IgG-negative (Figure S5F) and IgA spikes tend to occur alongside a concurrent IgG spike (Figure S5G). By contrast, in individuals experiencing miscarriage, we observed spikes in IgA magnitude without corresponding spikes in IgG magnitude (Figure S5G). Additionally, the pre-pregnancy Luminex titers are not significantly lower for any of our tested viruses for the miscarriage-outcome pregnancies compared to the birth-outcome pregnancies (Figure S14), indicating that differences in the frequency of low basal levels of signals between the loss and birth cohorts are not responsible for differences in spike detection.

Regarding the possibility that IgA STORM represents polyreactive antibodies, we do not find evidence of a repertoire-wide bias toward epitopes with any particular amino acid composition. Since some of antigens are from viruses for which the patient has not been infected, some of the signal is likely due to cross-reactivity for IgA STORM. Regardless of the source, the observed signal remains likely to be meaningful given how strongly it diverges from the inter-individual variation observed in the birth-outcome cohort. Examining the distribution of IgA Luminex scores from pregnancies that ended in live birth, the 95th percentile of birth-outcome fold-change score at trimester 1 (relative to pre-pregnancy) is exceeded by 14 of 19 miscarriage-outcome pregnancies. This suggests a single metric indicator with approximately 74% sensitivity and 95% specificity within this cohort (95% confidence intervals: sensitivity 58.3%–90.0%; specificity 91.9%–98.4%). Statistical analyses further corroborate that the miscarriage outcome pregnancies are enriched for high IgA fold-change scores by Luminex (Figures S9 and S10). This trend matches the patterns we observed on follow-up with anti-HLA and broader anti-self-antigen screening, where high levels of IgA targeting human proteins were observed prior to miscarriage.

MIPSA and PhIP-seq reveals broader IgA self-antigen recognition preceding miscarriage

We examined the possibility that IgA STORM may correlate with a maternal immune response against human proteins by two orthogonal techniques. The first, MIPSA,[44] involves ~15,000 whole protein human antigens, and the second, PhIP-seq, involves the phage display peptidome approach of VirScan but with a ~260,000 peptide library tiling across human proteins. Based on the IgA-specific pattern preceding miscarriage that we originally observed by Luminex, we chose to specifically explore the hypothesis that pregnancies that end in miscarriage differ from pregnancies that end in livebirth matched for gestational age in their self-antigen repertoires. To this end, we included samples from two time points: week 0 and week 8–12 (typically the last time point available prior to miscarriage) and profiled both IgG and IgA repertoires by both methods.

By MIPSA, we detected a broader range of whole human proteins that are targeted at week 8–12 prior to miscarriage compared to those detected in pregnancies that result in a live birth for IgA, but not for IgG (Figure S7A and Table S6).

From PhIP-seq, we detect a larger set of human peptides that are bound by IgA antibodies from the samples from miscarriage-outcome pregnancies compared to those from birth-outcome pregnancies, without an equivalent difference for IgG (Figure S7B; Tables S7 and S8).

For both MIPSA and PhIP-seq, we checked for consistent enrichment of recurring targets across participants, which would suggest a pathway. While PhIP-seq and MIPSA have both been used to discover autoantigens that are corroborated by orthogonal studies,[44,45,53,54] such analyses involved enrichment of a consistent self-antigen across multiple representative of a disease class. In contrast, we observed a much more heterogeneous result in this pregnancy cohort, including for individuals who exhibit a spike in self-antigen breadth prior to miscarriage.

These two assays suggest that the IgA repertoires from samples just preceding miscarriage exhibited an increase in self-antigen recognition compared to gestational time-matched samples from pregnancies that ended in live birth.

Elevated levels of anti-HLA IgA antibodies detected prior to miscarriage

Of particular interest in a maternal immune response targeting human proteins are antibodies against HLA, which are associated with prior pregnancy[55,56] and miscarriage risk.[57,58] We adapted OneLambda LabScreen Mixed, a Luminex-based assay which detects anti-HLA IgG preceding organ transplantation (Figure 5A and Table S9), to detect anti-HLA IgA. We compared pregnancies ending in miscarriage with those ending in birth, matched for gestational age. To this end, we again included samples from two time points: week 0 and week 8–12 (typically the last time point available prior to miscarriage). We observed significantly higher IgA antibodies targeting class I HLA in pre-miscarriage samples from week 8–12 compared with pre-pregnancy that we did not detect in samples from birth-outcome pregnancies (Figure 5A; Figure S7C). Notably, while pregnancies that end in miscarriage exhibit a significant increase in their anti-class I HLA IgA over the first trimester of pregnancy, we observe a subtle but slightly significant decrease in anti-class I HLA IgA over the first trimester of pregnancy for birth-outcome pregnancies (Figure 5B). Additionally, significantly higher levels of IgA antibodies targeting class I HLA were found in pre-miscarriage samples from week 8–12 compared to birth-outcome samples from the same gestational age (Figures 5B and 5C; Figures S7C–S7E).

Presumably, an HLA-directed humoral response would involve antibodies targeting one particular HLA. We sought to

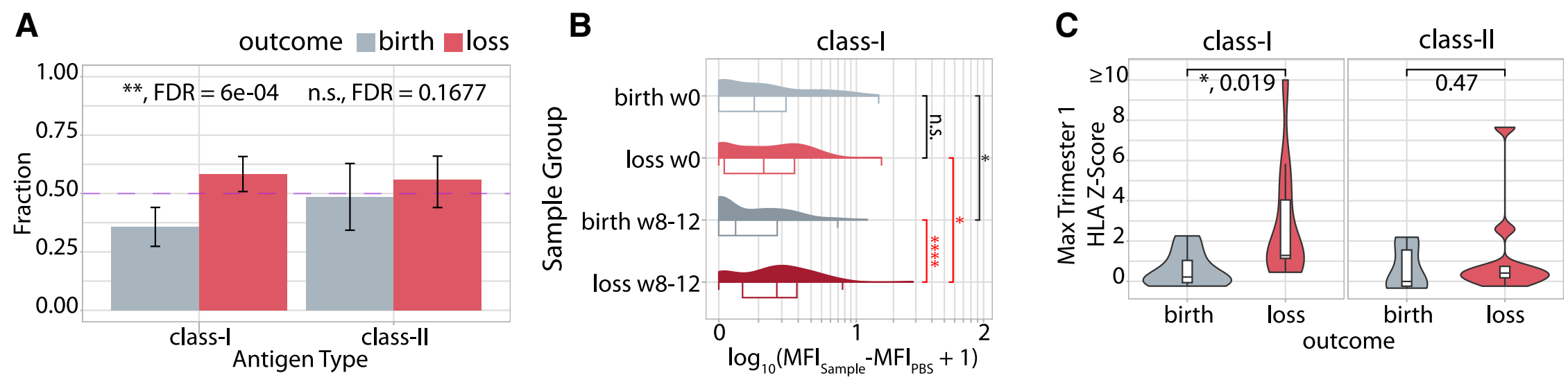

Figure 5. Higher levels of IgA targeting class I human leukocyte antigens detected preceding miscarriage

(A) Fraction of measurements with elevated reactivity at week 12 in birth and loss-outcome pregnancies, separated by antigen class. Error bars indicate bootstrap confidence intervals. FDRs correspond to the result of permutation tests comparing the observed difference fraction of elevated scores to the distribution of differences obtained by permuting sample labels (FDR **< 0.001).

(B) Distributions of background-subtracted scores across all tested class-1 HLAs and samples, stratified by pregnancy outcome and time point. Asterisks indicate significance of Wilcoxon tests comparing each group of scores (p ****<0.0001, *<0.1, n.s. not significant). Red brackets with asterisks indicate an increase in anti-HLA signal, either over time or associated with miscarriage, while the black bracket and asterisk indicate a reduction in anti-HLA signal over time in the birth cohort.

(C) Box and violin plots showing the maximum trimester 1 population-normalized class-I score for each tested participant. *p* value from a Wilcoxon test comparing the distribution of these scores for birth vs. loss-outcome (FDR *<0.05). See also Figure S7; Tables S6, S7. S8, S9, S11, S12, and S13.

overcome lack of HLA typing for this cohort by collapsing our anti-HLA antibody data to a single strongest antigen per person (STAR Methods). Comparing this measure across pregnancies that ended in miscarriage and livebirth, the samples preceding miscarriage exhibited significantly higher maximum anti-HLA IgA scores for class I, but not class II (Figure 5C).

DISCUSSION

Pregnancy represents a profound alteration of physiological homeostasis. Broad hormonal changes set in motion a series of adaptations to support the growth and development of the fetus in an environment that protects both the fetus and the mother-to-be. Changes to maternal immunity are critical because the mother's immune system must be tamed to enable tolerance of the fetus, but, at the same time, protect the fetus from infection *in utero* and after birth. In this study, we report a high-throughput, longitudinal, multi-isotype analysis of the antiviral antibody repertoire in 119 pregnant women to understand the antibody response at high resolution using two orthogonal technologies: PhIP-seq and Luminex.

The first major finding from this study involves antibody breadth during pregnancy. Maternal antibody concentrations are known to decrease during pregnancy,[10,16] which has been hypothesized to decrease the likelihood of a deleterious immune response against the fetus.[18] Unexpectedly, we observed that epitope-level antiviral IgG and IgA diversity increases over gestational age. These observations support the notion that maternal B cell immunity is not wholly suppressed during pregnancy, but rather the mother may be employing a deeper recall of immune memory, potentially enhancing protection to the mother and fetus. The magnitude of the antiviral antibody breadth increase observed here is 22% for IgG and 33% for IgA when accounting for change in bulk antibody concentration. The precedence for a change of this magnitude being meaningful comes from the observation that a 20% reduction in the breadth of diversity as a consequence of measles infection may increase the risk of disease from subsequent pathogen exposure.[40] Accordingly, increasing antibody breadth would be expected to increase protection. Pregnancy causes an intricate cascade of immunological changes, particularly within the T cell compartment.[59] After implantation, immunity transitions from a Th1 immunity to Th2 dominance, a change that reverses post-partum.[60,61] A higher Th2:Th1 ratio favors B cell activation,[62] consistent with the broader antibody repertoire observed by VirScan.

The second major finding involves the phenomenon that we preliminarily refer to as IgA PRIME. Unlike IgG and IgM levels that decrease during gestation, Luminex and VirScan both showed IgA levels for antigens increased. Although the most striking increases were observed for IgA targeting mucosal pathogens, norovirus and RSV, we also observed relative increases in IgA for many viruses when compared with the bulk IgA level or with antigen-specific IgG or IgM. There are several biological changes during pregnancy that may be involved. As previously mentioned, pregnancy is associated with a shift toward Th2-mediated immunity,[60] with an environment rich in TGF-β and IL-5 that facilitates class switch recombination to produce IgA.[63] Furthermore, conversion of IgA memory cells into plasma cells, facilitated by other Th2-associated cytokines, notably IL-6 and IL-10,[64] could play a role. Similarly, shifts in B cell populations toward greater preference for IgA could be driven by pregnancy-associated changes in the microbiota and hormone levels.[65,66] B cells express high levels of estrogen receptor and respond to the elevated levels of estrogen and progesterone characteristic of pregnancy. Monteiro et al. reported that high estrogen and progesterone levels favor expansion of particular follicular helper T (T_{FH}) subsets, as well as memory B cells and plasma cells,[16] findings consistent with our observation of a more robust maternal immune response during pregnancy, as in the increasing antibody breadth and IgA PRIME signals we observe. The possibility that IgA PRIME could be explained by subclinical viral exposures cannot be excluded, although the absence of parallel antigen-specific serum IgG reduces this possibility. We speculate that IgA PRIME could be part of maternal

preparation or "priming" for breastfeeding, which is primarily IgA transfer.[8,9,67,68] Notably, we still see an increase in overall VirScan breadth even if norovirus and RSV are excluded, emphasizing that IgA PRIME and antibody breadth increase are distinct phenomena.

The third major observation concerns distinct antibody repertoire patterns between full-term pregnancies and miscarriages, specifically a phenomenon we preliminarily refer to as IgA STORM. Careful analysis of IgA antibody dynamics revealed sharp spikes in IgA levels recognizing certain viral proteins for many women immediately prior to miscarriage, often to an extent well above that observed at any stage of healthy pregnancies resulting in live birth. Although we initially observed IgA STORM spikes in IgA levels in viral Luminex data, we also found that pre-miscarriage samples in this cohort show significantly higher levels of IgA against HLA class I and overall self-antigens, compared to controls from pregnancies that resulted in birth. Prior pregnancy and miscarriage are both associated with the presence of detectable antibodies against HLA.[55–58] These previous studies provide precedent for the concept of a pregnancy termination mechanism involving maternal antibodies recognizing paternal HLA. However, previous associations involved IgG rather than IgA. This work does not establish spikes in IgA as a cause of adverse pregnancy outcomes; rather, it reports a potential signal of immune dysregulation that will require future validation. Although the biological mechanism and physiological significance of IgA STORM remain unclear, its prevalence is striking. Given the multifactorial, heterogeneous nature of miscarriage, a preliminary biomarker that tracks with nearly 75% sensitivity (specificity 95%), even as an internal performance estimate, is of considerable interest for potential follow-up.[69]

One possible explanation for IgA STORM is viral induction of antigen-specific antibody responses that contribute to miscarriage. Viral infection has long been linked to miscarriage[70–73] and CMV is associated with adverse outcomes such as stillbirth,[74,75] although definitive evidence remains limited for many viruses.[31,76,77] However, infection alone is unlikely to explain these miscarriages for several reasons. First, IgG recognition of the same proteins is not elevated, as would be expected in a typical viral response. Viral infection in the context of impaired IgG production, while possible, is unlikely. For instance, some SARS-CoV-2 infections elicit IgA responses without detectable IgG.[78–81] Yet, one IgA STORM case showed robust IgG and IgA responses to flu vaccination, comparable to those in live-birth pregnancies, arguing against a general IgG deficiency. Second, IgA STORM often shows spikes against multiple pathogens per individual, with five of the ten strongest cases targeting two or more viruses. This pattern is inconsistent with typical infections and not observed in full-term pregnancies, where IgA responses to a single virus are usually accompanied by IgG. Together, these observations suggest that IgA STORM is unlikely to result from viral infection. Instead, the IgA reactivity likely reflects cross-reactivity, potentially as part of an aberrant anti-fetal immune response, since many targeted viral antigens show no evidence of actual infection.

A second possible explanation is that the IgA spikes could represent a dysregulated acceleration of pregnancy stage. IgA PRIME and IgA STORM both include IgA-specific effects that may result from aforementioned conditions favorable for IgA class-switching during pregnancy. However, the magnitude of fold-change spikes that we observe is much larger in IgA STORM than in IgA PRIME, even accounting for differences in timing, such that we believe that these phenomena arise from distinct mechanisms.

A third explanation involves an adverse immune response targeting the fetus. Pregnancy is a known risk factor for immunological dysregulation,[81,82] including in antiphospholipid syndrome, systemic lupus erythematosus, and rheumatoid arthritis (RA), all of which can complicate pregnancy. Additionally, IgA autoantibodies have been linked to tissue damage. IgA can recruit neutrophils via FcαR1 (CD89) on myeloid cells and has been implicated in diseases, including linear IgA bullous disease (LABD), dermatitis herpetiformis (DH), and RA.[83] When bound to neutrophils or macrophages, IgA can trigger phagocytosis or cytotoxic responses.[84,85] Moreover, IgA and IL-39 have been associated with neutrophil NETosis,[86,87] which is known to cause deleterious systemic inflammation in preeclampsia.[88] Additional pathogenic immune responses may similarly contribute to miscarriage in this cohort. Prior studies of recurrent miscarriage suggest that individuals with a history of prior miscarriage may harbor antibodies against preexisting fetal microchimeric cells, which can contribute to adverse outcomes in subsequent pregnancies.[89] All participants in the EAGeR cohort had experienced 1 to 2 prior miscarriages, which could prime maladaptive immune responses in latent pregnancies. Of the many possible explanations for IgA STORM considered, we believe that our data best support the hypothesis of a maternal anti-fetal immune response.

Limitations of the study

Although this study offers epitope-level resolution of maternal B cell immunity, the sample size (n = 119) limits statistical power, particularly for subgroup analyses. A larger cohort would enhance the robustness of these findings. Secondly, this study is also limited by the lack of parental and fetal HLA typing. Incorporating comprehensive HLA genotyping in future serological work would help clarify the relationship between anti-HLA IgA and miscarriage risk and allow determination of whether fetal HLA antigens might be targeted. Finally, this study cannot determine causality between the observed IgA STORM and miscarriage. Mechanistic questions remain open about whether elevated IgA responses precede and contribute to pregnancy loss, or whether immunological events triggered by impending miscarriage elicit the IgA response. It remains to be seen whether the mucosal IgA correlates with the presumably monomeric serum IgA detected in this study. Moreover, prior pregnancy losses could also prime the maternal immune system, influencing IgA responses in subsequent pregnancies.

Altogether, this study demonstrates that pregnancy is associated with dynamic remodeling of the maternal humoral immune system, likely to support fetal success, and that disruptions in this process may contribute to adverse outcomes. One key mechanism involves an expansion of antibody breadth, likely reflecting a deeper representation of the mother's antigen exposure history and potentially enhancing protection for both mother and fetus. Additionally, IgA PRIME may represent an

upregulation of IgA production for post-natal transfer to the infant. Together, breadth expansion and IgA PRIME illustrate how gestational immunity reshapes the maternal antibody repertoire, potentially optimizing offspring protection. In contrast, IgA STORM, an abnormal induction of cross-reactive IgA in some women prior to miscarriage, may offer an early clue into the etiology of subsets of pregnancy loss. It is marked by elevated IgA against class I HLA and a broader range of IgA targeting human proteins. Whether IgA STORM is a response to an underlying pathology or a causal factor remains unresolved. Clarifying this will require analyses of the IgA antibodies induced prior to miscarriage to determine if they are detrimental to pregnancy. If causally linked, these findings could inform the development of targeted interventions to prevent immune-mediated pregnancy loss. The antibody repertoire changes observed here likely reflect upstream dynamics, and continued investigation of the humoral immune landscape during pregnancy holds promise for deepening our understanding of these complex and finely tuned immunological adaptations.

RESOURCE AVAILABILITY

Lead contact

Requests for further information and resources should be directed to and will be fulfilled by the lead contact, Stephen J. Elledge (selledge@genetics.med.harvard.edu).

Materials availability

Any reagents that are unique to this study will be made available upon request with a completed materials transfer agreement.

Data and code availability

- Original antibody repertoire profiling data were deposited to Gene Expression Omnibus as GEO: GSE314505. They are publicly available as of the date of the publication. The DOIs are listed in the key resources table under "Deposited Data" as "Antibody repertoire profiling data" (GEO). Processed PhIP-seq, Luminex, MIPSA, and HLA data, along with library metadata, are available in supplementary tables.
- This paper does not report original code.
- Any additional information required to reanalyze the data reported in this paper is available from the lead contact upon request.

ACKNOWLEDGMENTS

Portions of this research were conducted on the O2 High Performance Compute Cluster, supported by the Research Computing Group, at Harvard Medical School. See https://it.hms.harvard.edu/our-services/research-computing for more information. We thank Michael Mina for helping access the cohort samples and Lindsey Sjaarda (NIH) and Elizabeth DeVilbiss (NIH) for information on the cohorts.

This study was supported by the following sources: the Howard Hughes Medical Institute (S.J.E.). Biospecimens and associated data were provided by the EAGeR trial, an initiative developed through intramural funding from the Eunice Kennedy Shriver National Institute of Child Health and Human Development, and supported by contract no. HHSN267200,603423, HHSN267200,603424, HHSN267200603426, HHSN275200403394C, HHSN275201100002I, and HHSN2750000I.

AUTHOR CONTRIBUTIONS

Conceptualization, S.J.E., G.A., and B.M.S.; data curation, B.M.S., N.N., Y.L., and N.E.W.; formal analysis, B.M.S.; funding acquisition, S.J.E. and G.A.; investigation, B.M.S., Y.L., N.N., M.M.M., N.E.W., and Y.L., resources, S.J.E. and G.A., supervision, S.J.E., G.A., B.J., and H.B.L.; visualization, B.M.S.; writing – original draft, B.M.S. and S.J.E.; writing – review and editing, B.M.S., S.J.E., G.A., E.F.S., S.L.M., and H.B.L.

DECLARATION OF INTERESTS

S.J.E. is a founder of and hold equity in Tscan Therapeutics, MAZE Therapeutics, Infinity Bio, Inc., and Mirimus, serves on the scientific advisory boards of Infinity Bio, Inc., and T-Scan Therapeutics, none of which affect this work. H.B.L. is a founder and SAB member of Infinity Bio, Inc. S.J.E. and H.B.L. have a patent on the VirScan technology. G.A. is an employee of AstraZeneca.

STAR★METHODS

Detailed methods are provided in the online version of this paper and include the following:

- KEY RESOURCES TABLE
- EXPERIMENTAL MODEL AND STUDY PARTICIPANT DETAILS
  - Human samples
- METHOD DETAILS
  - Phage immunoprecipitation and sequencing
  - Luminex
  - Profiling antibodies against HLA
  - MIPSA
  - ELISA
- QUANTIFICATION AND STATISTICAL ANALYSIS
  - Data analysis
  - Multiple hypothesis correction for IgA STORM
  - Collapsing LabScreen HLA data to a single strongest score
  - Z-scores
  - Covariates
  - Asterisks

SUPPLEMENTAL INFORMATION

Supplemental information can be found online at https://doi.org/10.1016/j.xcrm.2026.102993.

Received: December 4, 2025
Revised: April 19, 2026
Accepted: July 22, 2026
Published: August 19, 2026

REFERENCES

1. Abu-Raya, B., Michalski, C., Sadarangani, M., and Lavoie, P.M. (2020). Maternal Immunological Adaptation During Normal Pregnancy. Front. Immunol. *11*, 575197. https://doi.org/10.3389/fimmu.2020.575197.
2. Orije, M.R.P., Maertens, K., Corbière, V., Wanlapakorn, N., Van Damme, P., Leuridan, E., and Mascart, F. (2020). The effect of maternal antibodies on the cellular immune response after infant vaccination: A review. Vaccine *38*, 20–28. https://doi.org/10.1016/j.vaccine.2019.10.025.
3. Calvert, A., and Jones, C.E. (2017). Placental transfer of antibody and its relationship to vaccination in pregnancy. Curr. Opin. Infect. Dis. *30*, 268–273. https://doi.org/10.1097/QCO.0000000000000372.
4. Dolatshahi, S., Butler, A.L., Pou, C., Henckel, E., Bernhardsson, A.K., Gustafsson, A., Bohlin, K., Shin, S.A., Lauffenburger, D.A., Brodin, P., and Alter, G. (2022). Selective transfer of maternal antibodies in preterm and fullterm children. Sci. Rep. *12*, 14937. https://doi.org/10.1038/s41598-022-18973-4.
5. Jennewein, M.F., Goldfarb, I., Dolatshahi, S., Cosgrove, C., Noelette, F.J., Krykbaeva, M., Das, J., Sarkar, A., Gorman, M.J., Fischinger, S., et al. (2019). Fc Glycan-Mediated Regulation of Placental Antibody Transfer. Cell *178*, 202–215.e14. https://doi.org/10.1016/j.cell.2019.05.044.

6. Palmeira, P., Quinello, C., Silveira-Lessa, A.L., Zago, C.A., and Carneiro-Sampaio, M. (2012). IgG placental transfer in healthy and pathological pregnancies. Clin. Dev. Immunol. *2012*, 985646. https://doi.org/10.1155/2012/985646.

7. Glezen, W.P. (2003). Effect of maternal antibodies on the infant immune response. Vaccine *21*, 3389–3392. https://doi.org/10.1016/s0264-410x(03)00339-6.

8. Atyeo, C., and Alter, G. (2021). The multifaceted roles of breast milk antibodies. Cell *184*, 1486–1499. https://doi.org/10.1016/j.cell.2021.02.031.

9. Rio-Aige, K., Azagra-Boronat, I., Castell, M., Selma-Royo, M., Collado, M.C., Rodríguez-Lagunas, M.J., and Pérez-Cano, F.J. (2021). The Breast Milk Immunoglobulinome. Nutrients *13*, 1810. https://doi.org/10.3390/nu13061810.

10. Abbassi-Ghanavati, M., Greer, L.G., and Cunningham, F.G. (2009). Pregnancy and Laboratory Studies: A Reference Table for Clinicians. Obstet. Gynecol. *114*, 1326–1331. https://doi.org/10.1097/AOG.0b013e3181c2bde8.

11. Lima, J., Cambridge, G., Vilas-Boas, A., Martins, C., Borrego, L.-M., and Leandro, M. (2019). Serum markers of B-cell activation in pregnancy during late gestation, delivery, and the postpartum period. Am. J. Reprod. Immunol. *81*, e13090. https://doi.org/10.1111/aji.13090.

12. Benster, B., and Wood, E.J. (1970). Immunoglobulin Levels in Normal Pregnancy and Pregnancy Complicated by Hypertension. J. Obstet. Gynaecol. Br. Commonw. *77*, 518–522. https://doi.org/10.1111/j.1471-0528.1970.tb03559.x.

13. Ganzevoort, W., Rep, A., Bonsel, G.J., de Vries, J.I.P., and Wolf, H. (2004). Plasma volume and blood pressure regulation in hypertensive pregnancy. J. Hypertens. *22*, 1235–1242. https://doi.org/10.1097/01.hjh.0000125436.28861.09.

14. Jennewein, M.F., Abu-Raya, B., Jiang, Y., Alter, G., and Marchant, A. (2017). Transfer of maternal immunity and programming of the newborn immune system. Semin. Immunopathol. *39*, 605–613. https://doi.org/10.1007/s00281-017-0653-x.

15. Noyola-Martínez, N., Halhali, A., and Barrera, D. (2019). Steroid hormones and pregnancy. Gynecol. Endocrinol. *35*, 376–384. https://doi.org/10.1080/09513590.2018.1564742.

16. Monteiro, C., Kasahara, T., Sacramento, P.M., Dias, A., Leite, S., Silva, V.G., Gupta, S., Agrawal, A., and Bento, C.A.M. (2021). Human pregnancy levels of estrogen and progesterone contribute to humoral immunity by activating TFH/B cell axis. Eur. J. Immunol. *51*, 167–179. https://doi.org/10.1002/eji.202048658.

17. Albrecht, M., Pagenkemper, M., Wiessner, C., Spohn, M., Lütgehetmann, M., Jacobsen, H., Gabriel, G., Zazara, D.E., Haertel, C., Hecher, K., et al. (2022). Infant immunity against viral infections is advanced by the placenta-dependent vertical transfer of maternal antibodies. Vaccine *40*, 1563–1571. https://doi.org/10.1016/j.vaccine.2020.12.049.

18. Robinson, D.P., and Klein, S.L. (2012). Pregnancy and pregnancy-associated hormones alter immune responses and disease pathogenesis. Horm. Behav. *62*, 263–271. https://doi.org/10.1016/j.yhbeh.2012.02.023.

19. Mor, G., Aldo, P., and Alvero, A.B. (2017). The unique immunological and microbial aspects of pregnancy. Nat. Rev. Immunol. *17*, 469–482. https://doi.org/10.1038/nri.2017.64.

20. Bertolaccini, M.L., and Sanna, G. (2016). Recent advances in understanding antiphospholipid syndrome. F1000Res. *5*, 2908. https://doi.org/10.12688/f1000research.9717.1.

21. Moghbel, M., Roth, A., Baptista, D., Miteva, K., Burger, F., Montecucco, F., Vuilleumier, N., Mach, F., and Brandt, K.J. (2022). Epitope of antiphospholipid antibodies retrieved from peptide microarray based on R39-R43 of β2-glycoprotein I. Res. Pract. Thromb. Haemost. *6*, e12828. https://doi.org/10.1002/rth2.12828.

22. Ekinci, E.I., Chiu, W.-L., Lu, Z.X., Sikaris, K., Churilov, L., Bittar, I., Lam, Q., Crinis, N., and Houlihan, C.A. (2015). A longitudinal study of thyroid autoantibodies in pregnancy: the importance of test timing. Clin. Endocrinol. *82*, 604–610. https://doi.org/10.1111/cen.12571.

23. Leavy, O. (2016). Maternal IgG promotes intestinal homeostasis. Nat. Rev. Immunol. *16*, 335. https://doi.org/10.1038/nri.2016.64.

24. Niewiesk, S. (2014). Maternal Antibodies: Clinical Significance, Mechanism of Interference with Immune Responses, and Possible Vaccination Strategies. Front. Immunol. *5*, 446. https://doi.org/10.3389/fimmu.2014.00446.

25. Watanabe, M., Iwatani, Y., Kaneda, T., Hidaka, Y., Mitsuda, N., Morimoto, Y., and Amino, N. (1997). Changes in T, B, and NK Lymphocyte Subsets During and After Normal Pregnancy. Am. J. Reprod. Immunol. *37*, 368–377. https://doi.org/10.1111/j.1600-0897.1997.tb00246.x.

26. Atyeo, C., Pullen, K.M., Bordt, E.A., Fischinger, S., Burke, J., Michell, A., Slein, M.D., Loos, C., Shook, L.L., Boatin, A.A., et al. (2021). Compromised SARS-CoV-2-specific placental antibody transfer. Cell *184*, 628–642.e10. https://doi.org/10.1016/j.cell.2020.12.027.

27. Cosma, S., Carosso, A.R., Corcione, S., Cusato, J., Borella, F., Antonucci, M., Marozio, L., Revelli, A., Preti, M., Ghisetti, V., et al. (2021). Longitudinal analysis of antibody response following SARS-CoV-2 infection in pregnancy: From the first trimester to delivery. J. Reprod. Immunol. *144*, 103285. https://doi.org/10.1016/j.jri.2021.103285.

28. Arora, M., and Lakshmi, R. (2021). Vaccines - safety in pregnancy. Best Pract. Res. Clin. Obstet. Gynaecol. *76*, 23–40. https://doi.org/10.1016/j.bpobgyn.2021.02.002.

29. Bardos, J., Hercz, D., Friedenthal, J., Missmer, S.A., and Williams, Z. (2015). A National Survey on Public Perceptions of Miscarriage. Obstet. Gynecol. *125*, 1313–1320. https://doi.org/10.1097/AOG.0000000000000859.

30. Abdalla, H.I., Burton, G., Kirkland, A., Johnson, M.R., Leonard, T., Brooks, A.A., and Studd, J.W. (1993). Pregnancy: Age, pregnancy and miscarriage: uterine versus ovarian factors. Hum. Reprod. *8*, 1512–1517. https://doi.org/10.1093/oxfordjournals.humrep.a138289.

31. Quenby, S., Gallos, I.D., Dhillon-Smith, R.K., Podesek, M., Stephenson, M.D., Fisher, J., Brosens, J.J., Brewin, J., Ramhorst, R., Lucas, E.S., et al. (2021). Miscarriage matters: the epidemiological, physical, psychological, and economic costs of early pregnancy loss. Lancet *397*, 1658–1667. https://doi.org/10.1016/S0140-6736(21)00682-6.

32. Al-Alami, Z., Abu-Huwaij, R., Hamadneh, S., and Taybeh, E. (2024). Understanding Miscarriage Prevalence and Risk Factors: Insights from Women in Jordan. Medicina (Kaunas) *60*, 1044. https://doi.org/10.3390/medicina60071044.

33. Schisterman, E.F., Silver, R.M., Lesher, L.L., Faraggi, D., Wactawski-Wende, J., Townsend, J.M., Lynch, A.M., Perkins, N.J., Mumford, S.L., and Galai, N. (2014). Preconception low-dose aspirin and pregnancy outcomes: results from the EAGeR randomised trial. Lancet *384*, 29–36. https://doi.org/10.1016/S0140-6736(14)60157-4.

34. Connell, M.T., Sjaarda, L.A., Radin, R.G., Kuhr, D., Mumford, S.L., Plowden, T.C., Silver, R.M., and Schisterman, E.F. (2017). The Effects of Aspirin in Gestation and Reproduction (EAGeR) Trial: A Story of Discovery. Semin. Reprod. Med. *35*, 344–352. https://doi.org/10.1055/s-0037-1606384.

35. Levine, L.D., Holland, T.L., Kim, K., Sjaarda, L.A., Mumford, S.L., and Schisterman, E.F. (2019). The role of aspirin and inflammation on reproduction: the EAGeR trial. Can. J. Physiol. Pharmacol. *97*, 187–192. https://doi.org/10.1139/cjpp-2018-0368.

36. Radin, R.G., Mumford, S.L., Silver, R.M., Lesher, L.L., Galai, N., Faraggi, D., Wactawski-Wende, J., Townsend, J.M., Lynch, A.M., Simhan, H.N., et al. (2015). Sex ratio following preconception low-dose aspirin in women with prior pregnancy loss. J. Clin. Investig. *125*, 3619–3626. https://doi.org/10.1172/JCI82357.

37. Sjaarda, L.A., Radin, R.G., Silver, R.M., Mitchell, E., Mumford, S.L., Wilcox, B., Galai, N., Perkins, N.J., Wactawski-Wende, J., Stanford, J.B., and Schisterman, E.F. (2017). Preconception Low-Dose Aspirin Restores Diminished Pregnancy and Live Birth Rates in Women With Low-Grade Inflammation: A Secondary Analysis of a Randomized Trial. J. Clin. Endocrinol. Metab. *102*, 1495–1504. https://doi.org/10.1210/jc.2016-2917.

38. Rossen, L.M., Ahrens, K.A., and Branum, A.M. (2018). Trends in Risk of Pregnancy Loss Among US Women, 1990–2011. Paediatr. Perinat. Epidemiol. *32*, 19–29. https://doi.org/10.1111/ppe.12417.

39. Xu, G.J., Kula, T., Xu, Q., Li, M.Z., Vernon, S.D., Ndung'u, T., Ruxrungtham, K., Sanchez, J., Brander, C., Chung, R.T., et al. (2015). Comprehensive serological profiling of human populations using a synthetic human virome. Science *348*, aaa0698. https://doi.org/10.1126/science.aaa0698.

40. Mina, M.J., Kula, T., Leng, Y., Li, M., de Vries, R.D., Knip, M., Siljander, H., Rewers, M., Choy, D.F., Wilson, M.S., et al. (2019). Measles virus infection diminishes preexisting antibodies that offer protection from other pathogens. Science *366*, 599–606. https://doi.org/10.1126/science.aay6485.

41. Shrock, E., Fujimura, E., Kula, T., Timms, R.T., Lee, I.-H., Leng, Y., Robinson, M.L., Sie, B.M., Li, M.Z., Chen, Y., et al. (2020). Viral epitope profiling of COVID-19 patients reveals cross-reactivity and correlates of severity. Science *370*, eabd4250. https://doi.org/10.1126/science.abd4250.

42. Mitchell, M.M., Leng, Y., Boppana, S., Britt, W.J., Gutierrez Sanchez, L.H., and Elledge, S.J. (2023). Signatures of AAV-2 immunity are enriched in children with severe acute hepatitis of unknown etiology. Sci. Transl. Med. *15*, eadh9917. https://doi.org/10.1126/scitranslmed.adh9917.

43. Rao, V.N., and Coelho, C.H. (2025). Public antibodies: convergent signatures in human humoral immunity against pathogens. mBio *16*, e02247-24. https://doi.org/10.1128/mbio.02247-24.

44. Credle, J.J., Gunn, J., Sangkhapreecha, P., Monaco, D.R., Zheng, X.A., Tsai, H.-J., Wilbon, A., Morgenlander, W.R., Rastegar, A., Dong, Y., et al. (2022). Unbiased discovery of autoantibodies associated with severe COVID-19 via genome-scale self-assembled DNA-barcoded protein libraries. Nat. Biomed. Eng. *6*, 992–1003. https://doi.org/10.1038/s41551-022-00925-y.

45. Larman, H.B., Zhao, Z., Laserson, U., Li, M.Z., Ciccia, A., Gakidis, M.A.M., Church, G.M., Kesari, S., LeProust, E.M., Solimini, N.L., and Elledge, S.J. (2011). Autoantigen discovery with a synthetic human peptidome. Nat. Biotechnol. *29*, 535–541. https://doi.org/10.1038/nbt.1856.

46. Mohan, D., Wansley, D.L., Sie, B.M., Noon, M.S., Baer, A.N., Laserson, U., and Larman, H.B. (2018). PhIP-Seq characterization of serum antibodies using oligonucleotide-encoded peptidomes. Nat. Protoc. *13*, 1958–1978. https://doi.org/10.1038/s41596-018-0025-6.

47. Eshleman, S.H., Laeyendecker, O., Kammers, K., Chen, A., Sivay, M.V., Kottapalli, S., Sie, B.M., Yuan, T., Monaco, D.R., Mohan, D., et al. (2019). Comprehensive Profiling of HIV Antibody Evolution. Cell Rep. *27*, 1422–1433.e4. https://doi.org/10.1016/j.celrep.2019.03.097.

48. Brown, E.P., Licht, A.F., Dugast, A.-S., Choi, I., Bailey-Kellogg, C., Alter, G., and Ackerman, M.E. (2012). High-throughput, multiplexed IgG subclassing of antigen-specific antibodies from clinical samples. J. Immunol. Methods *386*, 117–123. https://doi.org/10.1016/j.jim.2012.09.007.

49. Atyeo, C., DeRiso, E.A., Davis, C., Bordt, E.A., De Guzman, R.M., Shook, L.L., Yonker, L.M., Fasano, A., Akinwunmi, B., Lauffenburger, D.A., et al. (2021). COVID-19 mRNA vaccines drive differential antibody Fc-functional profiles in pregnant, lactating, and non-pregnant women. Sci. Transl. Med. *13*, eabi8631. https://doi.org/10.1126/scitranslmed.abi8631.

50. Ander, S.E., Diamond, M.S., and Coyne, C.B. (2019). Immune responses at the maternal-fetal interface. Sci. Immunol. *4*, eaat6114. https://doi.org/10.1126/sciimmunol.aat6114.

51. PrabhuDas, M., Bonney, E., Caron, K., Dey, S., Erlebacher, A., Fazleabas, A., Fisher, S., Golos, T., Matzuk, M., McCune, J.M., et al. (2015). Immune mechanisms at the maternal-fetal interface: perspectives and challenges. Nat. Immunol. *16*, 328–334. https://doi.org/10.1038/ni.3131.

52. Palmeira, P., Quinello, C., Silveira-Lessa, A.L., Zago, C.A., and Carneiro-Sampaio, M. (2012). IgG Placental Transfer in Healthy and Pathological Pregnancies. Clin. Dev. Immunol. *2012*, 1–13. https://doi.org/10.1155/2012/985646.

53. Hosono, Y., Sie, B., Pinal-Fernandez, I., Pak, K., Mecoli, C.A., Casal-Dominguez, M., Warner, B.M., Kaplan, M.J., Albayda, J., Danoff, S., et al. (2023). Coexisting autoantibodies against transcription factor Sp4 are associated with decreased cancer risk in patients with dermatomyositis with anti-TIF1γ autoantibodies. Ann. Rheum. Dis. *82*, 246–252. https://doi.org/10.1136/ard-2022-222441.

54. Xu, G.J., Shah, A.A., Li, M.Z., Xu, Q., Rosen, A., Casciola-Rosen, L., and Elledge, S.J. (2016). Systematic autoantigen analysis identifies a distinct subtype of scleroderma with coincident cancer. Proc. Natl. Acad. Sci. USA *113*, E7526–E7534. https://doi.org/10.1073/pnas.1615990113.

55. Triulzi, D.J., Kleinman, S., Kakaiya, R.M., Busch, M.P., Norris, P.J., Steele, W.R., Glynn, S.A., Hillyer, C.D., Carey, P., Gottschall, J.L., et al. (2009). The effect of previous pregnancy and transfusion on HLA alloimmunization in blood donors: implications for a transfusion-related acute lung injury risk reduction strategy. Transfusion *49*, 1825–1835. https://doi.org/10.1111/j.1537-2995.2009.02206.x.

56. Persson, G., Picard, C., Marin, G., Isgaard, C., Stæhr, C.S., Molinari, N., Chiaroni, J., Lebech, M., Hviid, T.V.F., and Di Cristofaro, J. (2021). Maternal HLA Ib Polymorphisms in Pregnancy Allo-Immunization. Front. Immunol. *12*, 657217. https://doi.org/10.3389/fimmu.2021.657217.

57. Meuleman, T., van Beelen, E., Kaaja, R.J., van Lith, J.M.M., Claas, F.H.J., and Bloemenkamp, K.W.M. (2016). HLA-C antibodies in women with recurrent miscarriage suggests that antibody mediated rejection is one of the mechanisms leading to recurrent miscarriage. J. Reprod. Immunol. *116*, 28–34. https://doi.org/10.1016/j.jri.2016.03.003.

58. Nielsen, H.S., Witvliet, M.D., Steffensen, R., Haasnoot, G.W., Goulmy, E., Christiansen, O.B., and Claas, F. (2010). The presence of HLA-antibodies in recurrent miscarriage patients is associated with a reduced chance of a live birth. J. Reprod. Immunol. *87*, 67–73. https://doi.org/10.1016/j.jri.2010.05.006.

59. Aghaeepour, N., Ganio, E.A., Mcilwain, D., Tsai, A.S., Tingle, M., Van Gassen, S., Gaudilliere, D.K., Baca, Q., McNeil, L., Okada, R., et al. (2017). An immune clock of human pregnancy. Sci. Immunol. *2*, eaan2946. https://doi.org/10.1126/sciimmunol.aan2946.

60. Wang, W., Sung, N., Gilman-Sachs, A., and Kwak-Kim, J. (2020). T Helper (Th) Cell Profiles in Pregnancy and Recurrent Pregnancy Losses: Th1/Th2/Th9/Th17/Th22/Tfh Cells. Front. Immunol. *11*, 2025. https://doi.org/10.3389/fimmu.2020.02025.

61. Förger, F., and Villiger, P.M. (2020). Immunological adaptations in pregnancy that modulate rheumatoid arthritis disease activity. Nat. Rev. Rheumatol. *16*, 113–122. https://doi.org/10.1038/s41584-019-0351-2.

62. Rothermel, A.L., Gilbert, K.M., and Weigle, W.O. (1991). Differential abilities of Th1 and Th2 to induce polyclonal B cell proliferation. Cell. Immunol. *135*, 1–15. https://doi.org/10.1016/0008-8749(91)90249-b.

63. (1989). Transforming growth factor beta induces IgA production and acts additively with interleukin 5 for IgA production. J. Exp. Med. *170*, 1415–1420. https://doi.org/10.1084/jem.170.4.1415.

64. Jourdan, M., Caraux, A., De Vos, J., Fiol, G., Larroque, M., Cognot, C., Bret, C., Duperray, C., Hose, D., and Klein, B. (2009). An in vitro model of differentiation of memory B cells into plasmablasts and plasma cells including detailed phenotypic and molecular characterization. Blood *114*, 5173–5181. https://doi.org/10.1182/blood-2009-07-235960.

65. Wu, W., Sun, M., Chen, F., Cao, A.T., Liu, H., Zhao, Y., Huang, X., Xiao, Y., Yao, S., Zhao, Q., et al. (2017). Microbiota metabolite short chain fatty acid acetate promotes intestinal IgA response to microbiota which is mediated by GPR43. Mucosal Immunol. *10*, 946–956. https://doi.org/10.1038/mi.2016.114.

66. Barrientos, G., Fahlbusch, F.B., and Conrad, M.L. (2026). Microbes, molecules, and the maternal–fetal interface: rethinking the gut–placenta axis. Trends Immunol. *0*. https://doi.org/10.1016/j.it.2026.03.010.

67. Andreas, N.J., Kampmann, B., and Mehring Le-Doare, K. (2015). Human breast milk: A review on its composition and bioactivity. Early Hum. Dev. *91*, 629–635. https://doi.org/10.1016/j.earlhumdev.2015.08.013.

68. Li, Y., Jin, L., and Chen, T. (2020). The Effects of Secretory IgA in the Mucosal Immune System. Biomed Res. Int. *2020*, 2032057. https://doi.org/10.1155/2020/2032057.

69. Bollig, K.J., Senapati, S., Takacs, P., Robins, J.C., Haisenleder, D.J., Beer, L.A., Speicher, D.W., Koelper, N.C., and Barnhart, K.T. (2024). Evaluation of novel biomarkers for early pregnancy outcome prediction. Biol. Reprod. *110*, 548–557. https://doi.org/10.1093/biolre/ioad162.

70. Benedetto, C., Tibaldi, C., Marozio, L., Marini, S., Masuelli, G., Pelissetto, S., Sozzani, P., and Latino, M.A. (2004). Cervicovaginal infections during pregnancy: epidemiological and microbiological aspects. J. Matern. Fetal Neonatal Med. *16*, 9–12. https://doi.org/10.1080/14767050410001727107.

71. Srinivas, S.K., Ma, Y., Sammel, M.D., Chou, D., McGrath, C., Parry, S., and Elovitz, M.A. (2006). Placental inflammation and viral infection are implicated in second trimester pregnancy loss. Am. J. Obstet. Gynecol. *195*, 797–802. https://doi.org/10.1016/j.ajog.2006.05.049.

72. Baud, D., Regan, L., and Greub, G. (2008). Emerging role of Chlamydia and Chlamydia-like organisms in adverse pregnancy outcomes. Curr. Opin. Infect. Dis. *21*, 70–76. https://doi.org/10.1097/QCO.0b013e3282f3e6a5.

73. Allanson, B., Jennings, B., Jacques, A., Charles, A.K., Keil, A.D., and Dickinson, J.E. (2010). Infection and fetal loss in the mid-second trimester of pregnancy. Aust. N. Z. J. Obstet. Gynaecol. *50*, 221–225. https://doi.org/10.1111/j.1479-828X.2010.01148.x.

74. Iwasenko, J.M., Howard, J., Arbuckle, S., Graf, N., Hall, B., Craig, M.E., and Rawlinson, W.D. (2011). Human cytomegalovirus infection is detected frequently in stillbirths and is associated with fetal thrombotic vasculopathy. J. Infect. Dis. *203*, 1526–1533. https://doi.org/10.1093/infdis/jir121.

75. Njue, A., Coyne, C., Margulis, A.V., Wang, D., Marks, M.A., Russell, K., Das, R., and Sinha, A. (2020). The Role of Congenital Cytomegalovirus Infection in Adverse Birth Outcomes: A Review of the Potential Mechanisms. Viruses *13*, 20. https://doi.org/10.3390/v13010020.

76. Giakoumelou, S., Wheelhouse, N., Cuschieri, K., Entrican, G., Howie, S.E.M., and Horne, A.W. (2016). The role of infection in miscarriage. Hum. Reprod. Update *22*, 116–133. https://doi.org/10.1093/humupd/dmv041.

77. Basonidis, A., Liberis, A., Daniilidis, A., Petousis, S., and Dinas, K. (2020). Human papilloma virus infection and miscarriage: is there an association? Taiwan. J. Obstet. Gynecol. *59*, 656–659. https://doi.org/10.1016/j.tjog.2020.07.005.

78. Montague, B.T., Wipperman, M.F., Hooper, A.T., Hamon, S.C., Crow, R., Elemo, F., Hersh, L., Langdon, S., Hamilton, J.D., O'Brien, M.P., and Simões, E.A.F. (2022). Anti-SARS-CoV-2 IgA Identifies Asymptomatic Infection in First Responders. J. Infect. Dis. *225*, 578–586, jiab524. https://doi.org/10.1093/infdis/jiab524.

79. Fröberg, J., and Diavatopoulos, D.A. (2021). Mucosal immunity to severe acute respiratory syndrome coronavirus 2 infection. Curr. Opin. Infect. Dis. *34*, 181–186. https://doi.org/10.1097/QCO.0000000000000724.

80. Reinwald, M., Deckert, P.M., Ritter, O., Andresen, H., Schreyer, A.G., Weylandt, K.H., Dammermann, W., and Lüth, S. (2021). Prevalence and Course of IgA and IgG Antibodies against SARS-CoV-2 in Healthcare Workers during the First Wave of the COVID-19 Outbreak in Germany: Interim Results from an Ongoing Observational Cohort Study. Healthcare (Basel) *9*, 498. https://doi.org/10.3390/healthcare9050498.

81. Levy, R.A., Pinto, C.M., Domingues, V., Jesús, G. de, García-Carrasco, M., Jesús, N.R. de, and Cervera, R. (2013). Systemic autoimmune diseases and pregnancy. In Autoimmunity: From Bench to Bedside [Internet] (El Rosario University Press).

82. O'Donoghue, K. (2011). Pregnancy and the risk of autoimmune disease. Chimerism *2*, 84–85. https://doi.org/10.4161/chim.17771.

83. Breedveld, A., and van Egmond, M. (2019). IgA and FcαRI: Pathological Roles and Therapeutic Opportunities. Front. Immunol. *10*, 553. https://doi.org/10.3389/fimmu.2019.00553.

84. van Delft, M.A.M., Aleyd, E., van der Mast, R., de Jong, N., Boon, L., Simons, P.J., and van Egmond, M. (2023). Antagonizing FcαR1 (CD89) as treatment in IgA-mediated chronic inflammation and autoimmunity. Front. Immunol. *14*, 1118539. https://doi.org/10.3389/fimmu.2023.1118539.

85. van der Steen, L.P., Bakema, J.E., Sesarman, A., Florea, F., Tuk, C.W., Kirtschig, G., Hage, J.J., Sitaru, C., and van Egmond, M. (2012). Blocking Fcα receptor I on granulocytes prevents tissue damage induced by IgA autoantibodies. J. Immunol. *189*, 1594–1601. https://doi.org/10.4049/jimmunol.1101763.

86. Allen, K.C., Warner, S., Teague, H.L., Ramos-Benitez, M.J., Miao, R., Tian, X., Reger, R., Burbelo, P.D., Pang, C.W.J., Kanthi, Y., et al. (2025). SARS-CoV-2 Immune Complex-Mediated Neutrophil Activation. Open Forum Infect. Dis. *12*, ofaf199. https://doi.org/10.1093/ofid/ofaf199.

87. Ahmadi, P., Janzadeh, A., Honardoost, M., Taherkhani, S., Doyle, D., and Mojarad, N. (2025). The role of IL-39 in autoimmune diseases: From general to immunopathogenesis. Cytokine *192*, 156969. https://doi.org/10.1016/j.cyto.2025.156969.

88. Hernández González, L.L., Pérez-Campos Mayoral, L., Hernández-Huerta, M.T., Mayoral Andrade, G., Martínez Cruz, M., Ramos-Martínez, E., Pérez-Campos Mayoral, E., Cruz Hernández, V., Antonio García, I., Matias-Cervantes, C.A., et al. (2024). Targeting Neutrophil Extracellular Trap Formation: Exploring Promising Pharmacological Strategies for the Treatment of Preeclampsia. Pharmaceuticals *17*, 605. https://doi.org/10.3390/ph17050605.

89. Deshmukh, H., and Way, S.S. (2019). Immunological Basis for Recurrent Fetal Loss and Pregnancy Complications. Annu. Rev. Pathol. *14*, 185–210. https://doi.org/10.1146/annurev-pathmechdis-012418-012743.

90. Shrock, E.L., Shrock, C.L., and Elledge, S.J. (2022). VirScan: High-throughput Profiling of Antiviral Antibody Epitopes. Bio. Protoc. *12*, e4464. https://doi.org/10.21769/BioProtoc.4464.

91. Langmead, B., Trapnell, C., Pop, M., and Salzberg, S.L. (2009). Ultrafast and memory-efficient alignment of short DNA sequences to the human genome. Genome Biol. *10*, R25. https://doi.org/10.1186/gb-2009-10-3-r25.

92. Li, H., Handsaker, B., Wysoker, A., Fennell, T., Ruan, J., Homer, N., Marth, G., Abecasis, G., and Durbin, R.; 1000 Genome Project Data Processing Subgroup (2009). The Sequence Alignment/Map format and SAMtools. Bioinformatics *25*, 2078–2079. https://doi.org/10.1093/bioinformatics/btp352.

93. Buchfink, B., Reuter, K., and Drost, H.-G. (2021). Sensitive protein alignments at tree-of-life scale using DIAMOND. Nat. Methods *18*, 366–368. https://doi.org/10.1038/s41592-021-01101-x.

94. R Core Team R: A Language and Environment for Statistical Computing. (R Foundation for Statistical Computing).

95. Landau, W. (2021). The targets R package: a dynamic Make-like function-oriented pipeline toolkit for reproducibility and high-performance computing. JOSS *6*, 2959. https://doi.org/10.21105/joss.02959.

96. Wickham, H., Averick, M., Bryan, J., Chang, W., McGowan, L., François, R., Grolemund, G., Hayes, A., Henry, L., Hester, J., et al. (2019). Welcome to the Tidyverse. JOSS *4*, 1686. https://doi.org/10.21105/joss.01686.

97. Fairbanks, M. (2024). tidytable: Tidy Interface to "data.table."

98. Kassambara, A. (2023). ggpubr: "ggplot2" Based Publication Ready Plots. Version 0.6.0.

STAR★METHODS

KEY RESOURCES TABLE

REAGENT or RESOURCE	SOURCE	IDENTIFIER
**Antibodies**		
Goat anti-human IgA-BIOT	Southern Biotech	Cat#2050-08; RRID:AB_2795706
**Biological samples**		
EAGeR trial participant sera	National Institute of Child Health and Human Development	N/A
**Chemicals, peptides, and recombinant proteins**		
Protein A Dynabeads	Thermo Fisher, Invitrogen	Cat#10008D
Protein G Dynabeads	Thermo Fisher, Invitrogen	Cat#10009D
Pierce Streptavidin Beads	Thermo Fisher	Cat#C88817
**Critical commercial assays**		
LabScreen Mixed	One Lambda	Cat#LSM12
HuSight (MIPSA)	Infinity Bio	https://www.infinitybio.com/mipsa/
**Deposited data**		
Antibody repertoire profiling data	This paper	GEO: GSE314505
**Oligonucleotides**		
Primer: IS7: ACACTCTTT CCCTACACGACTCCAGTCAGG TGTGATGCTC	Shrock et al.[90]	N/A
Primer: IS8: GTGACTGGAG TTCAGACGTGTGCTCTTCCGA TCCGAGCTTATCGTCGTCATCC	Shrock et al.[90]	N/A
Primer: IS4: AATGATACGGC GACCACCGAGATCTACACTCTT TCCCTACACGACTCCAGT	Shrock et al.[90]	N/A
Primer: Index: CAAGCAGAA GACGGCATACGAGATNNNNN NNNGTGACTGGAGTTCAGACGTGT	Shrock et al.[90]	N/A
Primer: T7-Illumina-READ1-A: TGCTCGGGGATCCAGGAATT CCGCTGCGT	Shrock et al.[90]	N/A
**Software and algorithms**		
Bowtie	Langmead et al.[91]	https://bowtie-bio.sourceforge.net/index.shtml
Samtools	Li et al.[92]	https://www.htslib.org/
DIAMOND	Buchfink et al.[93]	https://github.com/bbuchfink/diamond
R (version 4.2.1)	R Core Team.[94]	https://www.r-project.org/
Targets R package	Landau.[95]	https://books.ropensci.org/targets/
Tidyverse r package	Wickham et al.[96]	https://tidyverse.org/
Tidytable r package	Fairbanks.[97]	https://cran.r-project.org/package=tidytable
Ggpubr r package	Kassambra.[98]	https://rpkgs.datanovia.com/ggpubr/
**Other**		
VirScan library	Xu et al.[39]	N/A
pep 2.2 library	Larman et al.[45]	N/A
pep 4 library	This paper	N/A
Nunc 96-Well Polypropylene DeepWell Plates	Thermo Fisher	Cat#260251

EXPERIMENTAL MODEL AND STUDY PARTICIPANT DETAILS

Human samples

Serum samples were obtained from 119 women originally participating in the EAGeR clinical trial[33] from the National Institute of Child Health and Human Development as indicated in the key resources table. As described initially, the Institutional Review Board for each clinical center approved the study, and written informed consent was obtained from participants.[33] For all participants, two pre-pregnancy time points were included, approximately 1 month apart, representing the two monthly visits immediately preceding a positive pregnancy test. Additionally, samples were included from gestational weeks 4, 8, 12, 20, and 36, along with one sample collected within a few days post-partum, typically within 48h. Of the 119 participants in Cohort 1, 19 had pregnancies that resulted in miscarriage. Samples were included until the latest pregnancy time point available. Anonymized demographic information (age, sex, pregnancy history, etc.) for EAGeR study participants whose samples are included in the present study are provide in Table S1, and analysis of covariates is included in Figure S1A.

METHOD DETAILS

Phage immunoprecipitation and sequencing

The phage immunoprecipitation and sequencing assay was performed as previously described.[39–41,45,46] T7 bacteriophage was used to display linear peptides representing the VirScan library. Serum samples were evenly diluted with PBS and a constant volume was assayed such that approximately 2 μg of antibody would be included from a typical serum sample. Diluted serum was mixed with peptide-displaying phage and incubated with end-over-end rotation. We used protein A and protein G Dynabeads (Invitrogen) to capture IgG, following the standard protocol. To capture IgA, we used 4 μg of Goat Anti-Human IgA-BIOT antibody overnight (Southern Biotech), followed by 20 μL of Pierce Streptavidin Magnetic Beads (Thermo-Fisher). Then, from each sample, DNA from the immunoprecipitated phage was amplified for the peptide-coding insert. A sample-specific barcode was attached by polymerase chain reaction,[90] and pooled samples were sequenced using an Illumina NextSeq. Samples were screened in duplicate, and each plate included no-serum mock immunoprecipitations as controls. All samples were screened by IgG VirScan. A subset of samples, including all miscarriage samples and samples from ~20% of birth-outcome pregnancies were screened by IgA VirScan. The utilized VirScan peptide library contains 115,753 uniquely barcoded 56-mers with 50% overlap between adjacent tiles tiling across proteins from viruses that infect humans. Two human peptidome libraries were used in this study. One contains ~260,000 90-mer peptides tiling across human proteins with 50% overlap between adjacent tiles as previously described. For a subset of samples, IgG PhIP-Seq with a human peptidome was performed with an augmented version of the library, pep4, containing ~600,000 90-mer peptide tiles, tiling across the same human proteome but with two barcodes representing each peptide and some additional control sequences. Samples were processed in 96-well plates, and so multiple 96-well plates were required to accommodate the 878 samples. To avoid the possibility that signal changes observed over the course of gestational time could be explained by batch effect, samples from each participant's time course were always processed on the same 96-well plate. Additionally, VirScan samples were screened in duplicate, and correlation between replicates was performed as quality control. Normalized read counts from VirScan replicates correlated strongly, consistent with previous VirScan studies, with a Pearson correlation mean ± SD 0.85 ± 0.15. Furthermore, a strong correlation was also observed across different time points from the same participant's time course, far beyond that observed from samples from mismatched individuals (Figures S1F and S1G).

Luminex

Antibody quantification was performed as described previously.[26,48] Briefly, a multiplexed Luminex assay was used to determine relative titer of antigen-specific isotypes and subclasses using the antigens described in Table S1. Antigens were covalently linked to carboxyl-modified Magplex© Luminex beads using Sulfo-NHS (N-hydroxysulfosuccinimide, Pierce) and ethyl dimethylaminopropyl carbodiimide hydrochloride (EDC). Antigen-coupled microspheres were blocked, washed, resuspended in PBS, and stored at 4°C. To form immune complexes, appropriately diluted plasma (1:100 for IgG2/3, IgA, IgM; 1:500 for IgG1) was added to the antigen-coupled microspheres, and plates were incubated overnight at 4°C, shaking at 700 rpm. The following day, plates were washed with 0.1% BSA 0.02% Tween 20. PE-coupled mouse anti-human detection antibodies (Southern Biotech) were used to detect antigen-specific antibody binding. Fluorescence was acquired using an Intellicyt iQue.

Profiling antibodies against HLA

A commercially available kit from OneLambda, "LabScreen Mixed" was purchased. Instead of the provided PE anti-human IgG detection antibody, we used a PE goat anti-human IgA antibody to adapt the assay for detecting IgA antibodies that target HLA. An optimization test was performed using the IgA detection antibody and a concentration series of a pool of week 12 serum samples. Based on this, a serum concentration was chosen and used to screen the remaining serum samples using OneLambda's protocol.

MIPSA

IgG and IgA MIPSA (Molecular indexing of proteins by self-assembly) assays were performed by Infinity Bio as previously described[44]: a library of proteins was tagged with unique DNA barcodes using the HaloTag-HaloLigand interaction. This library

was incubated with human serum to allow antibodies to bind, then immunoprecipitated. The DNA barcodes were then amplified and sequenced.

ELISA

Plates were coated with 100 ng/mL per well of anti-human IgG, anti-human IgM, or anti-human IgA. Plates were incubated overnight at 4°C and washed in wash buffer (phosphate-buffered saline with 0.05% Tween 20). Plates were blocked with a 5% FBS solution then washed again with wash buffer. Serum samples were diluted and added to the plates. Plates were incubated on ice for 30 min. After incubation, plates were washed, and anti-human IgG, anti-human IgM, or anti-human IgA coupled to horseradish peroxidase (HRP) (Bethyl Laboratories) was added for detection. Plates were incubated for 1 h at room temperature in the dark and washed. The ELISA was developed with TMB and stopped with sulfuric acid. The signal was read at 450 nm.

QUANTIFICATION AND STATISTICAL ANALYSIS

Data analysis

Read counts from Illumina sequencing were aligned to the VirScan and PhIP-Seq libraries using bowtie[91] and processed into read counts with samtools.[92] Peptide enrichment scores were calculated as z-scores as previously described.[40–42] Briefly, null distributions were defined by binning peptides into groups of 300 or larger based on abundance in mock immunoprecipitation reactions, and per-sample per-peptide enrichment scores were calculated as the deviation from the mean of the middle 90% of a sample's scores within each peptide bin. *Z* score thresholds for calling significant enrichment were chosen as $Z > 5$ for IgG and $Z > 10$ for IgA, such that roughly 1% of library scores as enriched for an average sample. The peptides that score above these thresholds in both replicates for a sample are termed "hits." A small number of samples with fewer than 100 enriched peptides (~0.1% of the library) were considered QC failures and excluded from subsequent analysis. From each sample set of peptide hits, we calculate a non-redundant breadth measure representing the number of distinct epitopes for which a set of peptide hits provides some supporting evidence. Briefly, the library peptides were queries for sequence similarity against each other using DIAMOND,[93] a network graph was constructed with nodes representing peptide and edges representing alignments between peptides, and the size of the maximal independent set of nodes was computed for each sample.

For some analyses, measurements were normalized per person to pre-pregnancy levels. For each participant, two initial samples (denoted "w0" for a sample taken around the last menses prior to a positive pregnancy test and "Pre" for a sample taken four weeks prior to w0) are averaged to define a pre-pregnancy immune baseline. The mean of these two scores was taken, and then the ratio was calculated between the score at the time point of interest and the pre-pregnancy average. Some ratios were taken with pseudo-counting, for example (A+1)/(B+1), with specific details described where applicable. For LabScreen Mixed assay data, each sample's readout consisted of median fluorescence intensity for 19 distinct Luminex beads. After checking replicate correlation, replicate data were combined by taking a weighted average of scores for each analyte, weighted by the number of read counts per replicate. Deconvolution of bead readouts to measures for individual HLAs was performed by taking the mean of scores from beads that had that specific HLA protein on its bead surface.

Data processing, statistical analysis, and figure generation were performed in R version 4.2.1,[94] using packages targets,[95] tidyverse,[96] tidytable,[97] and ggpubr.[98] Figures were arranged using Adobe Illustrator 2022. The Wilcoxon rank-sum test, Welch's *t* test, Fisher's exact test, and chi-squared test were performed as described in the text, with statistical parameters reported in the figure legend corresponding to each analysis. Tests such as Welch's *t* test and nonparametric tests were chosen to minimize the possibility of data violating test assumptions. Multiple hypothesis correction was performed using the 'p.adjust' function and either Benjamini-Hochberg "FDR" correction or Bonferroni correction as specified in the text. Library metadata is provided in Tables S10, S11, S12, and S13.

Multiple hypothesis correction for IgA STORM

To evaluate potential statistical significance of IgA STORM with multiple hypothesis correction, we performed Fisher's exact test for each antibody-antigen combination, comparing week 8–12 samples from pregnancies ending in live birth and miscarriage, based on the 95th percentile of fold-change scores in the birth-outcome, with a one-sided alternative hypothesis that miscarriage is associated with higher frequency of above-threshold scores. We then performed multiple hypothesis correction using the Benjamini-Hochberg method. The associations that remained statistically significant after correction are IgA-Rubella, IgA-Pertussis, IgA-CMV, IgA-HSV-1, IgA-EBV, IgA-norovirus, IgA-pneumo, and IgA-RSV. We noticed more noise in the influenza data, likely related to mid-pregnancy vaccinations. Thus, as a conservative measure, to exclude the effect of vaccination, we focused subsequent analysis on antibody-antigen combinations with significant FDRs for which there are not common vaccines.

Collapsing LabScreen HLA data to a single strongest score

For each of 12 beads containing class I antigens and each of 5 beads containing class II antigens, we centered and scaled our anti-HLA scores across our tested population and calculated a *Z* score for each sample. Then for each week 8–12 sample, the maximum *Z* score across all antigens is chosen to represent the strongest indication of anti-HLA IgA response. Then for class I and class II antigens separately, this maximum anti-HLA score is compared across pregnancy outcomes.

Z-scores

High-throughput serology on this scale is an area of ongoing development, and we have used our best judgment to adopt responsible practices. The thresholds used in this study for defining peptide enrichment by VirScan/PhIP-Seq and for scoring differences in between pregnancies that end in miscarriage to those that end in live birth have precedent from previous studies. For *Z* score, our lab and others have used a range of *Z* score enrichment thresholds, from $Z > 3.5$ across duplicates[40–42] to $Z > 10$ without replicates.[53] We choose a threshold of $Z > 5$ across duplicates but find similar trends regardless of chosen *Z* score threshold (Figure S11). For calling aberrant IgA signal in samples preceding miscarriage, we used thresholds defined by the 95th percentile of scores in the control population of birth-outcome pregnancies. This 95th percentile threshold has precedent in a previous PhIP-Seq study[53] and we additionally show that there is a strong difference in the distribution of IgA signal in first-trimester samples preceding miscarriage and first-trimester samples preceding live birth regardless of our chosen threshold (Figure S8).

Covariates

Additionally, we assessed available metadata from the EAGeR as possible covariates. We found that participants who received a documented influenza vaccination during pregnancy show a marked increase in anti-influenza antibodies, particularly IgG that binds to the neonatal Fc receptor (FcRn) (Figure S3I). We do not observe a significant association with maternal age, maternal BMI, maternal race, gestational diabetes, preeclampsia, number of prior pregnancies, number of prior miscarriages, birthweight, month of delivery, sample collection site, documented allergic reaction, or documented rash with any of our major findings of antibody breadth increase, IgA PRIME, and IgA STORM. This is partially explained by very low documented incidence of gestational diabetes, preeclampsia, allergy, and rash in our cohort.

Asterisks

For some figures, we represent *p*-values adjusted for multiple hypothesis testing with asterisks to reduce visual clutter. The FDR thresholds corresponding to each quantity of asterisks are described in corresponding figure legends. Except where otherwise specified, we use the following FDR thresholds: *<0.05, **<0.01, ***<0.001, ****<0.0001, *****<0.00001.

Cell Reports Medicine, Volume *7*

Supplemental information

Humoral pregnancy analyses reveal strategies

for fetal protection and aberrancy in miscarriage

Brandon M. Sie, Yumei Leng, Nadege Nziza, Moriah M. Mitchell, Nicholas E. Webb, Yunjoo Lee, Sunni L. Mumford, Enrique F. Schisterman, H. Benjamin Larman, Boris Julg, Galit Alter, and Stephen J. Elledge

Supplementary Figure S1

A

Pregnancy Outcome	Participants (n)	Age (years)	BMI	Prior Pregnancies (n)	Prior Pregnancy Losses (n)	Birthweight (g)	Gestational Age at End of Pregnancy (weeks)
Birth	100	28.3 ± 4.0 (19.5 -37.9)	24.8 ± 4.7 (17.1 -40.2)	2.3 ± 1.1 (1-7)	1.3 ± 0.5 (1-2)	3435 ± 444 (2393 -4990)	39.2 ± 1.3 (37-44.9)
Loss	19	29.4 ± 4.9 (20.3 -39.8)	25.5 ± 6.2 (17.8 -40.4)	2.3 ± 1.2 (1-5)	1.4 ± 0.5 (1-2)	NA	10.6 ± 1.8 (8-14.6)
Wilcoxon FDR		0.68	0.95	0.95	0.68		

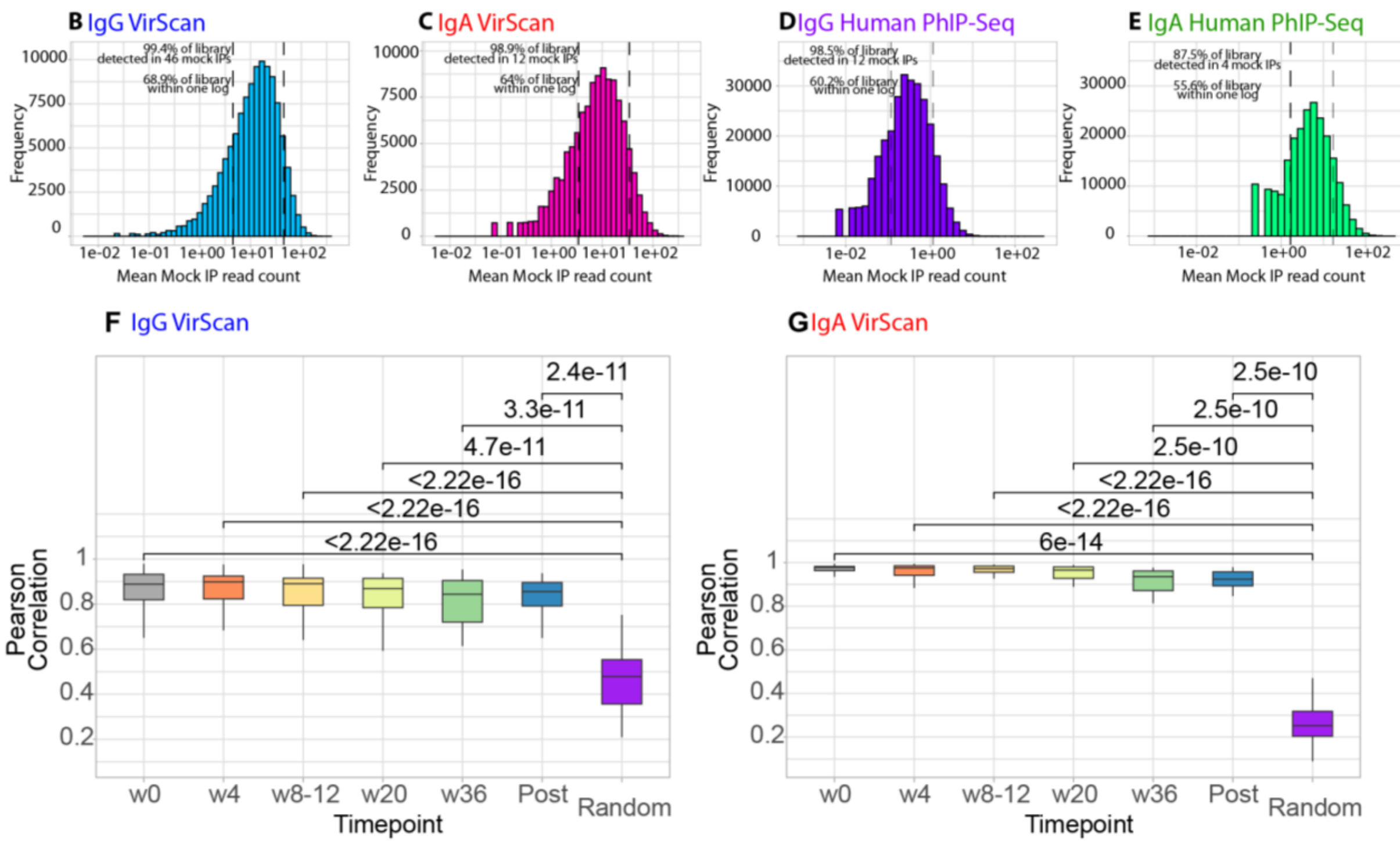

Fig. S1. Sample and library background information. Related to Figure 1.
(**A**) Demographic information for the EAGeR trial participants with samples screened in the current study, separated by pregnancy outcome. Values are listed as "mean ± standard deviation (minimum-maximum)." FDR values at the bottom of the chart show the Benjamini-Hochberg corrected p-values for a Wilcoxon rank-sum test comparing birth and loss outcome values for the metric specified at the top of that column. (**B-E**) Distribution of Illumina sequencing reads, averaged across no-serum mock immunoprecipitation reactions for (B) IgG VirScan, (C) IgA VirScan (D) IgG human PhIP-Seq, (E) IgA human PhIP-Seq. Dotted lines show a one log interval. The percentage of the library observed within one log and across all mock IP reactions are specified. For instances that multiple oligos code for the same peptide sequence, read counts were summed across redundant oligos. (**F-G**) Correlation of reads per million (RPM) for samples across gestational time compared to pre-pregnancy baseline for (F) IgG VirScan and (G) IgA VirScan. The "Random" category is comprised of one comparison each between a pre-pregnancy (Pre) sample and one other pre-pregnancy sample from different participant. P-values indicate the significance of a two-sided Wilcoxon rank-sum test comparing each of the two bracketed populations.

Supplementary Figure S2

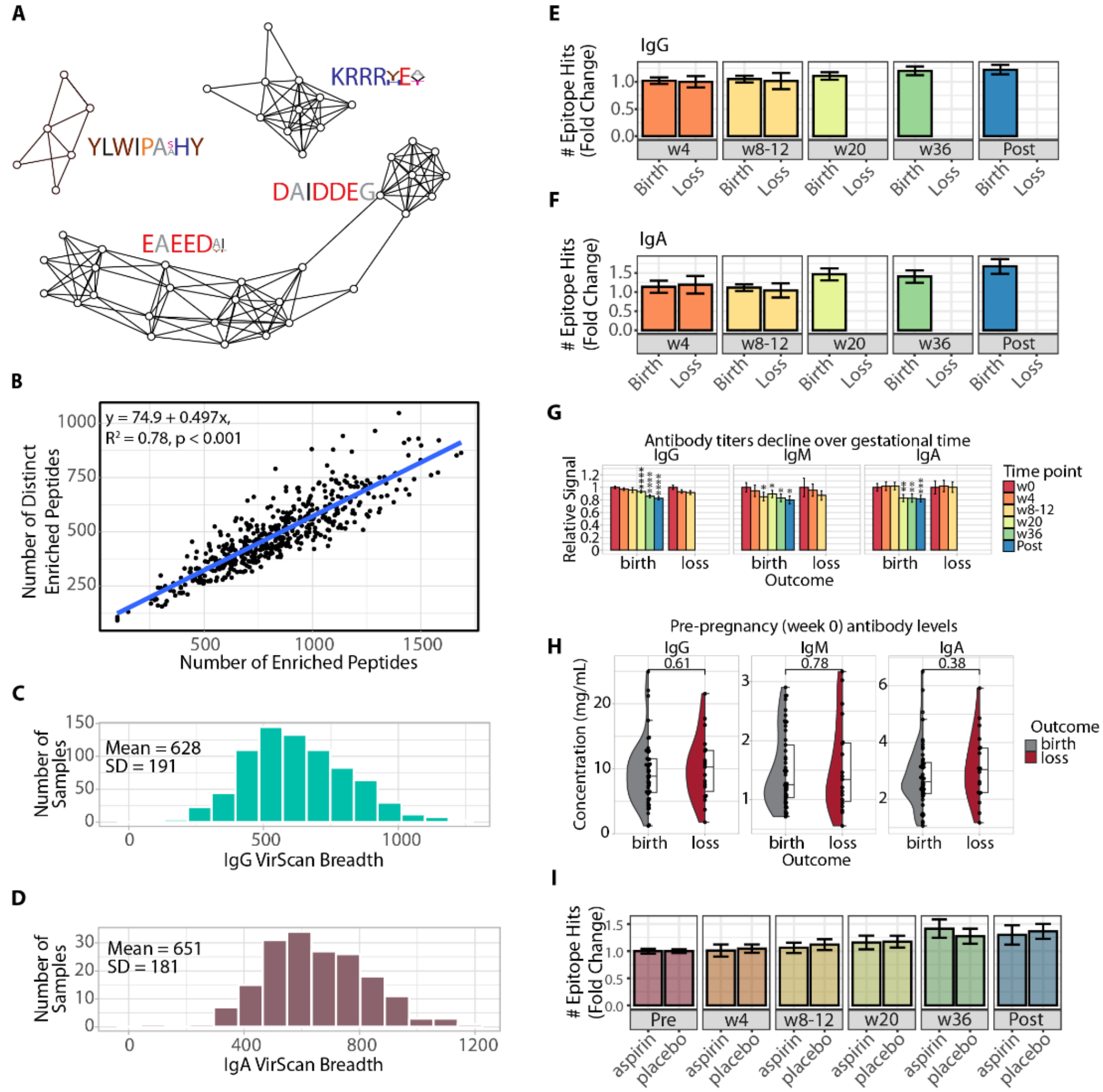

Fig. S2. Features of VirScan breadth quantification. Related to Figure 2.

(**A**) Network graph representation of relationship between peptides and epitopes from VirScan hits. Nodes represent selected peptides enriched by phage immunoprecipitation with the VirScan library for a particular donor sample. Edges represent peptides with sequence similarity. Sequence motif logos represent distinct epitopes, each comprised of a cluster of homologous peptides. (**B**) Scatterplot depicting per-sample non-collapsed number of enriched peptides on the x axis and post-collapse number of distinct enriched peptides on the y axis. The blue line represents a best-fit linear regression, corresponding to the equation, R-squared value, and p-value listed at the top left of the plot. (**C-D**) Distribution of the per-sample number of distinct enriched peptides ("breadth") per sample for (C) IgG and (D) IgA. (**E-F**) Bar plot showing fold change in VirScan breadth compared to pre-pregnancy baseline across gestational time for birth-outcome and loss-outcome pregnancies for (E) IgG and (F) IgA. Error bars indicate standard error of the mean (SEM). (**G**) ELISA measurements for IgG, IgM, and IgA concentration over gestational time, split by pregnancy outcome, normalized to pre-pregnancy baseline. Scores are normalized to the median week 0 scores per isotype and per pregnancy outcome. Error bars indicate SEM. Asterisks indicate significance for a Wilcox test for the null hypothesis that the median relative score at a given timepoint is equal to 1. * FDR < 0.1, ** FDR < 0.05, *** FDR < 0.01, **** FDR < 0.001. (H) Combined violin, box, and scatter plots showing the distribution of pre-pregnancy antibody titers in birth vs. loss outcome samples. Nominal p-values for t-tests comparing loss and birth are shown. (**I**) Bar plot showing fold change in VirScan breadth compared to pre-pregnancy baseline across gestational age for aspirin-receiving and placebo-receiving participants from the original EAGeR trial. Error bars indicate standard error of the mean (SEM).

Supplementary Figure S3

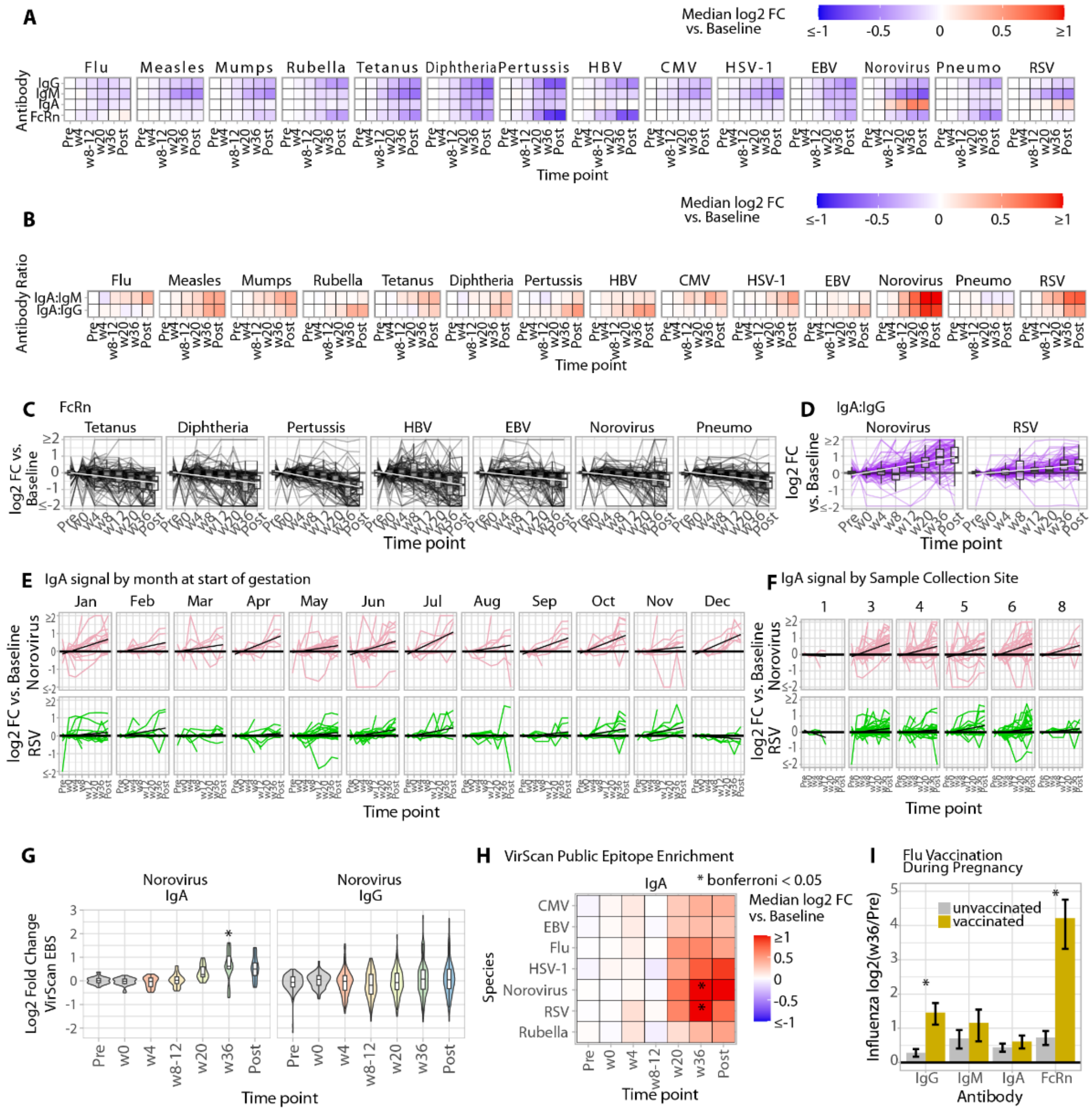

Fig. S3. Trends in Luminex antibody titers over gestational time. Related to Figure 3.

(**A-B**) Median fold change in Luminex MFI compared to pre-pregnancy baseline for antibody binding across all tested antigens. (A) for individual antibody isotypes, or for antibodies that bind to FcRn. (B) for the ratio of signal between two antibody types. (**C-F**) Line plot showing individual participant fold change compared to pre-pregnancy baseline for Luminex. Boxplots show median, 25th and 75th percentiles, and whiskers spanning up to 1.5 inter-quartile range. Trendlines show linear regression fit. (C) FcRn-binding antibodies for selected antigens of interest (D) the ratio of IgA fold change and IgG fold-change for norovirus and RSV (E) IgA:norovirus and IgA:RSV, stratified by month at the start of pregnancy (F) IgA:norovirus and IgA:RSV, stratified by physical location at which sample collection occurred. (**G**) Per-sample mean of IgA and IgG VirScan enrichment scores for 5 norovirus public epitope peptides, plotted as log2 fold change relative to pre-pregnancy baseline. Asterisks indicate Bonferroni-corrected p-value < 0.05. (**H**) Mean for public epitope enrichment score across all samples per time point for IgA for specified viruses. Per-donor public epitope scores were normalized by IgA concentration. Asterisks indicate Bonferroni-corrected p-value < 0.05. (**I**) Luminex influenza fold change at week 36 compared to pre-pregnancy baseline, stratified by antibody subset and whether influenza vaccination was administered during pregnancy. Asterisks indicate FDR < 0.05 for a t-test comparing log2 fold change scores of vaccinated and unvaccinated participants.

Supplementary Figure S4

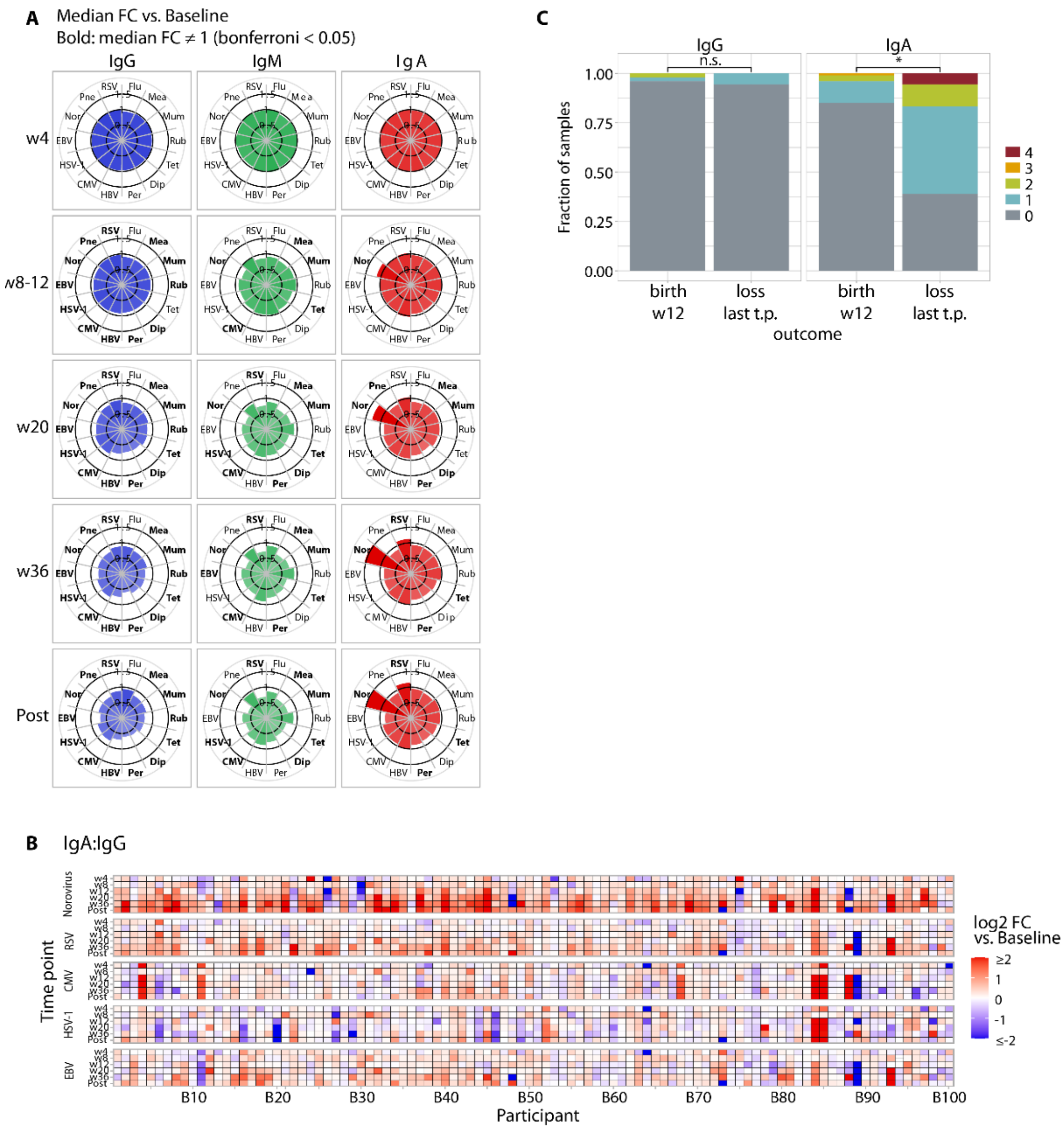

Fig. S4. Luminex antibody fold changes show IgA-specific trends. Related to Figures 3 and 4.

(A) Flower plot showing median fold change in Luminex MFI at compared to pre-pregnancy baseline. Bolded virus abbreviations indicate that a two-sided Wilcoxon rank-sum test yielded a Bonferroni-corrected p-value < 0.05 for the null hypothesis that median FC = 1. Abbreviations: Flu influenza, Mea measles, Mum mumps, Rub rubella, Tet tetanus, Dip diphtheria, Per pertussis, HBV Hepatitis B virus, CMV cytomegalovirus, HSV-1 herpes simplex virus 1, EBV Epstein-Barr virus, Nor norovirus, Pne streptococcus pneumoniae. **(B)** Heatmap showing the individual fold change scores for each participant (column) over gestational time (rows) for the ratio of IgA and IgG. **(C)** Stacked bar plot with colors indicating number of spikes to CMV, HSV-1, EBV, and/or Norovirus per sample. Bar plot expresses the fraction of samples with each number of spikes to these viruses, comparing the last time point available for pregnancies that ended in miscarriage to samples from birth outcome pregnancies that are matches for gestational time. Significance values indicate the FDRs for t-tests comparing per-isotype distributions of number of antiviral spikes in the specified loss- and birth-outcome samples (FDR *<0.05).

Supplementary Figure S5

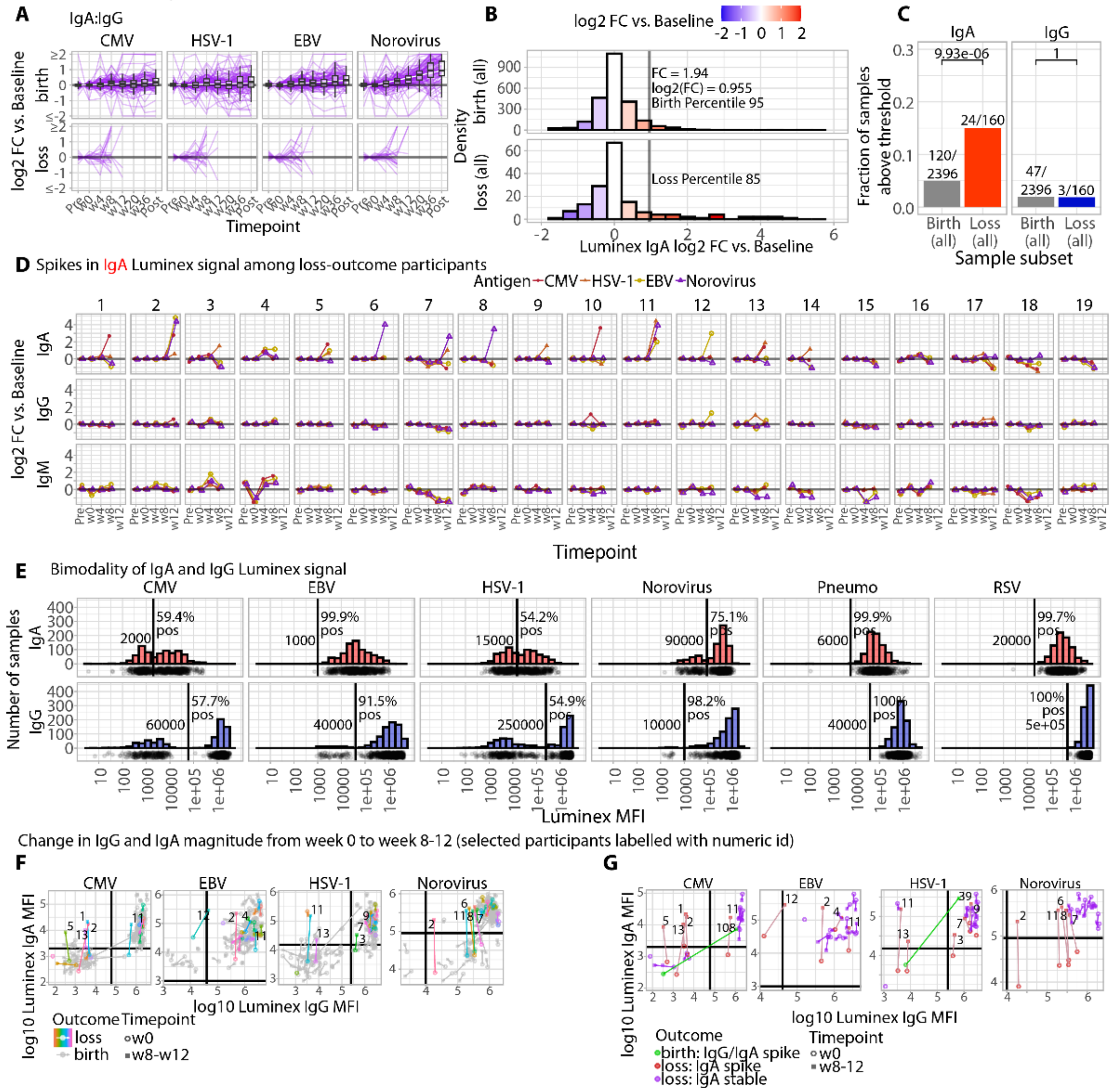

Fig. S5. IgA spikes precede miscarriage. Related to Figure 4.
(**A**) Ratio of IgA and IgG Luminex fold change relative to pre-pregnancy baseline for birth and loss outcome pregnancies for specified viruses. (**B**) Distribution of IgA fold-change vs. pre-pregnancy baseline for selected viruses of interest (CMV, HSV-1, EBV, and norovirus) for birth-outcome pregnancies at the week 4, week 8, week 12, week 20, week 36, and postpartum time points (top) and loss outcome pregnancies at the week 4, week 8, and week 12 time points (bottom). Vertical line indicates 95th percentile score for birth outcome (FC = 1.94). (**C**) Fraction of birth and loss samples from (B) scoring above FC of 1.94 for IgA (left) and IgG (right). Brackets are labeled with FDR values from Fisher's exact test. (**D**) Per-person fold change in Luminex antiviral titers for each loss outcome pregnancy for specified viruses for IgA, IgG, and IgM. Points are jittered horizontally slightly for improved visibility of overlapping data. (**E**) Distribution of Luminex MFI for IgA (red) and IgG (blue) for selected viruses. Vertical lines indicate threshold used for calling seropositivity with percentage of samples scoring as seropositive specified. (**F-G**) Scatterplot of Luminex IgG MFI on the x axis and IgA MFI on the y axis with lines connecting week 0 and week 12 samples per person for (F) birth outcome samples in grey and loss outcome samples in color. For pregnancies that terminated prior to week 12, the week 8 sample was used instead. (G) for loss outcome samples for which an IgA spike is observed in red, loss outcome samples for which an IgA spike is not observed in purple, and selected birth outcome samples with suspected true infection in green.

Supplementary Figure S6

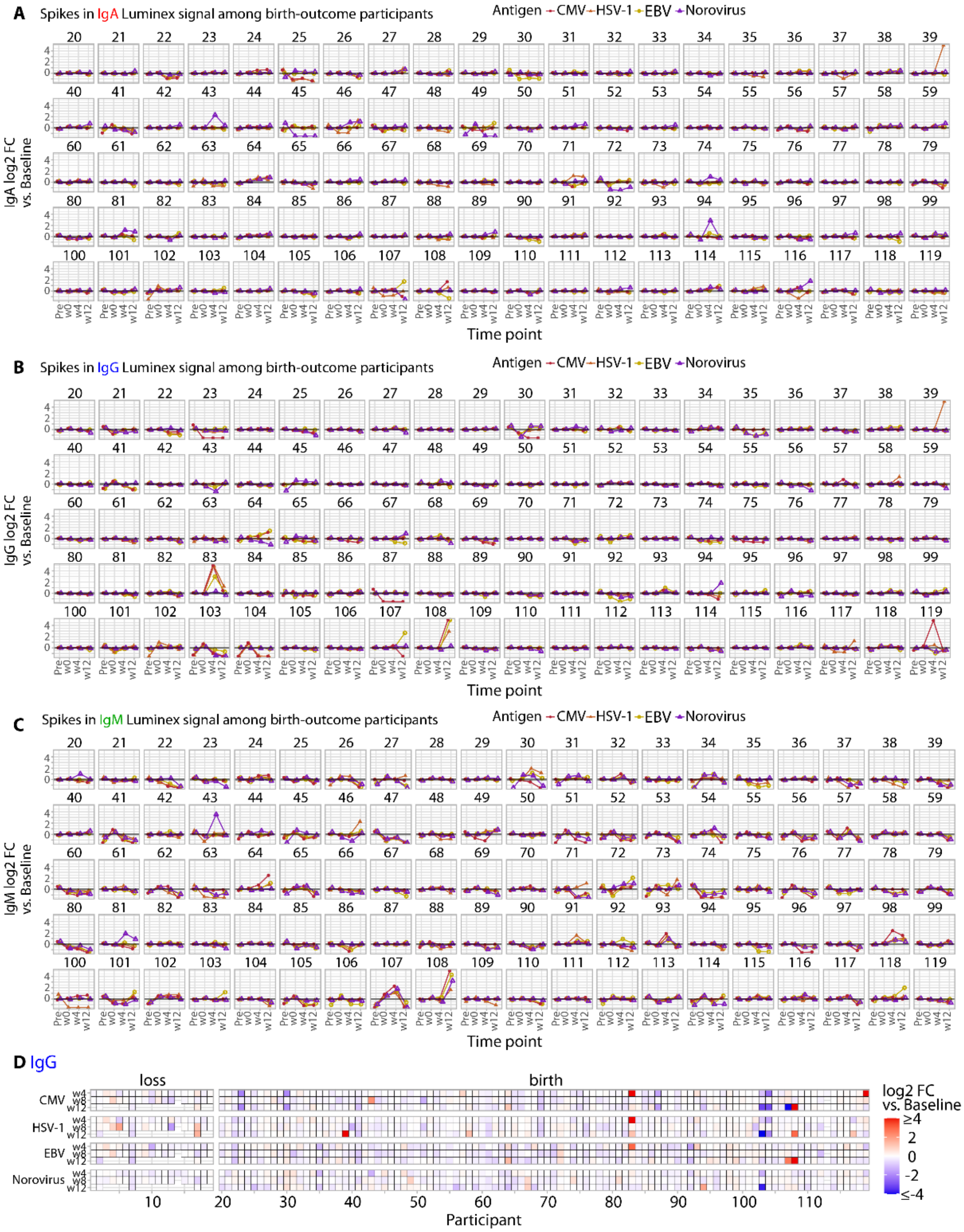

Fig. S6. Multi-isotype antiviral Luminex fold change. Related to Figure 4.

(**A-C**) Per-person fold change in Luminex antiviral titers for each birth outcome pregnancy for CMV, HSV-1, EBV, and norovirus for (A) IgA (B) IgG and (C) IgM. **(D)** Heatmap splitting by pregnancy outcome showing individual log2 fold change compared to pre-pregnancy baseline for Luminex antibody binding signal for selected viruses of interest for IgG.

Supplementary Figure S7

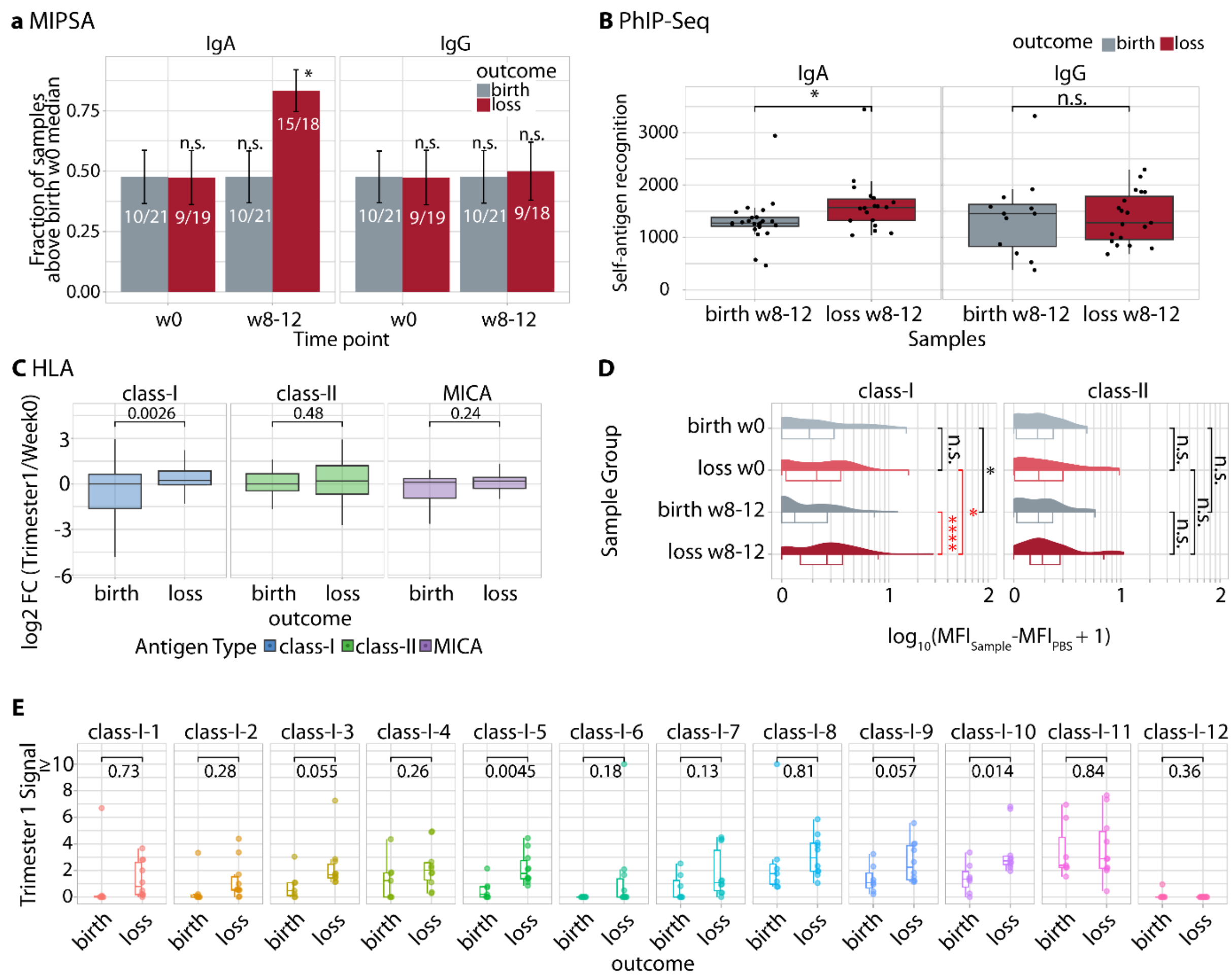

Fig. S7. Enhanced self-antigen recognition detected preceding miscarriage. Related to Figures 4 and 5. (**A**) Bar plots showing the fraction of samples with MIPSA self-antigen recognition higher than the median week 0 MIPSA self-antigen recognition for that isotype. Error bars show the standard deviation from 1000 bootstrapped samplings of each subgroup. Asterisk indicates FDR < 0.05 for a Fisher's exact test comparing the number of samples above and below the median score for birth outcome week 0 and the subgroup of interest. **(B)** Comparison of the number of peptides that score as enriched from PhIP-Seq for IgA (left) and IgG (right) using a human peptidome library for samples from the last time point collected prior to miscarriage or samples from birth outcome pregnancies, matched for gestational age. Asterisks indicate FDR < 0.05 for a Wilcoxon test comparing self-antigen recognition scores from birth and loss outcome pregnancies. (**C)** Box plots showing the distribution of IgA LabScreen Mixed scores as a ratio of each participant's trimester 1 score and their pre-pregnancy score. For each participant, there are 12 points for class I, 5 for class II, and 2 for MHC Class I-Related Chain A (MICA), corresponding to the number of distinct bead readouts included in the assay. Statistics indicate nominal p-values from a Wilcoxon test comparing the distributions of ratios for birth and loss-outcome pregnancies. **(D)** Distributions of background-subtracted scores across all class I (repeated from Fig. 5 for comparison) and class II antigens and samples, stratified by antigen class, pregnancy outcome, and time point. Significance indicators indicate the results of Wilcoxon tests comparing each group of scores (p ****<0.0001, *<0.1, n.s. not significant). Red brackets with asterisks indicate an increase in anti-HLA signal, either over time or associated with miscarriage, while the black bracket and asterisk indicate a reduction in anti-HLA signal over time in the birth cohort. **(E)** Birth and loss outcome LabScreen for each of the 12 separate class I read outs. Brackets are labeled with nominal p-values from a Wilcoxon test comparing birth and loss outcome scores.

Supplementary Figure S8

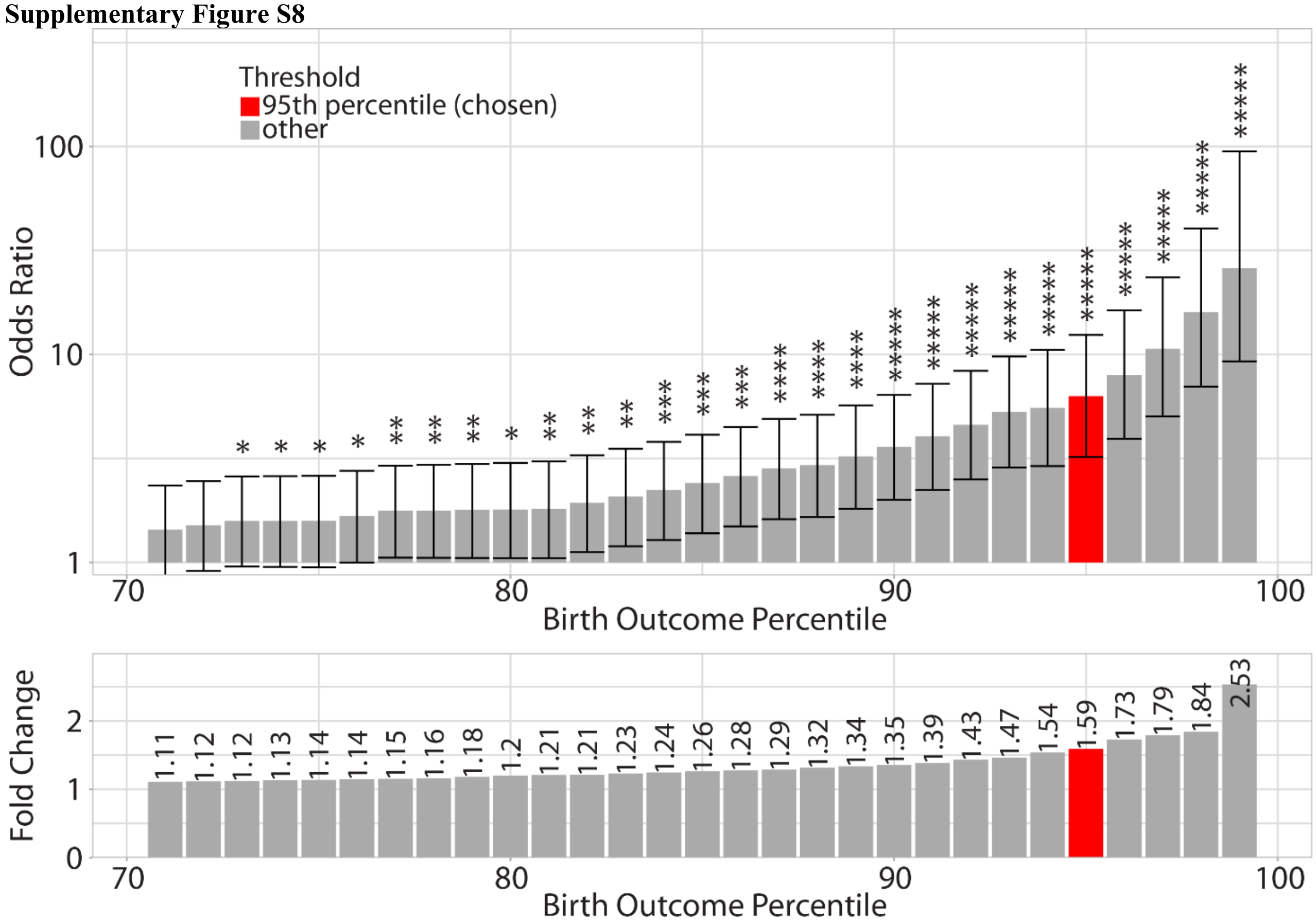

Figure S8. **IgA STORM is robustly associated with miscarriage across a wide range of thresholds. Related to Figure 4**. Thresholds for IgA fold-change ranging from fold increase of 11% (71st percentile of week 12 birth outcome scores) up to a fold increase of 153% (99th percentile of week 12 birth outcome scores) were evaluated for discrimination of pregnancy outcome (birth, miscarriage). On top, odds ratios are calculated, with y axis values on a log scale. Error bars indicate the 95% confidence interval for odds ratio. Asterisks indicate the significance level emerging from a Fisher's exact test for each threshold (* $p < 0.1$, ** $p < 0.05$, *** $p < 0.01$, **** $p < 0.001$, ***** $p < 0.0001$). Threshold chosen in the study of 95th percentile of birth outcome scores is highlighted in red.

Supplementary Figure S9

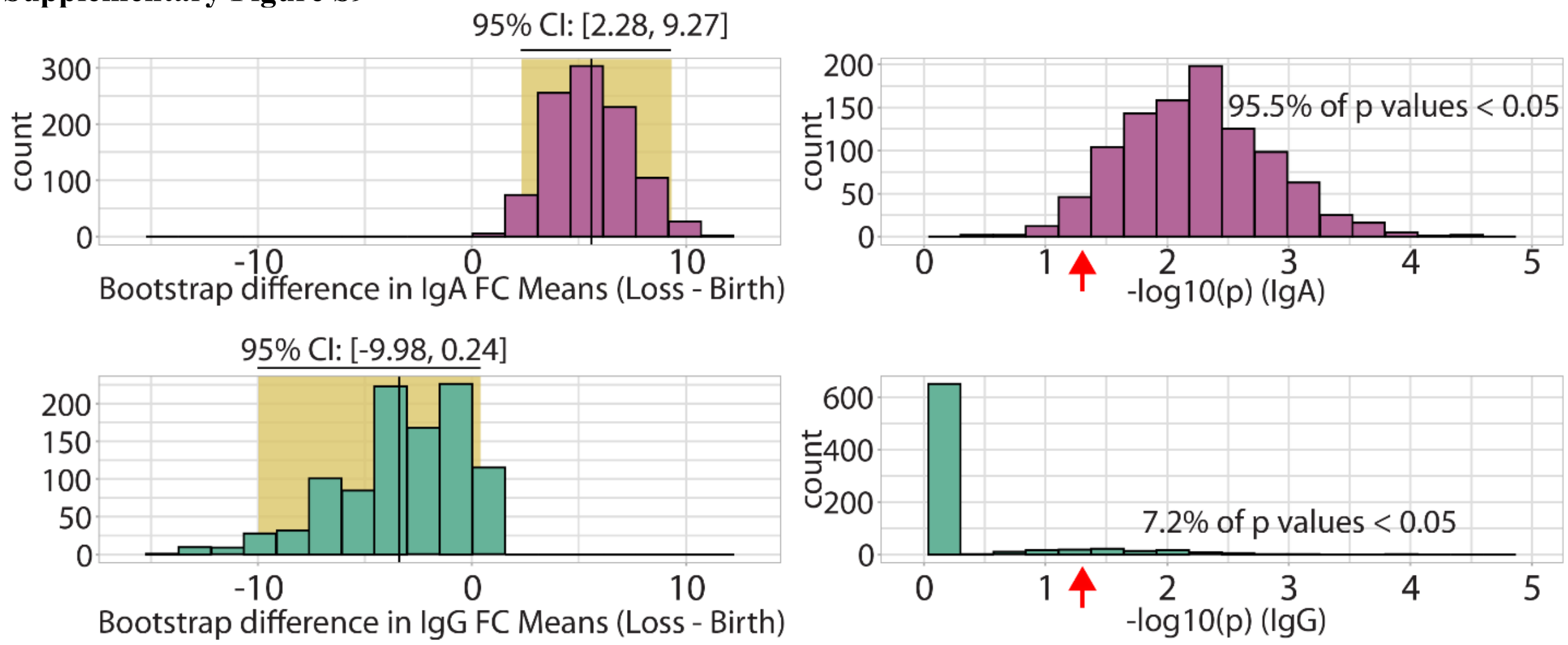

Figure S9. Bootstrapping evaluation of IgA STORM. Related to Figure 4. For IgA (top, pink) and IgG (bottom, teal), random sampling with replacement was performed 1,000 times. In each sampling iteration, for each isotype, paired data from week 0 and the week 12 time point was considered (or week 8 if week 12 sample was not collected/available). Across the four STORM antigens (CMV, EBV, HSV-1, and norovirus), the maximum fold change was calculated for each participant. Then, the mean maximum fold change was calculated for sampled participants, stratified by pregnancy outcome (miscarriage or live birth). The difference in mean fold change between pregnancies that end in miscarriage and pregnancies that end in live birth was computed for each iteration, and the resultant distribution is plotted in the two plots on the left. Text above the plot and the yellow rectangular area indicate the 95% confidence interval defined by these distributions. The vertical bars on these two left-side plots indicate the difference in means observed in the study. On the right, for each iteration, a t-test was performed to compare the distribution of fold change scores observed in pregnancies that ended in miscarriage and those that ended in live birth. The distribution of these p-values is plotted, and the red arrow below the x-axis indicates a p-value of 0.05 for reference.

Supplementary Figure S10

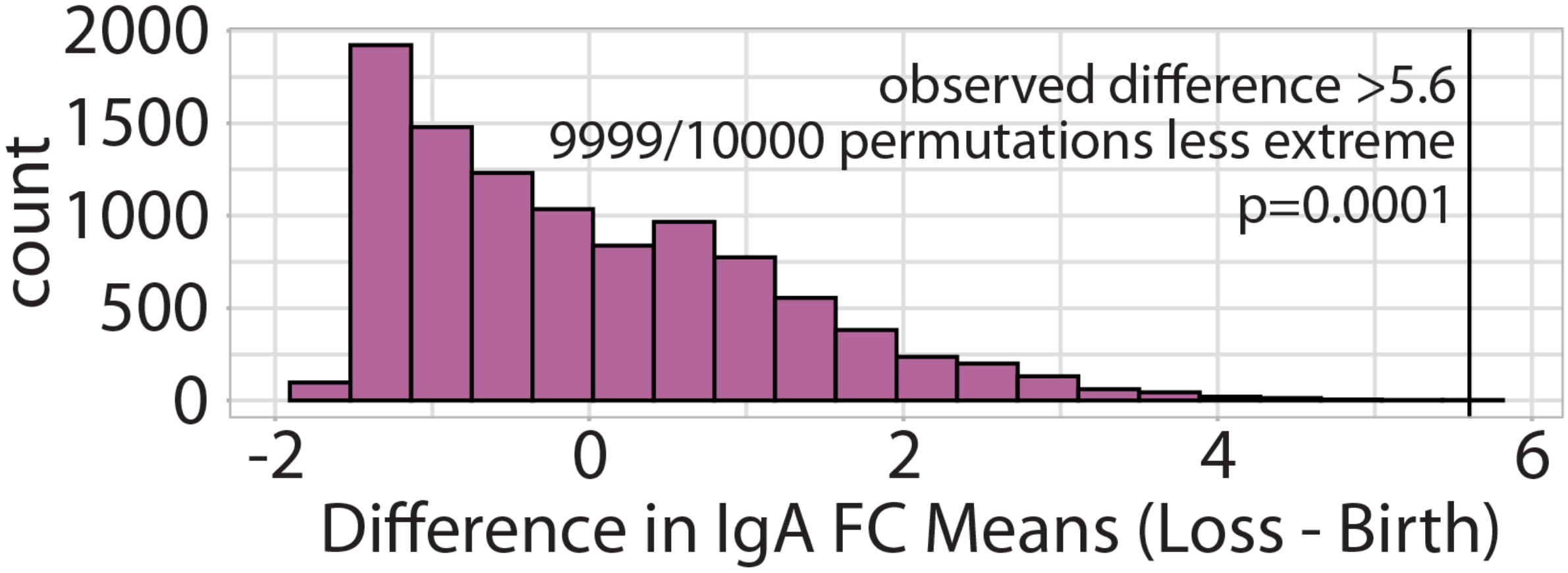

Figure S10. Permutation test evaluation of the significance of IgA STORM. Related to Figure 4. Paired IgA data was considered from week 0 and week 12 (or week 8 if week 12 sample was not collected/available). For all 119 participants, the maximum fold change was calculated across the four STORM antigens (CMV, EBV, HSV-1, and norovirus). Then, repeated for 10,000 iterations, metadata labels of whether each participant's pregnancy ended in live birth or miscarriage were randomized and recorded. From each randomized set of metadata labels, the mean STORM FC score was calculated for pregnancies labeled as ending in miscarriage and those labeled as ending in live birth. The histogram represents the distribution of difference in fold change means obtained from these randomly permuted metadata. The vertical line indicates the difference in means actually observed in the study (5.6), which correspond to a permutation p-value of 0.0001.

Supplementary Figure S11

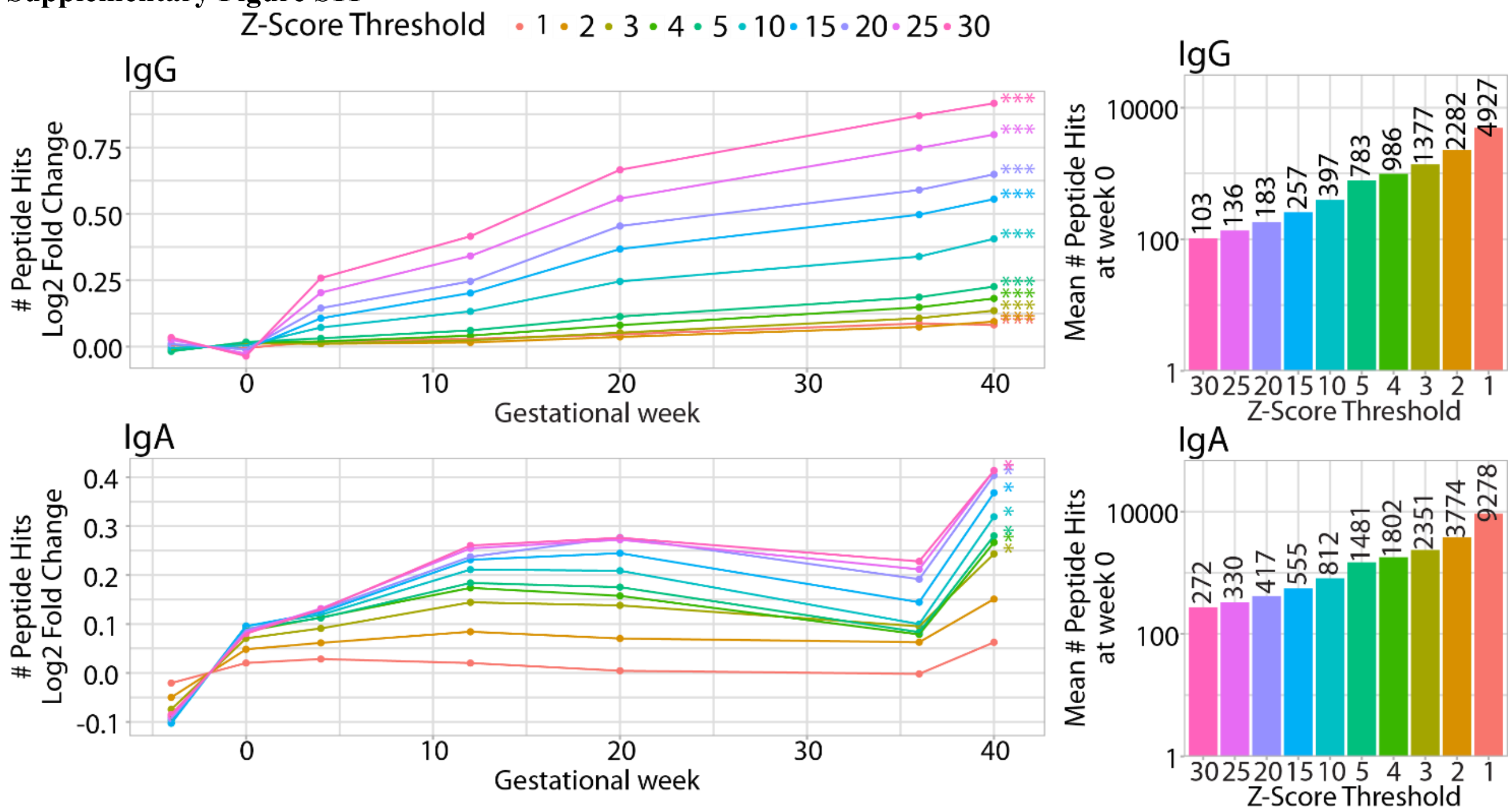

Figure S11.Antibody breadth increase is observed across a broad range of z-score thresholds. Related to Figure 2. (left) Change in antibody breadth over the observed gestational time course at different z-score thresholds for IgG on top and IgA below. Z-score threshold legend is at the top. The post-partum time point is coded as week 40. Asterisks to the right indicate significance of a Wilcoxon test testing the null hypothesis that the median fold change in antibody breadth at the post-partum time point is equal to 0 (log2 FC equal to 1) (*<0.05, ***<0.001). (right) bar plots indicating the average number of peptide hits at week 0 for each Z-score threshold for IgG on top and IgA below.

Supplementary Figure S12

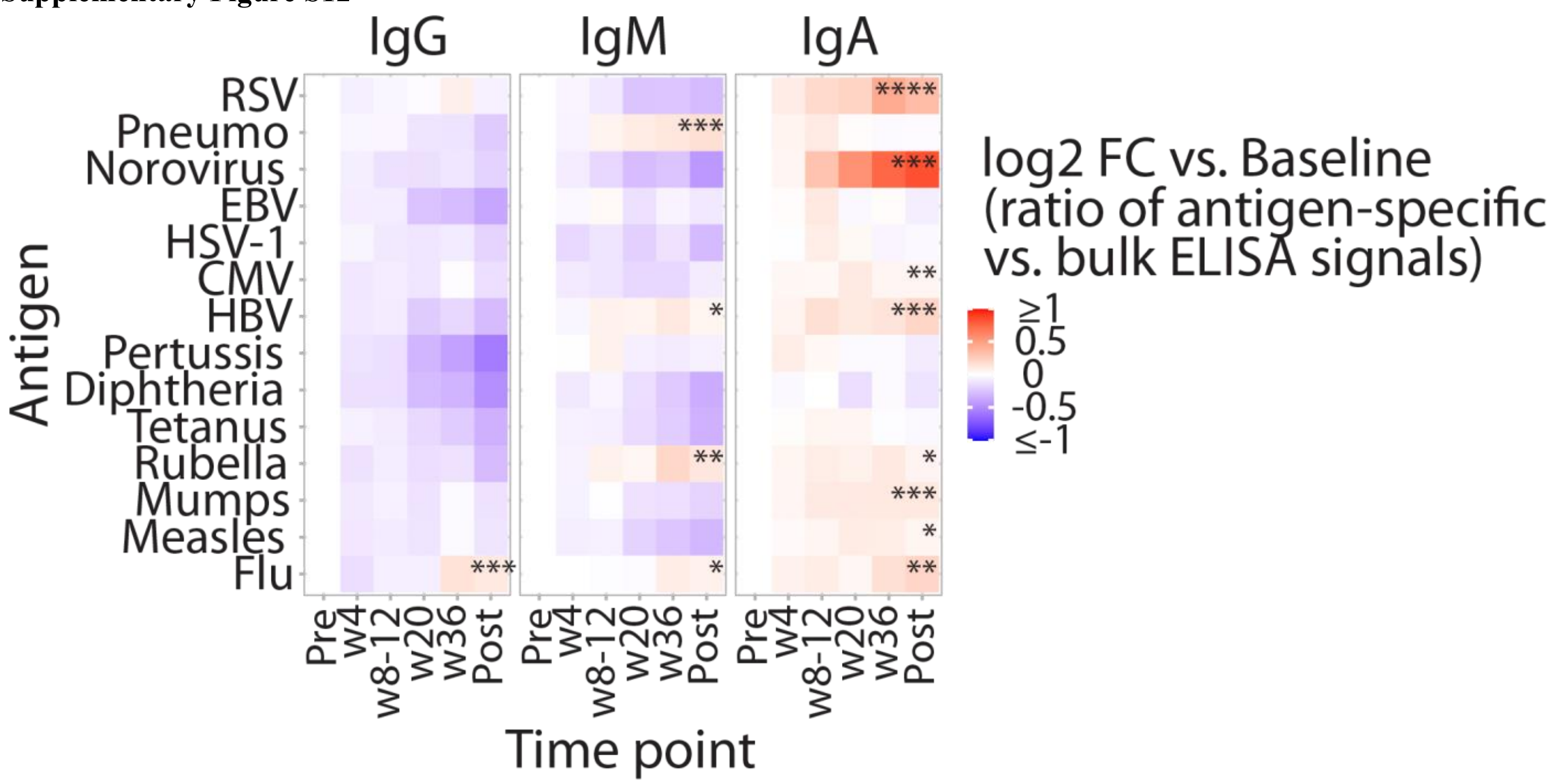

Figure S12. Median Luminex fold change vs. pre-pregnancy baseline, normalized to bulk isotype concentration by ELISA. Related to Figure 3. Asterisks indicate the BH-corrected significance level for one-sided t-test for the null hypothesis that post-partum fold-change in antigen-specific signal normalized to total isotype signal is less than or equal to 1 (*<0.05, **<0.01, ***<0.001, ****<0.0001).

Supplementary Figure S13

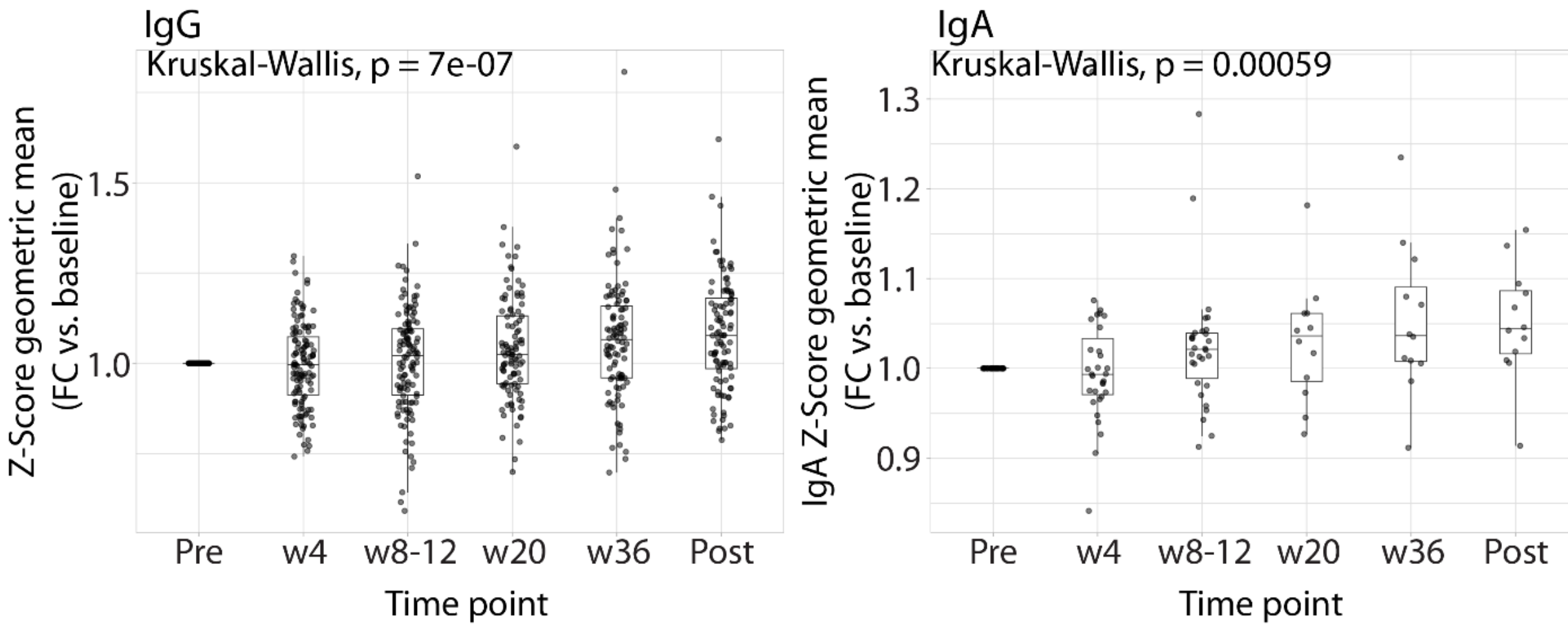

Figure S13. **VirScan hit magnitude increases over gestational time. Related to Figure 2**. Boxplots overlayed with horizontally jittered scatterplots representing the distribution of the geometric mean of Z-score magnitudes across the cohort and over gestational time for IgG (left) and IgA (right). For each isotype, text on top indicates the p-value from a Kruskal-Wallis test testing that the distributions at all time points are drawn from the same underlying distribution.

Supplementary Figure S14

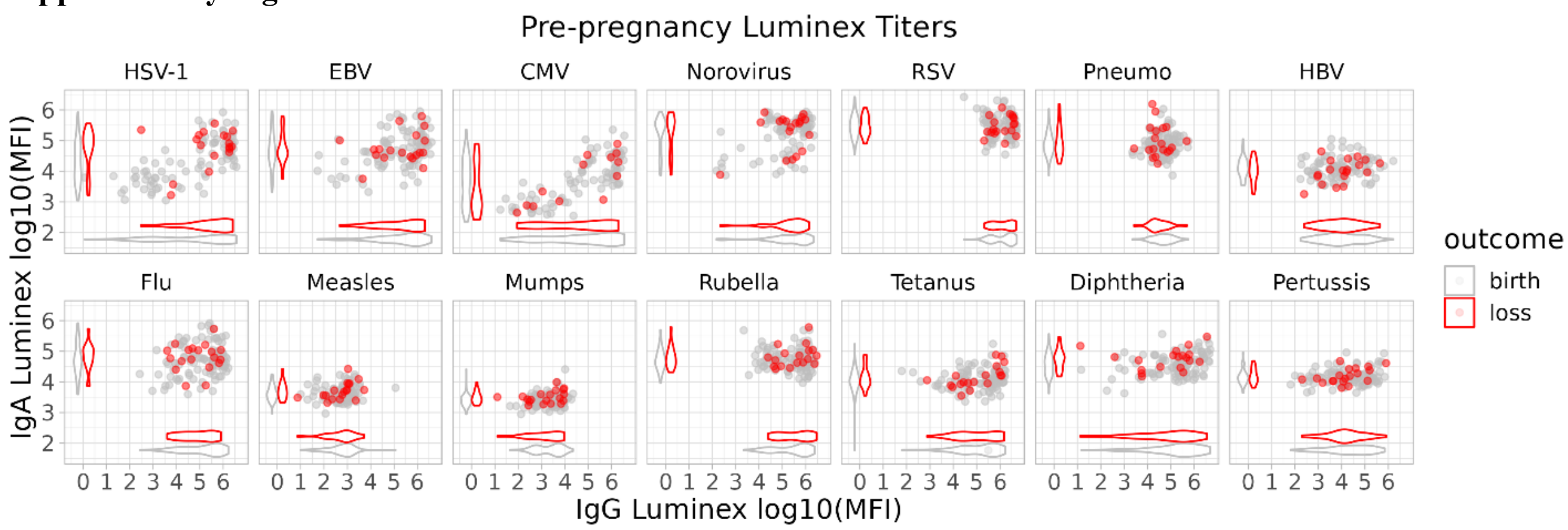

Figure S14. Distribution of pre-pregnancy raw Luminex MFI is not significantly different in pregnancies that end in miscarriage and those that end in live birth. Related to Figure 4. Raw Luminex MFIs are plotter for IgG (x) and IgA (y) for birth-outcome pregnancies (grey) and miscarriage-outcome pregnancies (red). Violin plots to the left and bottom of each scatterplot indicate the distribution of scores by pregnancy outcome for IgA (left) and IgG (bottom). Data is separated for each of the 14 antigens tested by Luminex. None of the antigens showed a statistically significant difference between pregnancy outcomes by t-test.

Table S4. Commercial antigens used for Luminex assay. Related to Figures 3 and 4.

Commercial protein antigens purchased for Luminex serological profiling. The first three columns indicate the supplier name, the product name, and the product catalog number when available. Items with the same label in the "Pool" column were mixed together prior to coupling to Luminex beads.

**Vendor**	**Item**	**Catalog #**	**Pool**
List Biological Laboratories	Pertussis toxin (50ug)	180	
Native Antigen (Vendor: Cedarlane)	Tetanus Toxoid, Recombinant Heavy Chain Fragment C (100ug)	REC31801-100	
Immune Tech	EBV gp350/220 (100ug)	IT-005-035p	
Immune Tech	HA1 (A/New Caledonia/20/1999) (100ug)	IT-003-001p	A
Immunetech	HA1(B/Phuket/3073/2013)	IT-003-B11p	A
Immunetech	HA1(H1N1)(A/Michigan/45/2015)	IT-003-00105p	A
BioRad Antibodies	Measles - Endmonston	PIP013	
ATCC	Pneumococcal Polysaccharide Powder Type 19A	301-X	B
ATCC	Pneumococcal Polysaccharide Powder Type 7F	108-X	B
Cedarlane	Diphtheria toxin	DIP-TNL	
BioRad Antibodies	Mumps - Enders	PIP014	
Vendor: Cedarlane	Norovirus VP1 GII.4	REC31620-100	
BioRad Antibodies	Rubella HPV-77	PIP044	
Immune Tech	HSV-1 gD(Ectodomain)	IT-005-055p	
Aviva systems biology	HBV HBsAg Adw	OPPA00881	
Sino Biological	Human cytomegalovirus (HCMV) Glycoprotein B / gB Protein (His Tag)	10202-V08H1	
NIH	RSV A/B Pre F Fusion Protein		C
NIH	RSV A/B Post F Fusion Protein		C
